# Self‐Assembly of Aminocyclopropenium Salts: En Route to Deltic Ionic Liquid Crystals

**DOI:** 10.1002/anie.202000824

**Published:** 2020-04-07

**Authors:** Juri Litterscheidt, Jeffrey S. Bandar, Max Ebert, Robert Forschner, Korinna Bader, Tristan H. Lambert, Wolfgang Frey, Andrea Bühlmeyer, Marcus Brändle, Finn Schulz, Sabine Laschat

**Affiliations:** ^1^ Institute of Organic Chemistry University of Stuttgart Pfaffenwaldring 55 70569 Stuttgart Germany; ^2^ Department of Chemistry Colorado State University Fort Collins CO 80523 USA; ^3^ Department of Chemistry & Chemical Biology Cornell University 122 Baker Laboratory Ittaca NY 14853 USA; ^4^ Department of Chemistry Columbia University New York NY 10027 USA

**Keywords:** aminocyclopropenium ions, aromatic ions, nanosegregation, self-assembly, X-ray diffraction

## Abstract

Aminocyclopropenium ions have raised much attention as organocatalysts and redox active polymers. However, the self‐assembly of amphiphilic aminocyclopropenium ions remains challenging. The first deltic ionic liquid crystals based on aminocyclopropenium ions have been developed. Differential scanning calorimetry, polarizing optical microscopy and X‐ray diffraction provided insight into the unique self‐assembly and nanosegregation of these liquid crystals. While the combination of small headgroups with linear *p*‐alkoxyphenyl units led to bilayer‐type smectic mesophases, wedge‐shaped units resulted in columnar mesophases. Upon increasing the size and polyphilicity of the aminocyclopropenium headgroup, a lamellar phase was formed.

## Graphical abstract

**Cyclopropenium‐based liquid crystals** form lamellar or columnar mesophases, depending on the substitution of the head group and the number of peripheral chains. The effective volume of headgroup versus hydrophobic part determines the mesophase type. These compounds bridge the gap between low molecular weight organocatalysts and polymeric electrolytes based on cyclopropenium.

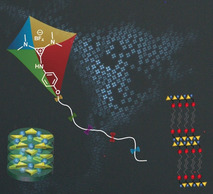

## Introduction

**Figure 1:**
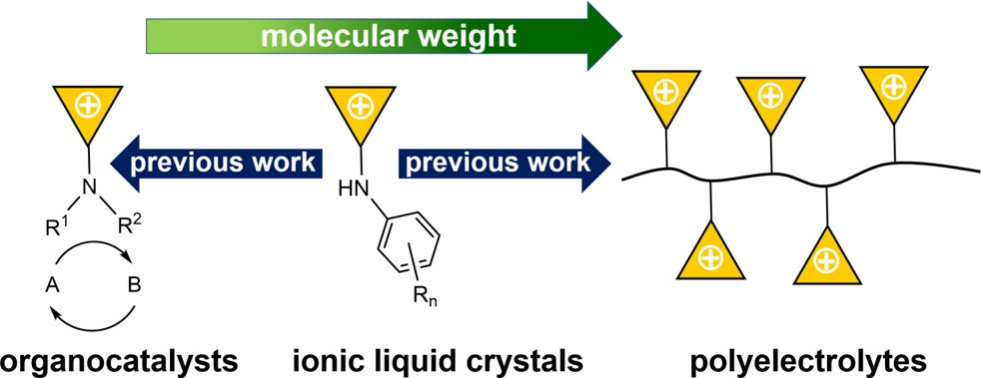
Different types of cyclopropenium based materials. ILCs with a strong tendency for self‐assembly are the link between low molecular weight organocatalysts (isolated molecules) and polyelectrolytes (polymers).

Since the groundbreaking synthesis of triphenylcyclopropenium ions by Breslow in 1957, interest in these small non‐benzoid aromatic rings has been steadily rising.^1^ The unique combination of aromatic stability with ring strain, and the possibility to tailor the physical and chemical properties using the substituents attached to the cyclopropenium, provides highly interesting materials from both theoretical and experimental or application‐oriented perspectives.^2^, ^3^, ^4^ Among the differently substituted cyclopropenium cations, the aminocyclopropenium ions first described by Yoshida in 1971,^5^ have received special attention.^6^ Most recent work has focused on their use as phase transfer, Lewis acid or organocatalysts,^7^, ^8^, ^9^, ^10^, ^11^, ^12^, ^13^, ^14^, ^15^, ^16^ electrophotocatalysts,^17^ ligands for catalytic metal complexes,^18^, ^19^, ^20^ ionic liquids,^21^, ^22^, ^23^, ^24^, ^25^ persistent radical cations,^26^ redox active polymers for redox flow batteries,^27^, ^28^, ^29^, ^30^, ^31^, ^32^ fluorescent materials,^33^, ^34^, ^35^ aromatic cations in hybrid halide perovskites,^36^ biologically active compounds such as transfection agents,^37^, ^38^, ^39^ and nanoparticles.^40^, ^41^ Surprisingly, the self‐assembly of cyclopropenium compounds into liquid crystalline phases has not been reported.^42^ We hypothesized that mesomorphic self‐assembly should be promoted by the planarity and charge delocalization of the aminocyclopropenium ion, which resembles an extended, or “deltic” guanidinium ion.^43^ Indeed, guanidinium ions are known building blocks that support mesomorphism of ionic liquid crystals (ILCs).^44^, ^45^, ^46^, ^47^ ILCs are anisotropic fluids with long‐range orientational order caused by Coulombic interactions between cationic headgroups and counterions, nanosegregation between immiscible parts (that is, an ionic headgroup, rigid core, and lipophilic tail), minimization of free volume complemented by van der Waals interactions, π—π, and hydrogen bonding interactions.^44^, ^45^ As ILCs have been reported to serve as a link between neutral liquid crystals and polyelectrolytes,^48^ insight into the structure–property relationships of aminocyclopropenium‐derived ILCs should enable better tuning of the corresponding polyelectrolytes carrying aminocyclopropenium units for both batteries and fuel cells,^27^, ^28^, ^29^, ^30^, ^31^, ^32^, ^49^, ^50^ as well as gene‐delivery vectors^51^, ^52^ (Figure 1). The manipulation of the three substituents of the deltic guanidinium headgroup of aminocyclopropenium‐based LCs should not only provide a general understanding and tailoring of the bulk self‐assembly of these aromatic cations, but should also lead to ordered oligomeric aminocyclopropenium‐salt‐based catholytes in redox flow batteries.^27^, ^28^, ^29^, ^30^, ^31^, ^32^ Moreover, the recently reported propensity of cyclopropenium cations to form closely bonded dimers with short π–π distances of only 3.22 Å (as compared to 3.3–3.8 Å for other arenes)^53^ should further enforce mesomorphic self‐assembly. Such cyclopropenium‐derived liquid crystals would complement the series of known liquid crystalline Hückel aromatic cyclopentadienyl anions and benzenes,^54^ providing insight into the requirements of 3‐membered π‐systems to form stable mesophases.

Herein we report, for the first time, that ILCs can be obtained from aminocyclopropenium derivatives, whose phase geometry and temperature range is controlled by the headgroup.

## Results and Discussion

### Synthesis of aminocyclopropenium salts and related compounds

**Scheme 1:**
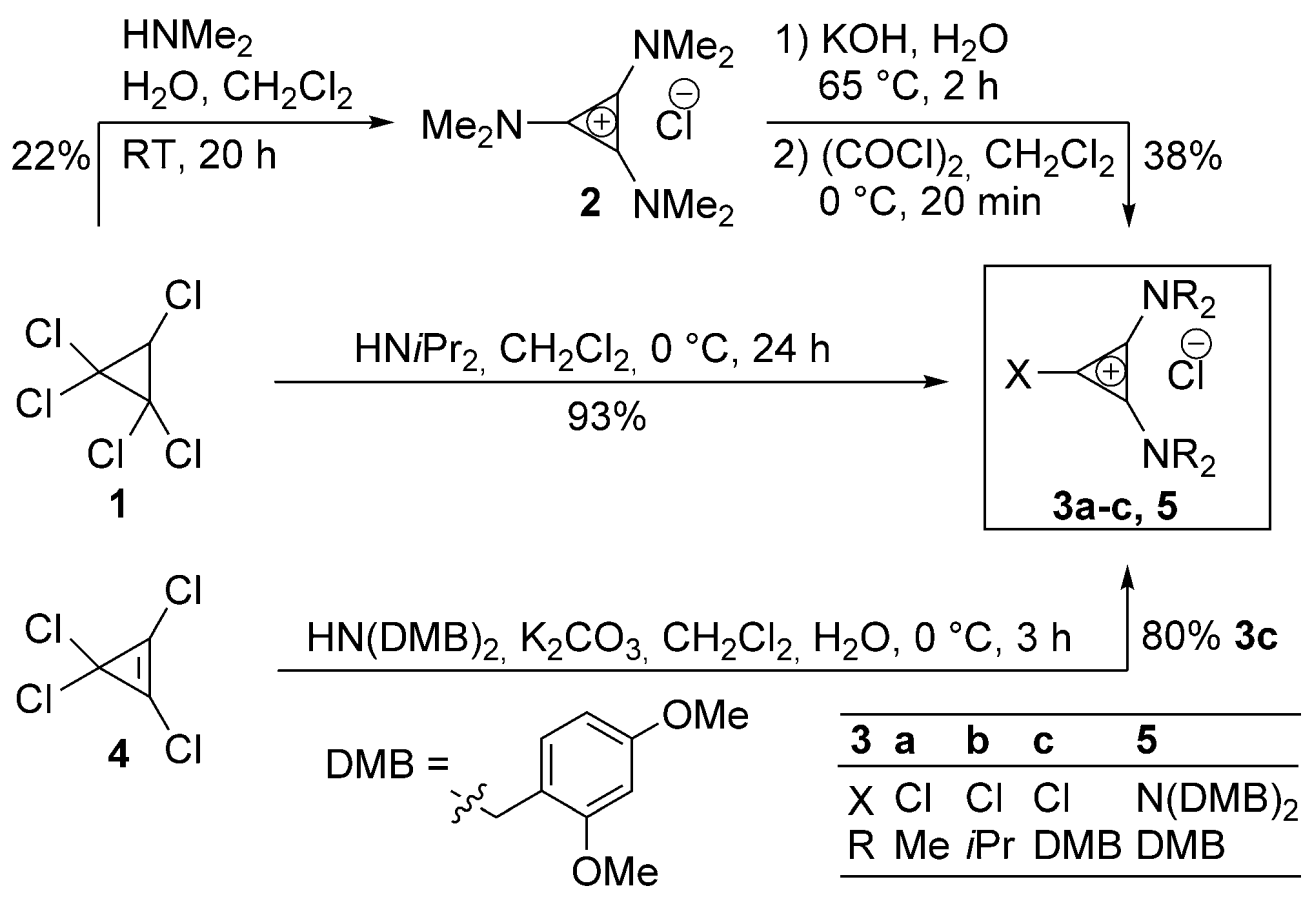
Synthesis of target cyclopropenium transfer reagents **3 a**–**c**. Key: dimethoxybenzyl (DMB).

At the outset of our study, we chose a 4‐alkoxyphenyl and wedge‐shaped 4‐[(3,4,5‐trialkoxybenzoyl)oxy]phenyl moieties as different mesogenic cores for attachment to the aminocyclopropenium headgroups because these core units are known to promote mesophase formation in guanidinium ILCs.^55^, ^56^ The synthesis of the cyclopropenium transfer reagents **3 a**–**c** is shown in Scheme 1.

Compound **1** was converted into tris(dimethylamino)cyclopropenium chloride **2**, according to a procedure by Yoshida.^5^ Subsequent saponification of **2** using a method by Curnow,^25^, ^56^ followed by treatment with oxalyl chloride, yielded the desired target **3 a** in 38 % yield over two steps. The corresponding diisopropylamino‐substituted **3 b** was obtained in 93 % yield by nucleophilic displacement of **1** with 2 equivalents of diisopropylamine, following the method by Yoshida.^5^, ^57^ Reacting tetrachlorocyclopropene **4** with bis(2,4‐dimethoxybenzyl)amine in CH_2_Cl_2_ in the presence of K_2_CO_3_ gave reagent **3 c** 43 in 80 % yield with 20 % of the separable threefold‐substituted cyclopropenium chloride **5**.

**Scheme 2:**
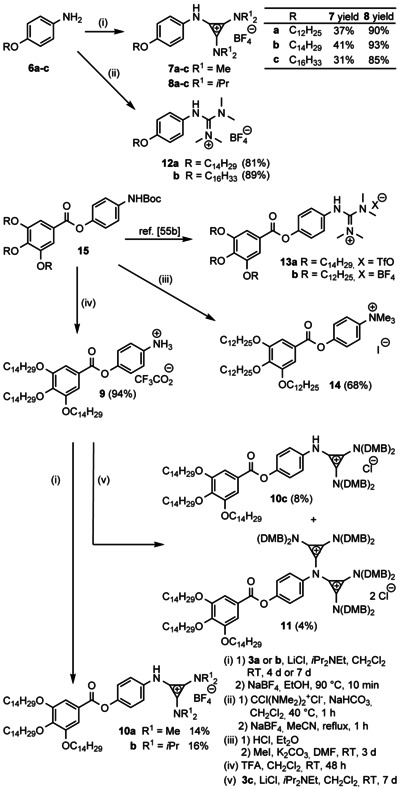
Synthesis of diamino cyclopropenium salts **7** and **10** with different mesogenic core units (for detailed synthesis of salts **8 a**–**c** see the Supporting Information, Scheme S3). Guanidinium ILCs **12**, known **13**,55b and ammonium salt **14** were synthesized for comparison. Key: trifluoroacetic acid (TFA), *N*,*N*‐dimethylformamide (DMF).

To access cyclopropenium ILCs **7**, 4‐alkoxyanilines **6 a**–**c** 55a were treated with cyclopropenium chloride **3 a** in the presence of LiCl and Hünig's base in CH_2_Cl_2_ for four days. After chromatographic purification, the resulting product was submitted to salt metathesis with NaBF_4_ in EtOH to yield the cyclopropenium tetrafluoroborates **7 a**–**c** in 31–41 % yield (Scheme 2). Under similar conditions, ammonium trifluoroacetate **9** derived from the corresponding Boc‐protected precursor55b was treated with reagents **3 a** or **3 b** to provide the wedge‐shaped tetrafluoroborates **10 a** or **10 b** in 14 % and 16 % yields, respectively (Scheme 2). Presumably, the acylated phenol of **9** reduces the nucleophilicity of the nitrogen atom relative to compounds **6**–**8**, thus leading to diminished yields of **10**. It should also be emphasized that efforts undertaken to optimize the yields were not extensive.

To assess the influence of the unique aminocyclopropenium headgroup on the liquid crystalline self‐assembly process, structurally related guanidinium salts **12 a** and **12 b**, and known compound **13 a** and **13 b** 55b with a similar core unit and side chain lengths as well as the same counterion, were chosen as reference compounds (Scheme 2). As described earlier for related ILCs,55a guanidinium tetrafluoroborates **12 a** and **12 b** were prepared from the corresponding guanidinium chlorides through salt metathesis.

Furthermore, the trimethylammonium salt **14** was prepared to compare this ILC, carrying a small spherical headgroup, with derivatives carrying much larger planar delocalized guanidinium or aminocyclopropenium headgroups. Compound **14** was obtained from **15** in 68 % by acidic removal of the *N*‐Boc group and methylation with an excess of methyl iodide.

The reaction of trifluoroacetate **9** with cyclopropenium salt **3 c** afforded the desired aminocyclopropenium chloride **10 c** in 8 % yield. The disubstituted derivative **11** was also isolated in 4 % yield; its formation was unexpected because of the bulky substituents, although similar bis(cyclopropenium)‐substituted amines carrying *i*Pr groups have been reported in the literature.^58^, ^59^

**Figure 2:**
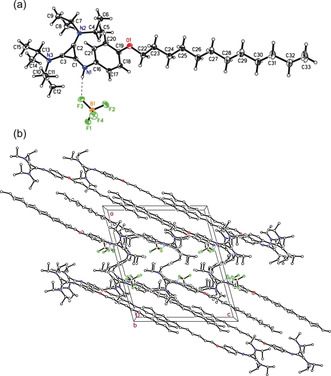
a) ORTEP drawing of the structure of 1,2‐bis(diisopropylamino)cyclopropenium tetrafluoroborate **8 a** in the solid state and b) packing diagram viewed along the *b* axis.

Fortunately, single crystals of **8 a** with a C_12_ side chain were obtained, which were suitable for X‐ray crystal structure analysis (Figure 2). Derivative **8 a** is oriented in a linear extended all‐*trans* conformation in the solid state. The distance N1H1⋅⋅⋅F3 between the NH as H‐donor and F3 of the tetrafluoroborate anion as H‐acceptor is 2.05 Å. A weak interaction between C21H21, C17H17, and C6H6 as H‐donors, and the F atoms of BF_4_ as H‐acceptor with H⋅⋅⋅F distances ranging between 2.36 Å and 2.55 Å, were found. Interdigitation of the alkyl chains is visible in the cell plot. A hydrophobic interaction between the chains, which are stacking perpendicular to the *ac* diagonal, was not observed (Supporting Information).^60^

### Mesomorphic properties of cyclopropenium salts and related compounds

The mesomorphic properties of the aminocyclopropenium salts **7**, **8**, **10**, and ammonium salt **9** were investigated by differential scanning calorimetry (DSC), polarizing optical microscopy (POM), and X‐ray diffraction (XRD). The corresponding guanidinium ILCs **12**, known **13**,55b and ammonium salt **14** (Scheme 2) were considered for comparison. The DSC results are summarized in Table 1. The DSC curves are shown in Figures S1–S8 (Supporting Information).

**Table 1:** Phase transition temperatures (°C) and enthalpies Δ*H* (kJ mol^−1^) of cyclopropenium salts **7**, **8**, **9**, **10**, and reference ILCs **12**–**14** for comparison.^[a]^

Compound	Cr	*T* (Δ*H*)	Mesophase	*T* (Δ*H*)	I	Cycle	Ref.
**7 a**	*	98 (39.6)	–	–	*	2nd H	
**7 b**	*	103 (49.5) 82 (47.0)	SmA SmA	117 (0.8) 117 (1.0)	*	3rd H 3rd C	
**7 c**	*	106 (54.3) 84 (52.2)	SmA SmA	140 (0.9) 140 (0.9)	*	3rd H 3rd C	
**8 a**	*	113	–	–	*	POM	
**8 b**	*	114	–	–	*	POM	
**8 c**	*	114	–	–	*	POM	
**9**	*	55 (81.2)	Col_r_	63 (15.4)	*	1st H^[b]^	
**10 a**	*	47 (68.3) 5 (14.5)	Col_h_ Col_h_	169 (0.7) 169^[c]^	*	1st H 1st C	
**10 b**	* ^[d]^	121 (22.9)	–	–	*	1st H	
**10 c**	* ^[e]^	103 (1.6) 102 (2.0)	SmA SmA	112^[c]^ 112^[c]^	*	2nd H 2nd C	
**12 a**	*	93 (50.0)	SmA	123 (0.8)	*	2nd H	
**12 b**	*	91 (52.1)	SmA	149 (1.2)	*	2nd H	
**13 a**	*	51 (54.5)	Col_h_	146 (0.5)	*	2nd H	55b
**13 b**	*	33 (32.7)	Col_h_	228^[f]^	*	1st H	55b
**14**	*	31 (50.1)	Col_h_	139 (32.2)	*	2nd H

[a] Phases observed: crystalline (Cr), smectic A (SmA), columnar rectangular (Col_r_), columnar hexagonal (Col_h_), isotropic liquid (I). [b] Heating rate 5 K min^−1^. [c] Determined by POM, in the DSC no transition was observed. [d] Additional Cr–Cr transitions. [e] Cr–Cr transition at 54 °C (3.6 kJ mol^−1^). [f] Decomposition upon reaching isotropic melt. H denotes heating, C denotes cooling.

**Figure 3:**
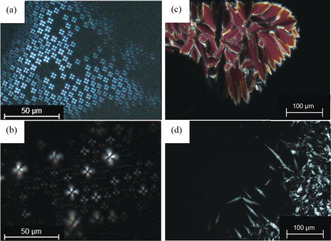
Textures of cyclopropenium derived ILCs as seen between crossed polarizers upon cooling from the isotropic liquid. a) Maltese cross textures of **7 b** at 119 °C and b) **7 c** at 144 °C (cooling rate 5 K min^−1^, magnification ×200; scale bar=50 μm). c) Mosaic texture of **10 a** at 169 °C and d) Bâtonnet texture of **10 c** at 107 °C (cooling rate 5 K min^−1^, magnification ×100; scale bar=100 μm).

Phenoxy bis(dimethylamino)cyclopropenium tetrafluoroborate **7 a** with a C_12_ side chain showed only isotropic melting at 98 °C upon heating. No evidence for additional phase transitions was detected during subsequent cooling/heating cycles. In contrast, **7 b** bearing a C_14_ side chain revealed endothermal melting at 103 °C and a clear transition at 117 °C upon heating. During subsequent cooling, an isotropic to mesophase transition at 117 °C and a crystallization at 82 °C was visible. Derivative **7 c** with a C_16_ side chain showed similar behavior with phase transitions at 106 °C and 140 °C upon heating, and 140 °C and 84 °C upon cooling, indicating some hysteresis because of supercooling. Under the POM, Maltese cross textures and a strong tendency for homeotropic alignment were observed (Figures 3 a,b), suggesting the presence of smectic A (SmA) mesophases.

**Figure 4:**
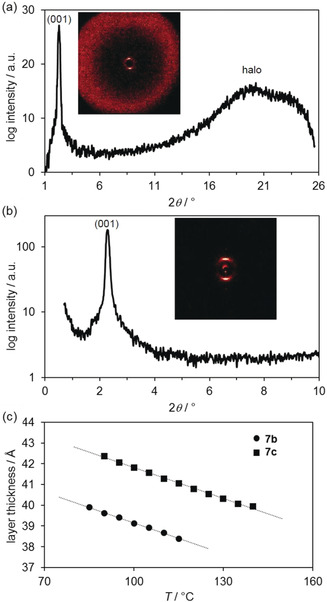
a) WAXS and b) SAXS profile of **7 b** at 110 °C. Inset: the respective diffraction image. c) Plot of layer distance versus temperature for cyclopropenium tetrafluoroborates **7 b** and **7 c**.

XRD experiments with **7 b** showed a distinct reflection in the small‐angle region at 2*θ*=2.2°/38.7 Å, which was assigned as the (001) layer reflex, and a broad halo around 2*θ*=15–26°, which is typical for the molten alkyl chains of the LC self‐assembly (Figures 4 a,b).

Temperature‐dependent XRD measurements provided the smectic layer distance of **7 b** and **7 c** as a function of temperature (Figure 4 c). The ratio of the experimentally determined smectic layer distance l_001_ of 39.6 Å (at 105 °C), with respect to the calculated molecular length of 27 Å for **7 b** using the Avogadro program,^61^ suggested a SmA bilayer‐type arrangement with considerable interdigitation.

Increasing the steric bulkiness of the aminocyclopropenium head group resulted in complete loss of mesomorphism, as observed for phenoxy bis(diisopropylamino)cyclopropenium tetrafluoroborates **8** (Table 1). These derivatives were non‐mesomorphic with melting points ranging from 113–114 °C. In the solid state a similar ionic sublayer was found for derivative **8 a**, as compared to the mesophase packing of **7 b** and **7 c** with less bulky head groups. Although direct correlations between solid‐state structures and mesophase structures have to be made with great care, crystallographic data might provide some useful insight into mesomorphic self‐assembly or rationale for the absence of mesomorphism.^62^, ^63^ Despite the hydrogen bond NH−F3 between the aminocyclopropenium N−H unit and the fluoro group of the counterion, which should promote liquid crystallinity, interdigitation of the alkyl chains, and thus, van der Waals interactions are less pronounced in compound **8 a**. Moreover, π–π stacking was absent in the solid‐state structure, in contrast with the corresponding mesogenic guanidinium salts.^64^ These counterbalanced effects, in particular the interdigitation present only in small compartments (Supporting Information, Figure S15), together with the steric bulkiness of the cationic headgroup, seem to disfavor mesomorphic self‐assembly.

For comparison, the mesomorphic properties of the guanidinium tetrafluoroborates **12 a** and **12 b** with the same chain lengths and counterion as the corresponding aminocyclopropenium tetrafluoroborates **7 a** and **7 b** were studied. POM investigations of the guanidinium derivatives showed Maltese crosses (Supporting Information, Figures S9d,e). XRD studies revealed a sharp (001) reflex and a broad halo (Supporting Information, Figures S13 and S14). The layer distance calculated from the (001) reflex decreased with increasing temperature. Together, this indicates a SmA phase for these derivatives.

Comparing the results for **7** and **12**, both are forming SmA phases, showing that the bis(dimethylamino)cyclopropenium cation behaved as an extended deltic guanidinium cation with a slightly decreased mesophase stability and temperature range. Notably, the smectic layer arrangement is favored despite the increase of the cross‐sectional area of the head group of **7**, as compared to the guanidinium derivatives **12**.

Subsequently, the mesomorphic properties of wedge‐shaped gallic acid phenyl‐ester‐based cyclopropenium salts **10** and the synthetic precursor **9** were investigated. Ammonium salt **9** presented a mesophase between 59 °C and 64 °C. Although under the POM textures (Supporting Information, Figure S9a,b) were visible upon repeated heating and cooling, DSC analysis revealed thermal decomposition.^65^ Therefore, the phase transition temperatures were taken from the first heating. XRD experiments showed several reflexes in the small‐angle region. These were assigned as (10), (11), (04), (40), (42), (50), and (52) reflexes, indicating a columnar rectangular (Col_r_) mesophase with *p*2*mm* symmetry (Supporting Information, Figure S10). Derivative **10 a** displayed an endothermal melting transition at 47 °C and an endothermal clearing transition at 169 °C upon heating, and an exothermal crystallization at 5 °C upon cooling. The isotropic to mesophase transition temperature of 169 °C was taken from POM experiments. Under the POM, mosaic textures were observed (Figure 3 c), suggesting either a planar aligned columnar phase or a lamellar phase.

The small‐angle X‐ray scattering (SAXS) profile of **10 a** displayed three sharp reflections with a ratio of 1:1/√3:1/√4, which were indexed as (10), (11) and (20) reflections of a hexagonal columnar lattice with *p6mm* symmetry, and a lattice parameter of 49.0 Å was calculated. In the wide‐angle region a broad halo around 4.5 Å was observed (Table 2; Supporting Information, Figure S11). Taking the value of 4.5 Å for the average chain distance into the calculation of the number of molecules per columnar repeat (*Z*), *Z=*5 was obtained.^66^ In contrast, the increased bulkiness of the 1,2‐bis(diisopropylamino)cyclopropenium headgroup in derivative **10 b** strongly disfavored mesomorphic self‐assembly.

**Table 2:** XRD data of phenylalkoxybenzoate based ILCs **7 b** and **7 c**, **9**, **10 a** and **10 c**, **12 a** and **12 c**, and **14**.

Compound	Mesophase	Lattice parameter [Å]	*d* Values [Å] expt (calcd)	Miller indices
**7 b**	SmA at 105 °C	–	39.61 4.4	(001) halo
**7 c**	SmA at 115 °C	–	42.40 4.4	(001) halo
**9**	Col_r_ at 60 °C *p*2*mm*	*a*=57.90 *b*=52.34	52.34 38.83 (38.83) 14.17 (14.47) 13.06 (13.09) 11.95 (11.92) 10.47 (10.47) 9.76 (9.85) 4.1	(01) (11) (41) (04) (24) (05) (25) halo
**10 a**	Col_h_ at 160 °C *p*6*mm*	*a*=49.00	42.44 24.25 (24.50) 21.12 (21.22) 4.5	(10) (11) (20) halo
**10 c**	SmA at 115 °C	–	49.92 24.79 (24.96) 4.2	(001) (002) halo
**12 a**	SmA at 95 °C	–	36.61 4.5	(001) halo
**12 b**	SmA at 95 °C	–	39.58 4.7	(001) halo
**14**	Col_h_ at 31 °C *p*6*mm*	*a*=52.43	45.41 22.75 (22.70) 4.5	(10) (20) halo

**Figure 5:**
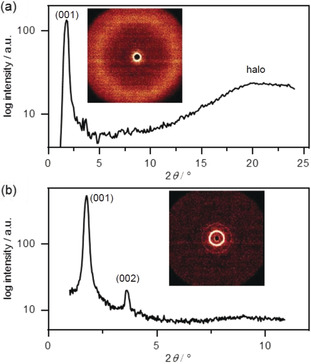
a) WAXS and b) SAXS profile of **10 c** at 171 °C (a) and 115 °C (b). Inset: the respective diffraction image.

When cyclopropenium chloride **10 c** was heated in the DSC, an endothermal Cr–Cr transition at 54 °C and an endothermal melting transition at 103 °C was measured. No clearing transition could be detected until decomposition occurred, but POM revealed a clearing temperature of 112 °C. In the cooling cycle the corresponding phase transitions were observed at 112 °C (POM) and 102 °C (DSC), indicating enantiotropic behavior. Bâtonnet textures with homeotropic domains under the POM (Figure 3 d) suggested the presence of a SmA mesophase. In the small‐angle regime a sharp reflection at 2*θ*=1.8°/49.9 Å and a reflex at 2*θ*=3.6°/24.8 Å were observed that were assigned as (001) and (002) smectic layer reflexes (Figure 5 b). The wide‐angle X‐ray scattering (WAXS) profile showed a broad halo around 4.2 Å (Figure 5 a). As a consequence of increasing decomposition of the sample with prolonged exposure time, temperature‐dependent layer spacings could not be determined. Presumably, the dimethoxybenzyl (DMB) protecting groups were cleaved under these conditions.^67^

Structurally related guanidinium derivatives were considered for comparison. Compounds **13 a** and **13 b** are already known^27^ and demonstrated a columnar hexagonal (Col_h_) mesophase. For derivative **13 a** with triflate anion, an enantiotropic mesophase was observed between 51 °C and 146 °C. Derivative **13 b**, with tetrafluoroborate anion, had a broader mesophase range because of a lower melting point at 33 °C and a much higher clearing temperature at 228 °C, leading to decomposition upon clearing. Derivative **14**, with a trimethyl ammonium head group, was also investigated. DSC analysis revealed a mesophase between 31 °C and 139 °C. XRD studies, together with POM textures (Supporting Information, Figures S9c and S12) revealed the presence of a Col_h_ mesophase.

Initially, we surmised that the bulky 2,4‐dimethoxybenzyl protecting group might deteriorate any liquid crystalline self‐assembly, in particular considering the size misfit of headgroup and core unit. However, the results demonstrate that nanosegregation is favored because of polyphilic interactions. Presumably, a smectic bilayer‐type organization is realized by a π–π stacked electron‐rich aryl layer, followed by a charged layer, where the cyclopropenium cations are counterbalanced by the tetrafluoroborate anions, followed by an aryl layer of the gallic acid phenyl esters and a hydrophobic layer. Notably, derivative **11** carrying two cyclopropenium headgroups was non‐mesomorphic. Thus, the combination of steric hindrance and Coulomb repulsion of two cations in a close vicinity seems to disfavor liquid crystallinity.

## Conclusion

**Figure 6:**
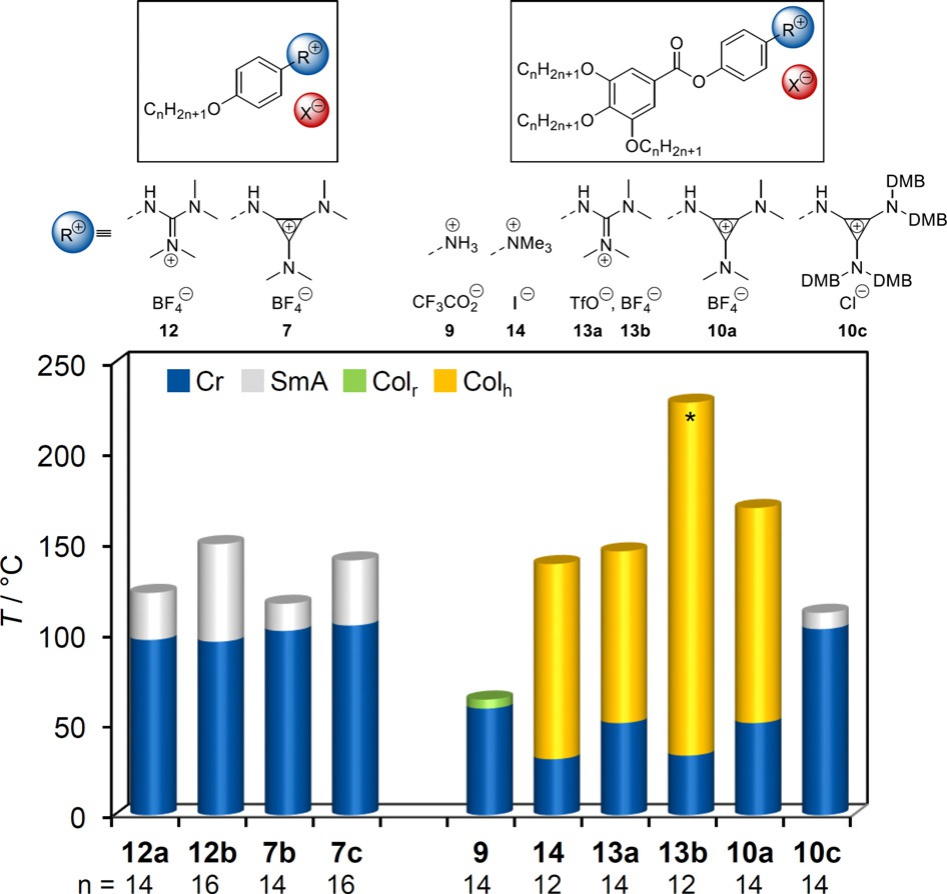
Comparison of mesophases of alkoxyphenyl and phenylalkoxybenzoate‐based ILCs depending on the headgroup ammonium (**9**, **14**), guanidinium (**12**, **13**), and aminocyclopropenium (**7**, **10**; * denotes decomposition). The values of **13 a** and **13 b** were taken from ref. 27.

**Figure 7:**
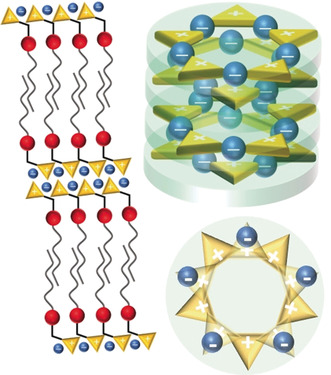
Proposed packing models of the observed mesophases. Smectic A_d_ phase with double layers (left) for derivatives with one alkyl chain or the huge steric demand of the cyclopropenium head group. Columnar mesophase from side (top right) and top view (bottom right) for derivatives with three alkyl chains and the reduced steric demand of the cyclopropenium head group.

We demonstrate, for the first time, that aminocyclopropenium salts self‐assemble into liquid crystalline mesophases. Nanosegregation of immiscible parts, electrostatic interactions, and volume requirements of both the head group and hydrophobic parts play a major role in controlling the mesophase stability. The geometry of the mesophase was determined by the effective volume of headgroup versus hydrophobic part, in agreement with Israelachvili's packing model for lyotropic liquid crystals.^68^ Thus, aminocyclopropenium salts with small *N*,*N*‐dimethylamino substituents and a single alkoxy chain attached to the aryl unit (**7 b** and **7 c**) form lamellar geometries (Figures 6 and 7), which is similar to the corresponding guanidinium salts **12 a** and **12 b**. With increasing steric demand of the headgroup mesomorphism is lost (for example, *N*,*N*‐diisopropylamino **8 a**–**c**). Precursor **9** with a polar ammonium headgroup was capable of forming a Col_r_ mesophase with *p2mm* symmetry. On the other hand, wedge‐shaped aminocyclopropenium salts with *N*,*N*‐dimethylamino groups self‐assemble into micellar‐like columnar geometries (for example, **10 a**; Figure 7), which is similar to trimethylammonium (**14**) and guanidinium salts **13 a** and **13 b**. Again, mesomorphism was lost with more sterically demanding headgroups (diisopropylamino). However, when the headgroup surpasses a certain size requiring a similar volume as the hydrophobic part and provides additional polyphilic interactions, lamellar mesophases were found again (for example, **10 c**). These results are in good agreement with molecular dynamics (MD) simulations on pyridinium ILCs, which revealed that only those compounds with a relatively large volume ratio of cation to anion form stable SmA phases.^69^

In conclusion, aminocyclopropenium ILCs serve as well‐defined model compounds to study self‐assembly and nanosegregation, which are important in polyelectrolytes used for battery materials. These ILCs bridge the gap between low molecular weight organocatalysts and polymeric electrolytes, and thus, contribute to the general utility of 3‐ring aromatic compounds.

## Conflict of interest

The authors declare no conflict of interest.

## Supporting information

As a service to our authors and readers, this journal provides supporting information supplied by the authors. Such materials are peer reviewed and may be re‐organized for online delivery, but are not copy‐edited or typeset. Technical support issues arising from supporting information (other than missing files) should be addressed to the authors.

Supplementary
