# Supplementary material for: Self‐Assembly of Aminocyclopropenium Salts: En Route to Deltic Ionic Liquid Crystals
Source: Angew Chem Int Ed Engl. 2020 Apr 7;59(26):10557–65. doi: 10.1002/anie.202000824 (PMC7317216; doi:10.1002/anie.202000824)
Supplement: Supplementary file 1 — Supplementary [file ANIE-59-10557-s001.pdf]

Angewandte Chemie
Eine Zeitschrift der Gesellschaft Deutscher Chemiker

Supporting Information

Self-Assembly of Aminocyclopropenium Salts: En Route to Deltic Ionic Liquid Crystals

Juri Litterscheidt, Jeffrey S. Bandar, Max Ebert, Robert Forschner, Korinna Bader, Tristan H. Lambert,* Wolfgang Frey, Andrea Bühlmeyer, Marcus Brändle, Finn Schulz, and Sabine Laschat**

anie_202000824_sm_miscellaneous_information.pdf

Author Contributions

The manuscript was written through contributions of all authors, who have given approval to the final version of the manuscript. J.L. designed, synthesized, and characterized all cyclopropenium compounds, and performed POM and DSC analysis, J.S.B. synthesized the cyclopropenium precursors, contributed his synthetic expertise on non-benzenoid compounds, and proofread the manuscript. M.E. synthesized and characterized guanidinium ILCs, and performed DSC and POM experiments and data analysis. R.F., K.B., A.B., and F.S. performed XRD experiments. T.H.L. supervised, provided his expertise on aminocyclopropenium salts, and proofread the manuscript. W. F. performed the X-ray single-crystal structure analyses. M.B. synthesized and characterized ammonium ILCs. S.L. coordinated the research, and wrote and proofread the manuscript.

Table of Contents

1) General Methods.. S2

2) Experimental Procedures.. S2

3) References... S14

4) Differential Scanning Calorimetry (DSC) Curves.. S15

5) Polarizing Optical Microscopy (POM) of Derivatives **9**, **12** and **14**............................... S19

6) X-Ray Diffraction (XRD) Measurement of Derivatives **9**, **10a**, **12** and **14**.................... S20

7) X-Ray Single-crystal Structure Analysis... S25

8) NMR Data of New Compounds.. S27

1) General Methods

NMR spectra were recorded on 400, 500 or 700 MHz spectrometers at room temperature. Infrared spectra were recorded on a Fourier transform infrared (FT-IR) spectrometer with Platinum ATR system at room temperature. Mass spectra (MS) and high-resolution mass spectra (HRMS) were recorded using the ESI-TOF technique. For POM analysis an *Olympus* BX50 microscope (heat unit: *Linkam* BX50) was used. For DSC measurements, the samples were placed in *Mettler Toledo* aluminum pans (40 µL). Recording of the respective thermograms was performed on a *Mettler Toledo* DSC 822e calorimeter. X-ray diffraction in the mesophase was performed using a *Bruker* Nanostar C ($Cu_{K\alpha}$: $\lambda = 1.5406$ Å) with an equipped HI-STAR detector. The limit from the instrument is 2Theta ≈ 25°. Single crystal analysis was conducted on a *Bruker* kappa APEXII Duo diffractometer. Data collection: APEX2 Software Suite; cell refinement: SAINT (both Bruker 2008). The structures were solved by using the program SHELXS 97 (Sheldrick 2008) and refined by using the program SHELXL 97 (Sheldrick 2008). Molecular graphics: XP in SHELXTL-Plus (Sheldrick 2008). Thin-layer chromatography was performed on silica gel 60 F_{254} precoated aluminium plates. Column chromatography was carried out using silica gel (grain size of 40–63 µm) with solvents distilled prior to use. Commercially available reagents were used as purchased unless otherwise stated. Solvents were distilled prior to use or purified and dried by standard procedures. Reactions under Schlenk conditions were performed in dried glassware under inert gas atmosphere.

2) Experimental Procedures

A direct synthesis of 1,2-bis(dimethylamino)-3-chlorocyclopropenium chloride **3a** from commercially available pentachlorocyclopropane **1** and dimethylamine was not feasible due to the high reactivity of dimethylamine leading to the threefold-substituted cyclopropenium salt and ring-opening products.[1,3,4]

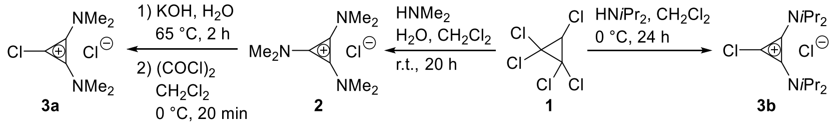

Scheme S1.

Tris(dimethylamino)cyclopropenium chloride (2).[1] To a solution of **1** (3.90 mL, ~85% purity, 6.51 g, 30.4 mmol) in CH_2Cl_2 (100 mL) at 0 °C was added a 40% aqueous solution of dimethylamine (25.0 mL, 220 mmol) over 15 min, and the reaction mixture was stirred for 2 h at 0 °C and for a further 18 h at room temperature. The layers were separated, and the aqueous

layer was extracted with CH_2Cl_2 (2 × 50 mL). The solvent was removed under vacuum and the residue was dissolved under heating in MeCN / toluene (2 : 1). The solution was slowly cooled to –10 °C and filtered after 12 h. After removal of all volatile materials, the filtrate was dissolved in boiling EtOAc and then cold brine was added. The mixture was slowly cooled to 0 °C at which point **2** (1.35 g, 6.61 mmol, 22%) crystallized as yellowish-brown needles. **^{1}H NMR** ($CDCl_3$, 700 MHz): δ = 3.09 (s, 18 H, C*H*$_3$) ppm; **^{13}C NMR** ($CDCl_3$, 176 MHz): δ = 42.3 (N*C*H$_3$), 118.0 (C-1) ppm; **FT-IR:** $\tilde{\nu}$ = 2938 (w), 2907 (w), 1641 (w), 1556 (m), 1455 (w), 1403 (w), 1282 (w), 1219 (w), 1133 (w), 1093 (w), 1047 (m), 786 (w), 520 (w) cm^{-1}; **MS** (ESI): *m/z* = 168.15 [M–Cl$^-$] ($C_9H_{18}N_3^+$). **HRMS** (ESI): *m/z* calcd for [M–Cl$^-$] 168.1495 ($C_9H_{18}N_3^+$), found: 168.1503.

The spectroscopic data were in agreement with those in the literature.[1]

3-Chloro-1,2-bis(dimethylamino)cyclopropenium chloride (3a). To a vigorously stirred solution of NaOH (0.50 g, 12.5 mmol) in H_2O (5 mL) **2** (1.35 g, 6.61 mmol) was added, and the reaction mixture was heated to 65 °C for 2 h and then cooled to room temperature. The pH value was adjusted to pH 3 with 1 M HCl, and the solution then poured into $CHCl_3$ (50 mL) / H_2O (100 mL). The layers were separated, all volatile materials removed under reduced pressure and the residue dried at 50 mbar and 35 °C. The residue (505 mg, 3.6 mmol) was dissolved under N_2 atmosphere in anhydrous CH_2Cl_2 (20 mL), oxalyl chloride (351 µL, 521 mg, 4.10 mmol) was added and the reaction mixture stirred for 20 min at room temperature. All volatile materials were removed under reduced pressure and the residue recrystallized from Et_2O / CH_2Cl_2 (2 : 1) to give **3a** (446 mg, 2.38 mmol, 36%) as brown crystals. **^{1}H NMR** ($CDCl_3$, 700 MHz): δ = 3.22 (s, 6 H, NC*H*$_3$), 3.29 (s, 6 H, NC*H*$_3$) ppm; **^{13}C NMR** ($CDCl_3$, 176 MHz): δ = 41.9, 42.9 (N*C*H$_3$), 91.4 (C-1), 134.5 (C-2) ppm; **FT-IR**: $\tilde{\nu}$ = 3388 (m), 2939 (w), 1945 (w), 1627 (m), 1524 (w), 1447 (w), 1410 (w), 1389 (w), 1274 (w), 1211 (w), 1146 (w), 1037 (w), 792 (w), 521 (w) cm^{-1}; MS (ESI): *m/z* = 159.07 [M–Cl$^-$] ($C_7H_{13}N_2Cl^+$); **HRMS** (ESI): *m/z* calcd. for [M–Cl$^-$] 159.0684 ($C_7H_{13}N_2Cl^+$), found: 159.0682.

The spectroscopic data were in agreement with those in the literature.[2]

3-Chloro-1,2-bis(diisopropylamino)cyclopropenium chloride (3b). To a solution of di-isoproplylamine (31.2 mL, 222 mmol, 22.5 g) in THF (40 mL) / CH_2Cl_2 (50 mL) was added dropwise a solution of **1** (4.76 g, 21.7 mmol) in CH_2Cl_2 (20 mL) over 50 min at 0 °C, and the reaction mixture was stirred for 24 h at room temperature. All volatile materials were re-moved under reduced pressure and the residue was dissolved in boiling acetone (40 mL) / toluene (20 mL). The mixture was cooled to room temperature with vigorous

stirring and then stored for 20 h at 7 °C. The precipitate was filtered off and the filtrate concentrated under reduced pressure. The residue was recrystallized from EtOAc to give **3b** (6.29 g, 20.4 mmol, 94%) as a colorless solid. Mp 122 °C. **^{1}H NMR** ($CDCl_3$, 400 MHz): δ = 1.22-1.35 (m, 24 H, 3-C*H*$_3$), 3.11-3.48 (m, 2 H, 3-H), 4.04-4.32 (m, 2 H, 3-H) ppm; **^{13}C NMR** ($CDCl_3$, 101 MHz): δ = 21.3, 22.7 (*C*H$_3$), 58.2 (C-3), 93.2 (C-1), 132.2 (C-2) ppm; **FT-IR**: $\tilde{\nu}$ = 3413 (s), 2979 (m), 2937 (w), 2824 (m), 2698 (s), 2569 (w), 2467 (m), 2179 (s), 1913 (m), 1582 (m), 1527 (w), 1459 (w), 1418 (w), 1375 (w), 1347 (w), 1206 (w), 1137 (m), 1020 (w), 925 (m), 723 (s), 639 (m), 571 (w), 508 (w) cm^{-1}; **MS** (ESI): *m/z* = 271.19 [M–Cl$^-$] ($C_{15}H_{28}N_2Cl^+$); **HRMS** (ESI): *m/z* calcd. for [M–Cl$^-$] 271.1936 ($C_{15}H_{28}N_2Cl^+$), found: 271.1936.

3-Chloro-1,2-bis(bis(2,4-dimethoxybenzyl)amino)cyclopropenium chloride (3c).[3] To a stirred mixture of tetrachlorocyclopropene **4** (2.87 g, 1.92 mL, 15.6 mmol) and K_2CO_3 (4.57 g, 33.0 mmol) in CH_2Cl_2 (100 mL) / H_2O (30 mL) at 0 °C under Ar atmosphere was added dropwise a solution of bis(2,4-dimethoxybenzyl)amine (9.12 g, 28.9 mmol) in CH_2Cl_2 (300 mL) over 2.5 h. The reaction mixture was warmed to room temperature and stirred for 50 min. The layers were separated, and the organic layer was washed with brine (100 mL), dried (Na_2SO_4), filtered and concentrated under vacuum to give the crude product as an orange oil. The oil was washed with Et_2O / CH_2Cl_2 (2 : 1) to give **3c** as a light yellow precipitate (7.27 g, 9.83 mmol, 63%). Derivative **5** was precipitated from the solution by slow addition of Et_2O / hexanes (2 : 1) (1.91 g, 1.87 mmol, 12%).
The spectroscopic data were in agreement with those in the literature.[3]

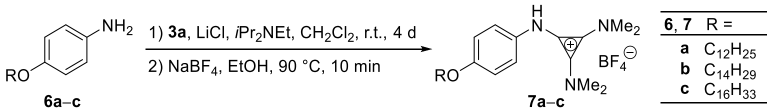

Scheme S2

Synthesis of 1,2-bis (dimethylamino)cyclopropenium tetrafluoroborates 7; General Procedure GP1.

To a solution of **6** (1.50 mmol), anhydrous LiCl (2.00 mmol) and Hünig's base (323 mg, 2.50 mmol) in CH_2Cl_2 (80 mL) was added **3a** (0.80 mmol), and the reaction mixture was stirred for 4 d at room temperature. The solvent was removed under reduced pressure and the residue chromatographed on SiO_2 with CH_2Cl_2 / EtOH (20 : 1→15 : 1→8 : 1). The crude product was taken up in CH_2Cl_2 / EtOH (5 : 1), $NaBF_4$ (329 mg, 3.00 mmol) was added and the reaction mixture heated for 10 min at 80 °C. After removal of the solvent under reduced

pressure, the residue was taken up in CH_2Cl_2 and filtered. The solvent was removed, and the product obtained as light brown solid.

***N*^{3}-(4-Dodecyloxyphenyl)-*N*^{1},*N*^{1},*N*^{2},*N*^{2}-tetramethyl-1,2,3-triaminocyclopropenium tetrafluoroborate (7a).** According to GP1, from **6a** (834 mg, 3.00 mmol), Hünig's base (2.25 mL, 1.71 g, 13.1 mmol), LiCl (55.0 mg, 1.31 mmol) and **3a** (302 mg, 1.54 mmol); yield: 279 mg, 0.57 mmol, 37%. Mp 98 °C. **^{1}H NMR** ($CDCl_3$, 400 MHz): δ = 0.88 (t, *J* = 7.0 Hz, 3 H, CH_2-CH_3), 1.20–1.39 (m, 16 H, CH_2), 1.39–1.49 (m, 2H, CH_2-CH_2-CH_2O), 1.70–1.81 (m, 2 H, CH_2-CH_2O), 3.07 (s, 12 H, NCH_3), 3.90 (t, *J* = 6.5 Hz, 2 H, CH_2O), 6.83 (m, 2 H, *o*-H), 7.16 (m, 2 H, *m*-H), 8.58 (s, 1 H, N*H*) ppm; **^{13}C NMR** ($CDCl_3$, 101 MHz): δ = 14.1 (CH_2-*C*H_3), 22.7, 26.0, 29.3, 29.4, 29.6, 29.7, 31.9 (*C*H_2), 42.1 (N*C*H_3), 68.4 (*C*H_2O), 112.9 (C-2), 115.3 (C-*o*), 118.0 (C-1), 123.0 (C-*m*), 132.0 (C-*p*), 157.3 (C-*i*) ppm; FT-IR: $\tilde{\nu}$ = 3321 (s), 2923 (m), 2853 (w), 1582 (w), 1541 (w), 1515 (w), 1468 (w), 1428 (w), 1243 (w), 1056 (s), 831 (w), 521 (w) cm^{-1}; MS (ESI): *m/z* = 400.33 [M–BF_4^-] ($C_{25}H_{42}N_3O^+$). **HRMS** (ESI): *m/z* calcd. for [M–BF_4^-] 400.3322 ($C_{25}H_{42}N_3O^+$), found: 400.3275; Anal. calcd. for $C_{25}H_{44}BF_4N_3O$ (489.45): C 61.60, H 8.69, N 8.62; found: C 61.77, H 8.57, N 8.47.

***N*^{3}-(4-Tetradecyloxyphenyl)-*N*^{1},*N*^{1},*N*^{2},*N*^{2}-tetramethyl-1,2,3-triaminocyclopropenium tetrafluoroborate (7b).** According to GP1, from **6b** (917 mg, 3.00 mmol), Hünig's base (2.02 mL, 1.54 g, 11.8 mmol), LiCl (61.0 mg, 1.45 mmol) and **3a** (298 mg, 1.52 mmol), yield: 320 mg 0.62 mmol, 41%. **^{1}H NMR** ($CDCl_3$, 400 MHz): δ = 0.88 (t, *J* = 7.0 Hz, 3 H, CH_2-CH_3), 1.23–1.38 (m, 20 H , CH_2), 1.39–1.43 (m, 2H, CH_2-CH_2-CH_2O), 1.70–1.80 (m, 2 H, CH_2-CH_2O), 3.06 (s, 12 H, NCH_3), 3.89 (t, *J* = 6.7 Hz, 2 H, CH_2O), 6.83 (m, 2 H, *o*-H), 7.16 (m, 2 H, *m*-H), 8.56 (s, 1 H, N*H*) ppm; **^{13}C NMR** ($CDCl_3$, 101 MHz): δ = 14.1 (CH_2-*C*H_3), 22.7, 26.1, 29.3, 29.4, 29.6, 29.7, 31.9 (*C*H_2), 42.0 (N*C*H_3), 68.4 (*C*H_2O), 112.7 (C-2), 115.3 (C-*o*), 118.1 (C-1), 122.9 (C-*m*), 132.0 (C-*p*), 157.2 (C-*i*) ppm; **FT-IR**: $\tilde{\nu}$ = 3322 (s), 2919 (m), 2851 (w), 1580 (w), 1538 (w), 1508 (w), 1468 (w), 1427 (w), 1239 (m), 1175 (w), 1051 (s), 831 (w), 757 (w), 521 (w) cm^{-1}; **MS** (ESI): *m/z* = 428.36 [M–BF_4^-] ($C_{27}H_{46}N_3O^+$); **HRMS** (ESI): *m/z* calcd. for [M–BF_4^-] 428.3635 ($C_{27}H_{46}N_3O^+$), found: 428.3635; Anal. calcd. for $C_{27}H_{46}BF_4N_3O$ (515.49): C 62.91, H 8.99, N 8.15; found: C 62.96, H 9.03, N 8.19. DSC: Cr 103 °C [49.5 kJ/mol] SmA 117 °C [0.8 kJ/mol] I.

***N*^{3}-(4-Hexadecyloxyphenyl)-*N*^{1},*N*^{1},*N*^{2},*N*^{2}-tetramethyl-1,2,3-triaminocyclopropenium tetrafluoroborate (7c).** According to GP1, from **6c** (126 mg, 0.32 mmol), Hünig's base (2.06 mL, 1.56 g, 11.9 mmol), LiCl (125 mg, 2.98 mmol), **3a** (68.7 mg, 0.35 mmol), yield: 53.2 mg 99.2 µmol, 32%. **^{1}H NMR** ($CDCl_3$, 400 MHz): δ = 0.88 (t, *J* = 7.0 Hz, 3 H, CH_2-

CH_3), 1.19–1.48 (m, 26 H, CH_2), 1.70–1.80 (m, 2 H, CH_2-CH_2O), 3.07 (s, 12 H, NCH_3), 3.90 (t, *J* = 6.6 Hz, 2 H, CH_2O), 6.83 (m, 2 H, *o*-H), 7.16 (m, 2 H, *m*-H), 8.58 (s, 1 H, N*H*) ppm; **^{13}C NMR** ($CDCl_3$, 101 MHz): δ = 14.1 (CH_2-CH_3), 22.7, 26.1, 29.3, 29.4, 29.6, 29.7, 31.9 (CH_2), 42.1 (NCH_3), 68.3 (CH_2O), 112.8 (C-2), 115.3 (C-*o*), 118.1 (C-1), 123.0 (C-*m*), 132.0 (C-*p*), 157.2 (C-*i*) ppm; **FT-IR**: $\tilde{\nu}$ = 3309 (s), 2917 (m), 2850 (w), 1587 (w), 1536 (w), 1515 (w), 1475 (w), 1426 (w), 1240 (w), 1050 (s), 916 (w), 833 (w), 735 (w), 520 (w) cm^{-1}; **MS** (ESI): *m/z* = 456.40 [M–BF_4^-] ($C_{29}H_{50}N_3O^+$); **HRMS** (ESI): *m/z* calcd. for [M–BF_4^-] 456.3954 ($C_{29}H_{50}N_3O^+$), found: 456.3960; Anal. calcd. for $C_{29}H_{50}BF_4N_3O$ (543.54): C 64.08, H 9.27, N 7.73; found: C 63.77, H 8.95, N 7.04.

DSC: Cr 106 °C [53.8 kJ/mol] SmA 140 °C [0.8 kJ/mol] I.

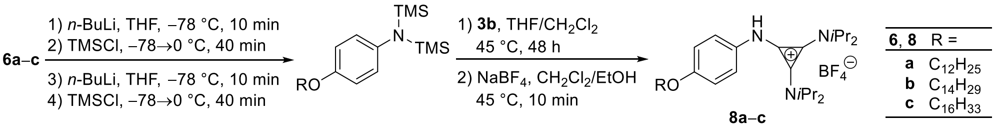

Scheme S3

Synthesis of 1,2-bis(diisopropylamino)cyclopropenium tetrafluoroborates 8; General Procedure GP2.

To a solution of **6** (0.50 mmol) in dry THF (40 mL) at –60 °C under inert atmosphere was added a 2.5 M solution of *n*-BuLi in hexanes (0.2 ml, 0.50 mmol), and the reaction mixture was stirred for 10 min. After addition of TMS-Cl (59.8 mg, 0.55 mmol), the reaction mixture was warmed to room temperature over 40 min. Then the mixture was recooled to –60 °C and a 2.5 M solution of *n*-BuLi in hexane (0.2 ml, 0.50 mmol) was added followed by addition of TMS-Cl (59.8 mg, 0.55 mmol) after 10 min. The reaction mixture was warmed to room temperature over 40 min and stirred for a further 30 min. Then **3b** (0.60 mmol) and dry CH_2Cl_2 (20 mL) were added, and the reaction mixture was stirred at 25 °C for 2 h and at 45 °C for 42 h. All volatile materials were removed under reduced pressure and the residue taken up in $CHCl_3$ (40 mL) and filtered. To the filtrate 20 mL of a solution of HBF_4 (3.51 g, 20.0 mmol, 48wt% in H_2O) was added and the emulsion stirred for 30 min. The layers were separated, and the aqueous layer extracted with $CHCl_3$ (2 × 40 mL). The combined organic layers were concentrated under reduced pressure and the residue purified by column chromatography on SiO_2 (CH_2Cl_2 / EtOH 10 : 0→10 : 1→5 : 1). For the ion exchange the crude product was taken up in CH_2Cl_2 / EtOH (5 : 1) and filtered through a pad of HBF_4 treated SiO_2 (2 cm). SiO_2 was washed with CH_2Cl_2 / EtOH (100 mL / 30 mL) and the filtrate concentrated under reduced pressure. The residue was taken up in CH_2Cl_2 (100 mL) and filtered

to remove traces of SiO_2. The filtrate was concentrated, and the residue recrystallized from EtOAc *in vacuo* and dried in a vacuum desiccator over P_2O_5.

***N*³-(4-Dodecyloxyphenyl)-*N*¹,*N*¹,*N*²,*N*²-tetrakis(isopropyl)-1,2,3-triaminocyclopropenium tetrafluoroborate (8a)**. According to GP2, from **6a** (21.6 mg, 77.8 µmol), *n*-BuLi (0.06 mL, 158 µmol), TMS-Cl (0.05 mL, 45.1 mg, 0.40 mmol) and **3b** (8.90 mg, 28.9 µmol), yield: 15.6 mg, 26.0 µmol, 90%. Mp 113 °C. **^{1}H NMR** ($CDCl_3$, 500 MHz): δ = 0.89 (t, *J* = 7.3 Hz, 3 H, CH_2-C*H*$_3$), 1.13–1.37 (m, 40 H, C*H*$_2$, 3-C*H*$_3$), 1.37–1.47 (m, 2 H, C*H*$_2$-CH_2-CH_2O), 1.71–1.79 (m, 2 H, C*H*$_2$-CH_2O), 3.62–3.77 (m, 4 H, 3-H), 3.91 (t, *J* = 6.4 Hz, 2 H, C*H*$_2$O), 6.86 (m, 2 H, *m*-H), 7.21 (m, 2 H, *o*-H), 8.04 (s, 1 H, N*H*) ppm; **^{13}C NMR** ($CDCl_3$, 126 MHz): δ = 14.1 (CH_2-*C*H_3), 22.0 (C-4), 22.7, 26.0, 29.2, 29.4, 29.6, 29.7, 31.9 (*C*H_2), 50.9 (C-3), 68.4 (*C*H_2O), 114.1 (C-1), 115.2 (C-*m*), 115.4 (C-2), 125.8 (C-*o*), 131.5 (C-*p*), 157.8 (C-*i*) ppm; **FT-IR**: $\tilde{\nu}$ = 3304 (s), 2924 (s), 2853 (w), 1508 (m), 1467 (w), 1453 (w), 1393 (w), 1375 (w), 1349 (w), 1289 (w), 1237 (m), 1212 (w), 1191 (w), 1160 (w), 1138 (w), 1053 (w), 829 (w), 722 (w), 679 (w), 556 (w), 519 (w) cm^{-1}; **MS** (ESI): *m/z* = 512.46 [M – BF_4^-] ($C_{33}H_{58}N_3O^+$); **HRMS** (ESI): *m/z* calcd. for [M – BF_4^-] 512.4574 ($C_{33}H_{58}N_3O^+$), found: 512.4600; Anal. calcd. for $C_{33}H_{58}BF_4N_3O$ (599.65): C 66.10, H 9.75, N 7.01; found: C 66.18, H 9.74, N 6.91.

***N*³-(4-Tetradecyloxyphenyl)-*N*¹,*N*¹,*N*²,*N*²-tetrakis(isopropyl)-1,2,3-triaminocyclopropenium tetrafluoroborate (8b)**. According to GP2, from **6b** (32.1 mg, 105 µmol), *n*-BuLi (90.0 µL, 223 µmol), TMS-Cl (0.05 mL, 45.1 mg, 0.40 mmol) and **3b** (32.6 mg, 106 µmol), yield: 61.5 mg, 98.0 µmol, 93%. Mp 114 °C. **^{1}H NMR** ($CDCl_3$, 500 MHz): δ = 0.88 (t, *J* = 7.6 Hz, 3 H, CH_2-C*H*$_3$), 1.19–1.37 (m, 44 H, C*H*$_2$, 3-C*H*$_3$), 1.37–1.46 (m, 2 H, C*H*$_2$-CH_2-CH_2O), 1.72–1.78 (m, 2 H, C*H*$_2$-CH_2O), 3.62–3.77 (m, 4 H, 3-H), 3.91 (t, *J* = 6.4 Hz, 2 H, C*H*$_2$O), 6.86 (m, 2 H, *m*-H), 7.21 (m, 2 H, *o*-H), 8.04 (s, 1 H, N*H*) ppm; **^{13}C NMR** ($CDCl_3$, 126 MHz): δ = 14.1 (CH_2-*C*H_3), 22.0 (C-4), 22.7, 26.0, 29.2, 29.4, 29.6, 29.7, 31.9 (*C*H_2), 50.9 (C-3), 68.4 (*C*H_2O), 114.1 (C-1), 115.2 (C-*m*), 115.4 (C-2), 125.8 (C-*o*), 131.5 (C-*p*), 157.8 (C-*i*) ppm, **FT-IR**: $\tilde{\nu}$ = 3303 (s), 2922 (s), 2853 (w), 1508 (m), 1469 (w), 1452 (w), 1393 (w), 1375 (w), 1349 (w), 1289 (w), 1237 (m), 1212 (w), 1191 (w), 1160 (w), 1138 (w), 1053 (w), 884 (w), 828 (w), 722 (w), 679 (w), 556 (w), 519 (w) cm^{-1}; **MS** (ESI): *m/z* = 540.49 [M–BF_4^-] ($C_{35}H_{62}N_3O^+$); **HRMS** (ESI): *m/z* calcd. for [M–BF_4^-] 540.4887 ($C_{35}H_{62}N_3O^+$), found: 540.4887; Anal. calcd. for $C_{35}H_{62}BF_4N_3O$ (627.70): C 66.97, H 9.96, N 6.69; found: C 67.06, H 9.67, N 6.57.

***N*³-(4-Hexadecyloxyphenyl)-*N*¹,*N*¹,*N*²,*N*²-tetrakis(isopropyl)-1,2,3-triaminocyclopropenium tetrafluoroborate (8c)**. According to GP2, from **6c** (24.0 mg, 71.9 µmol), *n*-BuLi

(0.06 mL, 147 µmol), TMS-Cl (0.05 mL, 45.1 mg, 0.40 mmol) and **3b** (6.51 mg, 21.2 µmol), yield: 11.8 mg, 18.0 µmol, 85%. Mp 114 °C. **^{1}H NMR** (CDCl$_3$, 500 MHz): δ = 0.88 (t, *J* = 7.0 Hz, 3 H, CH$_2$-C*H*$_3$), 1.19–1.37 (m, 48 H, C*H*$_2$, 3-C*H*$_3$), 1.37–1.46 (m, 2 H, C*H*$_2$-CH$_2$-CH$_2$O), 1.72–1.90 (m, 2 H, C*H*$_2$-CH$_2$O), 3.62–3.77 (m, 4 H, 3-H), 3.91 (t, *J* = 6.4 Hz, 2 H, C*H*$_2$O), 6.86 (m, 2 H, *m*-H), 7.21 (m, 2 H, *o*-H), 8.04 (s, 1 H, N*H*) ppm; **^{13}C NMR** (CDCl$_3$, 126 MHz): δ = 14.1 (CH$_2$-*C*H$_3$), 22.0 (C-4), 22.7, 26.0, 29.2, 29.4, 29.6, 29.7, 31.9 (*C*H$_2$), 50.9 (C-3), 68.4 (*C*H$_2$O), 114.1 (C-1), 115.2 (C-*m*), 115.4 (C-2), 125.8 (C-*o*), 131.5 (C-*p*), 157.8 (C-*i*) ppm; **FT-IR**: $\tilde{\nu}$ = 3304 (s), 2922 (s), 2853 (w), 1508 (m), 1468 (w), 1452 (w), 1393 (w), 1375 (w), 1349 (w), 1289 (w), 1237 (m), 1212 (w), 1191 (w), 1160 (w), 1138 (w), 1053 (w), 915 (w), 828 (w), 727 (w), 679 (w), 556 (w), 519 (w) cm^{-1}; MS (ESI): *m/z* = 568.52 [M–BF$_4^-$] ($C_{37}H_{66}N_3O^+$); **HRMS** (ESI): *m/z* calcd. for [M–BF$_4^-$] 568.5200 ($C_{37}H_{66}N_3O^+$), found: 568.5206; Anal. calcd. for $C_{37}H_{66}BF_4N_3O$ (655.76): C 67.77, H 10.15, N 6.41; found: C 67.80, H 10.22, N 6.26.

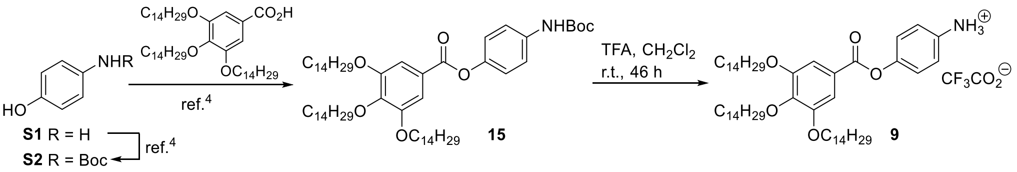

Scheme S4

4-[(*tert*-Butoxycarbonyl)amino]phenyl 3,4,5-tris(tetradecyloxy)benzoate (15). To a solution of **S2** (1.00 g, 4.78 mmol) in dry CH$_2$Cl$_2$ (500 mL) under inert atmosphere were added *N*,*N*-dimethylaminopyridine (DMAP) (20.0 mg, 0.16 mmol), dicyclohexylcarbodiimide (DCC) (1.03 g, 5.01 mmol) and 3,4,5-tris(tetradecyloxy)benzoic acid (3.55 g, 4.68 mmol). The reaction mixture was stirred at room temperature for 3 d prior to addition of H_2O (20 mmol). The reaction mixture was heated to reflux and filtered through a pad of SiO_2 (1 cm), which was washed with boiling CH$_2$Cl$_2$ (300 mL). The combined organic layers were concentrated under reduced pressure and the residue was recrystallized from EtOH to give **15** (3.38 g, 3.56 mmol, 76%) as colorless crystals. **^{1}H NMR** (CDCl$_3$, 500 MHz): δ = 0.84–0.91 (m, 9 H, CH$_2$-C*H*$_3$), 1.21–1.40 (m, 60 H, C*H*$_2$), 1.44–1.53 (m, 6 H, C*H*$_2$-CH$_2$-CH$_2$O), 1.53 (s, 9 H, 7-C*H*$_3$), 1.71–1.87 (m, 6 H, C*H*$_2$-CH$_2$O), 3.99–4.07 (m, 6 H, C*H*$_2$O), 6.62 (s, 1 H, N*H*), 7.11 (m, *o*-H), 7.35–7.46 (m, 4 H, *m*-H, 2-H) ppm; **^{13}C NMR** (CDCl$_3$, 126 MHz): δ = 14.1 (CH$_2$-*C*H$_3$), 22.7, 26.1, 29.3, 29.4, 29.6, 29.7, 29.8, 30.5, 31.8, 32.0 (CH$_2$), 28.3 (7-*C*H$_3$), 69.2 (3-O*C*H$_2$), 73.5 (4-O*C*H$_2$), 80.6 (C-7), 108.5 (C-2), 119.4 (C-*o*), 122.2 (C-*m*), 123.9 (C-1), 136.1, 142.9 (C-*i*, C-*p*), 146.3 (C-4), 152.8 (C-6), 153.0 (C-3), 165.2 (C-5) ppm.
The spectroscopic data were in agreement with those in the literature.[4]

4-{[3,4,5-Tris(tetradecyloxy)benzoyl]oxy}benzene ammonium trifluoroacetate (9). A suspension of trifluoroacetic acid (TFA) (4.00 mL, 5.92 g, 51.9 mmol) and **15** (2.10 g, 2.21 mmol) in CH_2Cl_2 (100 mL) was stirred at room temperature for 48 h. After concentration to 20 mL, the mixture was successively co-evaporated with toluene (2 × 50 mL) and acetone (3 × 100 mL). The crude product was recrystallized from EtOH and dried under vacuum to give **9** (2.00 g, 2.08 mmol, 94%) as a colorless solid. **^{1}H NMR** ($CDCl_3$, 400 MHz): δ = 0.85–0.91 (m, 9 H, CH_3), 1.21–1.41 (m, 60 H, -CH_2-), 1.42–1.53 (m, 6 H, CH_2-CH_2-CH_2O), 1.71–1.87 (m, 6 H, CH_2-CH_2O), 3.99–4.09 (m, 6 H, CH_2O), 7.01–7.25 (m, 7 H, *o*-H, *m*-H, NH_3), 7.37 (s, 2 H, 2-H) ppm; **^{13}C NMR** ($CDCl_3$, 101 MHz): δ = 14.1 (CH_3), 22.7, 26.1, 29.3, 29.4, 29.6, 29.7, 29.8, 31.9 (-CH_2-), 69.3 (3-OCH_2), 73.6 (4-OCH_2), 108.6 (C-2), 120.3 (C-*o*), 123.0 (C-*m*), 123.6 (C-1), 143.2, 147.5 (C-*i*, C-*p*, C-4), 153.0 (C-3), 165.3 (C-5) ppm; **FT-IR**: $\tilde{\nu}$ = 2916 (m), 2850 (w), 1731 (w), 1677 (w), 1587 (w), 1510 (w), 1467 (w), 1430 (w), 1388 (w), 1338 (w), 1191 (m), 1120 (w), 956 (w), 859 (w), 748 (w), 723 (w) cm^{-1}; **MS** (ESI): *m/z* = 850.73 [M–$C_2O_2F_3^-$] ($C_{55}H_{96}NO_5^+$). **HRMS** (ESI): *m/z* calcd. for [M–$C_2O_2F_3^-$] 850.7283 ($C_{49}H_{83}NO_5Na^+$), found: 850.7269; Anal. calcd. for $C_{55}H_{96}F_3NO_7$ (M) (964.39): C 70.99, H 10.03, N 1.45; Calcd. for (5 M–2 TFA): C 73.47, H 10.49, N 1.51; found: C 73.43, H 10.46, N 1.61.

DSC: Cr 55 °C [81.2 kJ mol^{-1}] Col$_h$ 63 °C [15.4 kJ mol^{-1}] I.

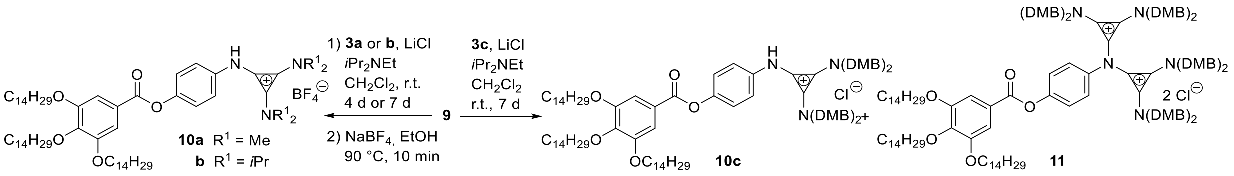

Scheme S5

Synthesis of 1,2-bis(dialkylamino)cyclopropenium salts 10; General Procedure GP3.

To a suspension of LiCl (2.00 mmol) and **9** (0.80 mmol) in CH_2Cl_2 (80 mL) were added Hünig's base (2.50 mmol) and the appropriate compound **3a**–**c** (1.20 mmol), and the reaction mixture was stirred at room temperature for 4–7 d. The mixture was then poured onto 0.5 M HCl (200 mL), the layers were separated, and the aqueous layer was extracted with CH_2Cl_2 (2 × 150 mL). The combined organic layers were washed with brine (200 mL), dried (Na_2SO_4) and concentrated under reduced pressure. The residue was purified by column chromatography on SiO_2 with CH_2Cl_2 / EtOH (15 : 1→5 : 1). For anion exchange the salt was dissolved in CH_2Cl_2 (20 mL). EtOH (5 mL) and $NaBF_4$ (5.00 mmol) were added, the suspension was heated at reflux for 10 min and then evaporated under reduced pressure. The residue

was taken up in high-purity $CHCl_3$ (30 mL), filtered and the solvent was removed under reduced pressure to give **10a**,**b**.

(4-{[3,4,5-Tris(tetradecyloxy)benzoyl]oxy}phenylamino)-2,3-bis(dimethylamino)cyclopropenium tetrafluoroborate (10a). According to GP3, from **9** (83.0 mg, 86.0 µmol), Hünig's base (3.32 mL, 2.52 g, 19.4 mmol), LiCl (112 mg, 2.67 mmol) and **3a** (201 mg, 1.03 mmol), yield: 12 mg, 12.0 µmol, 14%, light brown solid. **^{1}H NMR** ($CDCl_3$, 400 MHz): δ = 0.83–0.89 (m, 9 H, CH_2-CH_3), 1.20–1.39 (m, 60 H, CH_2), 1.42–1.52 (m, 6 H, CH_2-CH_2-CH_2O), 1.70–1.86 (m, 6 H, CH_2-CH_2O), 3.21 (s, 12 H, NCH_3), 3.97–4.08 (m, 6 H, CH_2O), 7.13 (m, *o*-H), 7.35 (s, 2 H, 2-H), 7.36 (m, *m*-H), 12.29 (s, 1 H, NH) ppm; **^{13}C NMR** ($CDCl_3$, 101 MHz): δ = 14.1 (CH_2-*C*H_3), 22.7, 26.0, 26.1, 29.3, 29.4, 29.6, 29.7, 31.9 (*C*H_2), 42.5 (N*C*H_3), 69.3 (3-O*C*H_2), 73.6 (4-O*C*H_2), 108.5 (C-2), 111.9 (C-7), 120.8 (C-*o*), 122.7 (C-*m*), 123.6 (C-6), 137.5, 143.1 (C-*i*, C-*p*), 147.7 (C-4), 153.0 (C-3), 165.0 (C-5) ppm; **FT-IR**: $\tilde{\nu}$ = 2919 (w), 2851 (w), 1732 (w), 1584 (w), 1545 (w), 1500 (w), 1467 (w), 1429 (w), 1337 (w), 1193 (w), 1118 (w), 722 (w) cm^{-1}; **MS** (ESI): *m/z* = 972.81 [M–BF_4^-] ($C_{62}H_{106}N_3O_5^+$); **HRMS** (ESI): *m/z* calcd. for [M–BF_4^-] 972.8127 ($C_{62}H_{106}N_3O_5^+$), found: 972.8125; Anal. calcd. for $C_{62}H_{106}BF_4N_3O_5$ (1060.34): C 70.23 H 10.08, N 3.96; found: C 70.39, H 10.15, N 3.79.
DSC: Cr 47 °C [65.1 kJ mol^{-1}] Col 170 °C I.

(4-{[3,4,5-Tris(tetradecyloxy)benzoyl]oxy}phenylamino)-2,3-bis(diisopropylamino)-cyclopropenium tetrafluoroborate (10b). According to GP3, from **9** (615 mg, 0.66 mmol), Hünig's base (3.36 mL, 2.54 g, 19.4 mmol), LiCl (110 mg, 2.66 mmol) and **3b** (223 mg, 0.77 mmol), yield: 116 mg, 10.3 µmol, 16%, light brown solid. Mp 121 °C. **^{1}H NMR** ($CDCl_3$, 400 MHz): δ = 0.82–0.86 (m, 9 H, CH_2-CH_3), 1.17–1.36 (m, 84 H, CH_2, 8-CH_3), 1.42–1.49 (m, 6 H, CH_2-CH_2-CH_2O), 1.70–1.83 (m, 6 H, CH_2-CH_2O), 3.71–3.81 (m, 4 H, 8-H), 3.98–4.04 (m, 6 H, CH_2O), 7.11 (m, *o*-H), 7.35 (s, 2 H, 2-H), 7.42 (m, *m*-H), 11.57 (s, 1 H, N*H*) ppm; **^{13}C NMR** ($CDCl_3$, 101 MHz): δ = 14.1 (CH_2-*C*H_3), 22.2 (8-*C*H_3), 22.7, 26.0, 26.1, 29.3, 29.4, 29.6, 29.7, 31.9 (-*C*H_2-), 51.2 (C-8), 69.3 (3-O*C*H_2), 73.3 (4-O*C*H_2), 108.5 (C-2), 114.3 (C-7), 122.4 (C-*o*), 123.7 (C-1), 123.9 (C-*m*), 138.1, 143.1 (C-*i*, C-*p*), 148.3 (C-4), 153.0 (C-3), 165.0 (C-5) ppm; **FT-IR**: $\tilde{\nu}$ = 2916 (w), 2849 (w), 1732 (w), 1690 (w), 1586 (w), 1509 (m), 1467 (w), 1336 (w), 1189 (w), 1161 (w), 1118 (w), 925 (w), 860 (w), 725 (m), 639 (w) cm^{-1}; **MS** (ESI): *m/z* = 1084.94 [M–BF_4^-] ($C_{70}H_{122}N_3O_5^+$); **HRMS** (ESI): *m/z* calcd. for [M–BF_4^-] 1084.9379 ($C_{70}H_{122}N_3O_5^+$), found: 1084.9385; Anal. calcd. for $C_{70}H_{122}BF_4N_3O_5$ (1172.56): C 71.70 H 10.49, N 3.58; found: C 71.70, H 10.63, N 3.46.

(4-{[3,4,5-Tris(tetradecyloxy)benzoyl]oxy}phenylamino)-2,3-tetrakis(2,4-dimethoxy-benzyl)cyclopropenium chloride (10c). According to GP3, from **9** (612 mg, 0.66 mmol), Hünig's base (3.32 mL, 2.52 g, 19.4 mmol), LiCl (112 mg, 2.67 mmol) and **3c** (566 mg, 0.76 mmol), yield: **10c** (82.0 mg, 52.7 µmol, 8%) and **11** (60.0 mg, 26.6 µmol, 4%) as brown solids. **10c**: **^{1}H NMR** ($CDCl_3$, 400 MHz): δ = 0.84–0.89 (m, 9 H, CH_2-C*H*$_3$), 1.18–1.36 (m, 60 H, C*H*$_2$), 1.41–1.49 (m, 6 H, C*H*$_2$-CH_2-CH_2O), 1.67–1.82 (m, 6 H, C*H*$_2$-CH_2O), 3.60 (s, 12 H, 2-OC*H*$_3$), 3.80 (s, 12 H, 4-OC*H*$_3$), 3.97–4.06 (m, 6 H, C*H*$_2$O), 4.41 (s, 8 H, 7-H), 6.38–6.46 (m, 8 H, 3-H, 5-H), 7.00–7.10 (m, 6 H, *o*-H, 6-H), 7.33 (s, 2 H, 2'-H), 7.41 (m, 2 H, *m*-H), 12.07 (s, 1 H, N*H*) ppm; **^{13}C NMR** ($CDCl_3$, 101 MHz): δ = 14.1 (CH_2-*C*H_3), 22.7, 26.1, 29.3, 29.4, 29.6, 29.7, 31.9 (*C*H_2), 50.2 (C-7), 55.1, 55.4 (2-O*C*H_3, 4-O*C*H_3), 69.2 (3'-O*C*H_2), 73.6 (4'-O*C*H_2), 98.9 (C-3), 103.9 (C-5), 108.4 (C-2'), 110.5 (C-7'), 119.4 (C-6'), 123.8 (C-1), 120.2 (C-*o*), 122.5 (C-*m*), 131.2 (C-6), 138.9, 142.9 (C-*i*, C-*p*), 146.9 (C-4'), 152.9 (C-3'), 159.2 (C-4), 161.3 (C-2) ppm; **FT-IR**: $\tilde{\nu}$ = 2921 (m), 2851 (w), 1731 (w), 1692 (w), 1611 (w), 1587 (w), 1540 (w), 1507 (w), 1465 (w), 1432 (w), 1334 (w), 1292 (w), 1265 (w), 1188 (w), 1157 (w), 1114 (m), 1029 (w), 936 (m), 834 (w), 784 (w), 725 (w), 640 (w), 584 (w) cm^{-1}; **MS** (ESI): *m/z* = 1517.01 [M–Cl^-] ($C_{96}H_{138}N_3O_{13}^+$). **HRMS** (ESI): *m/z* calcd. for [M–Cl^-] 1517.0224 ($C_{96}H_{138}N_3O_{13}^+$), found: 1517.0059; Anal. calcd. for $C_{96}H_{138}ClN_3O_{13}$ (1553.60): C 72.67, H 8.95, N 2.70; found: C 72.56, H 8.75, N 2.62.
DSC: Cr 103 °C [149.1 kJ mol^{-1}] SmA 117 °C [2.4 kJ mol^{-1}] I.

(4-{[3,4,5-Tris(tetradecyloxy)benzoyl]oxy}phenylamino)-bis[2,3-tetrakis(2,4-dimeth-oxybenzyl)]cyclopropenium dichloride (11): Mp 42 °C. **^{1}H NMR** ($CDCl_3$, 400 MHz): δ = 0.84–0.90 (m, 9 H, CH_2-C*H*$_3$), 1.22–1.36 (m, 60 H, C*H*$_2$), 1.40–1.50 (m, 6 H, C*H*$_2$-CH_2-CH_2O), 1.68–1.83 (m, 6 H, C*H*$_2$-CH_2O), 3.39 (s, 12 H, OC*H*$_3$), 3.75 (s, 12 H, OC*H*$_3$), 3.76 (s, 12 H, OC*H*$_3$), 3.86 (s, 12 H, OC*H*$_3$), 3.95–4.06 (m, 6 H, C*H*$_2$O), 4.11 (s, 8 H, 7-H), 4.41 (s, 8 H, 7-H), 6.18–6.20 (m, 4 H, 3-H), 6.34–6.37 (m, 4 H, 5-H), 6.44–6.47 (m, 4 H, 3-H), 6.50–6.56 (m, 4 H, 5-H), 6.89 (m, 4 H, 6-H), 7.17 (m, 4 H, 6-H), 7.28 (s, 2 H, 2'-H), 7.33 (m, 2 H, *o*-H), 7.98 (m, *m*-H) ppm; **^{13}C NMR** ($CDCl_3$, 101 MHz): δ = 14.1 (CH_2-*C*H_3), 22.7, 26.1, 29.3, 29.4, 29.6, 29.7, 31.9 (*C*H_2), 50.8, 51.0 (C-7), 55.2, 55.5, 55.8, 56.0 (2-O*C*H_3, 4-O*C*H_3), 69.3 (3'-O*C*H_2), 73.6 (4'-O*C*H_2), 98.5, 98.7 (C-3), 104.3 (C-7'), 104.6, 105.1 (C-5), 108.6 (C-2'), 113.1, 113.5 (C-1), 124.4, 125.3 (C-*o*, C-*m*), 123.5 (C-1'), 125.5 (C-6'), 131.2, 132.2 (C-6), 136.9, 143.4 (C-*i*, C-*p*), 151.5 (C-4'), 153.0 (C-3'), 158.8, 158.9 (C-4), 161.5, 162.0 (C-2) ppm; **FT-IR**: $\tilde{\nu}$ = 3375 (s), 2971 (m), 2852 (w), 1957 (w), 1735 (w), 1611 (w), 1579 (w), 1508 (w), 1465 (w), 1434 (w), 1334 (w), 1292 (w), 1158 (w), 1115 (w), 1024 (m), 936 (m), 835 (w), 786 (w), 640 (w), 580 (w) cm^{-1}; **MS** (ESI): *m/z* = 1092.18 [M–2 Cl^-]

($C_{133}H_{181}N_5O_{21}^{2+}$), 2220.30 [M–Cl$^-$] ($C_{133}H_{181}N_5O_{21}Cl^+$); **HRMS** (ESI): *m/z* calcd. for [M–2 Cl$^-$] 1092.1809 ($C_{133}H_{181}N_5O_{21}^{2+}$), found: 1092.1619.

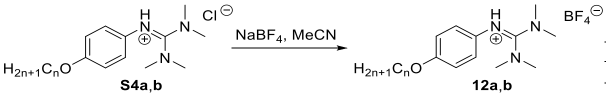

S4,12	a	b
n	14	16

Scheme S6

Synthesis of 4-alkoxyphenyl guanidinium tetrafluoroborates 12; General Procedure GP4. The respective guanidinium chloride **S4**[6] (1.20 mmol) was stirred with $NaBF_4$ (397 mg, 3.60 mmol) in MeCN (10 mL) at reflux for 1 h. The solids were filtered off and washed with hot MeCN (10 mL). The filtrate was concentrated under vacuum. The residue was dissolved in CH_2Cl_2 (7 mL) and filtered. After evaporation of the solvent, the pure product was obtained.

1,1,3,3-Tetramethyl-2-(4-(tetradecyloxy)phenyl)guanidinium tetrafluoroborate (12a): Yield: 478 mg, 973 µmol, 81%, pale brown solid. **^{1}H NMR** (400 MHz, $CDCl_3$): δ = 8.47 (s, 1H, NH), 6.96 (m, 2H, Ar-H), 6.87 (m, 2H, Ar-H), 3.91 (t, *J* = 6.5 Hz, 2H, OCH_2), 2.95 (br s, 12H, $N(CH_3)_2$), 1.76 (quint, *J* = 7.2 Hz, 2H, OCH_2CH_2), 1.49–1.19 (m, 22H, CH_2), 0.88 (t, *J* = 6.7 Hz, 3H, CH_3) ppm; **^{13}C NMR** (101 MHz, $CDCl_3$): δ = 158.9, 157.1 (*C*(NMe_2)$_2$, *p*-C), 129.9 (*i*-C), 122.4 (*o*-C), 115.7 (*m*-C), 68.4 (OCH_2), 40.2 ($N(CH_3)_2$), 31.9, 29.7, 29.7, 29.6, 29.6, 29.4, 29.4, 29.2, 26.0, 22.7 (CH_2), 14.1 (CH_3) ppm; **FT-IR**: $\tilde{\nu}$ = 3317 (w), 2917 (s), 2850 (m), 1640 (m), 1599 (w), 1567 (m), 1514 (m), 1468 (w), 1422 (w), 1402 (w), 1293 (w), 1241 (m), 1175 (w), 1114 (w), 1075 (s), 1001 (s), 914 (w), 831 (w), 722 (w), 522 (w) cm^{-1}; Anal. calcd. for $C_{25}H_{46}BF_4N_3O$ (491.47): C 61.10, H 9.43, N 8.55; found: C 60.97, H 9.52, N 8.55.

DSC: Cr 97 °C [50.1 kJ mol^{-1}] SmA 123 °C [0.8 kJ mol^{-1}] I.

1,1,3,3-Tetramethyl-2-(4-(hexadecyloxy)phenyl)guanidinium tetrafluoroborate (12b): Yield: 555 mg, 1.07 mmol, 89%, pale brown solid. **^{1}H NMR** (300 MHz, $CDCl_3$): δ = 8.47 (s, 1H, NH), 6.96 (m, 2H, Ar-H), 6.87 (m, 2H, Ar-H), 3.91 (t, *J* = 6.5 Hz, 2H, OCH_2), 2.95 (br s, 12H, $N(CH_3)_2$), 1.76 (quint, *J* = 7.2 Hz, 2H, OCH_2CH_2), 1.49–1.19 (m, 26H, CH_2), 0.88 (t, *J* = 6.7 Hz, 3H, CH_3) ppm; **^{13}C NMR** (75 MHz, $CDCl_3$): δ = 158.9, 157.1 (*C*(NMe_2)$_2$, *p*-C), 129.9 (*i*-C), 122.3 (*o*-C), 115.6 (*m*-C), 68.4 (OCH_2), 40.2 ($N(CH_3)_2$), 31.9, 29.7, 29.6, 29.6, 29.6, 29.4, 29.3, 29.2, 26.0, 22.7 (CH_2), 14.1 (CH_3) ppm; **FT-IR**: $\tilde{\nu}$ = 3321 (w), 2917 (s), 2850 (m), 1630 (m), 1599 (m), 1565 (m), 1512 (m), 1469 (m), 1421 (w), 1400 (w), 1292 (w), 1237 (m), 1172 (w), 1148 (w), 1052 (s), 1001 (s), 912 (w), 833 (w), 733 (w), 519 (w) cm^{-1};

Anal. calcd. for $C_{27}H_{50}BF_4N_3O$ (519.52): C 62.42, H 9.70, N 8.09; found: C 62.41, H 9.42, N 7.86.

DSC: Cr 96 °C [52.0 kJ mol^{-1}] SmA 150 °C [1.3 kJ mol^{-1}] I.

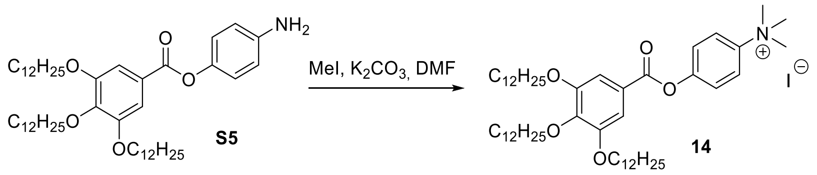

Scheme S7

***N,N,N*-Trimethyl-4-(3,4,5-tris(dodecyloxy)benzoyl)oxy)phenylammonium iodide (14)**

Following a reported procedure,[6] to a solution of known amine **S5**[5] (300 mg, 0.39 mmol) in DMF (10 mL), K_2CO_3 (190 mg, 1.37 mmol) and methyl iodide (1.00 g, 7.05 mmol) were added and the resulting mixture was stirred at room temperature for 3 d. The solvent was then removed *in vacuo*, the residue was dissolved in CH_2Cl_2 (20 mL) and filtered via a fritted funnel. The solid from the filtration was purified by chromatography on HI-treated SiO_2 (eluent: EtOAc, then CH_2Cl_2 / MeOH (3 : 1)). The residue was taken up in CH_2Cl_2 (100 mL) and filtered to remove traces of SiO_2. The filtrate was concentrated *in vacuo* and dried in a vacuum desiccator over P_2O_5. Yield: 250 mg, 267 µmol, 68%. **^{1}H-NMR** ($CDCl_3$, 300 MHz): δ = 0.82–0.95 (m, 9H, CH_3), 1.12–1.42 (m, 48H, CH_2), 1.42–1.55 (m, 6H, CH_2), 1.70–1.90 (m, 6H, $OCH_2C\mathit{H_2}$), 4.13–4.99 (m, 15H, N-$(CH_3)_3$, OCH_2), 7.36 (s, 2H, 2‘-H), 7.43–7.50 (m, 2H, 2-H), 8.10–8.17 (m, 2H, 3‘-H) ppm. **^{13}C-NMR** (75 MHz, $CDCl_3$): δ = 14.1 (CH_3), 22.7, 26.1, 26.1, 29.3, 29.4, 29.4, 29.4, 29.6, 29.7, 29.7, 29.7, 29.8, 30.4, 31.9, (CH_2), 58.1 (N-$(CH_3)_3$), 69.4, 73.7 (OCH_2), 108.7 (C-2), 121.8 (C-2‘), 122.7 (C-1), 124.1 (C-3‘), 143.6 (C-4‘), 144.2 (C-4), 152.2 (C-1‘), 153.1 (C-3), 164.4 (C=O) ppm. **FT-IR**: $\tilde{\nu}$ = 3145 (w), 2920 (vs), 2852 (s), 2157 (w), 2014 (w), 1731 (m), 1666 (w), 1585 (m), 1500 (m), 1446 (m), 1430 (s), 1386 (w), 1335 (vs), 1225 (s), 1192 (vs), 1115 (vs), 1013 (m), 949 (m), 851 (m), 819 (w), 781 (w), 751 (m), 721 (w), 661 (w), 559 (w) cm^{-1}. MS (ESI$^+$): *m/z* = 808.67 [M – I$^-$] ($C_{52}H_{90}NO_5^+$); **MS** (ESI$^-$): *m/z* = 126.91 [I]$^-$; **HRMS** (ESI$^+$): *m/z* calcd. for [M – I$^-$] 808.6814 ($C_{52}H_{90}NO_5^+$), found: 808.6821; **HRMS** (ESI$^-$): *m/z* calcd. for [I$^-$] 126.9050, found: 126.9039; Anal. Calcd. for $C_{52}H_{90}INO_5$(936.20): C 66.71, H 9.69, N 1.50; found: C 66.43, H 8.86, N 1.37.

DSC: Cr 31.0 °C [–47 kJ/mol], Col$_h$ 139 °C [–19 kJ/mol], I.

3) References

[1] Yoshida, Z.-I.; Tawara, Y. Aminocyclopropenium Ion. *J. Am. Chem. Soc.* **1971**, *93*, 2573–2574.

[2] Hawkes, G. E.; Smith, R. A.; Roberts, J. D. Nuclear Magnetic Resonance Spectroscopy. Carbon-13 Chemical Shifts of Chlorinated Organic Compounds. *J. Org. Chem.* **1974**, *39*, 1276–1290.

[3] Mishiro, K.; Hu, F.; Paley, D. W.; Min, W.; Lambert, T. H. Macrosteres: The Deltic Guanidinium Ion. *Eur. J. Org. Chem.* **2016**, 1655–1659.

[4] Butschies, M.; Haenle, J. C.; Tussetschläger, S.; Laschat, S. Liquid crystalline guanidinium phenylalkoxybenzoates: towards room temperature liquid crystals via bending of the mesogenic core and the use of triflate counter ions. *Liq. Cryst.* **2013**, *40*, 52–71.

[5] Butschies, M.; Frey, W.; Laschat, S. Designer Ionic Liquid Crystals Based on Congruently Shaped Guanidinium Sulfonates. *Chem. Eur. J.* **2012**, *18*, 3014–3022.

[6] Sauer, S.; Saliba, S.; Tussetschläger, S.; Baro, A.; Frey, W.; Giesselmann, F.; Laschat, S.; Kantlehner, W. *p*-Alkoxybiphenyls with guanidinium head groups displaying smectic mesophases. *Liq. Cryst.* **2009**, *36*, 275–299.

4) Differential Scanning Calorimetry (DSC) Curves

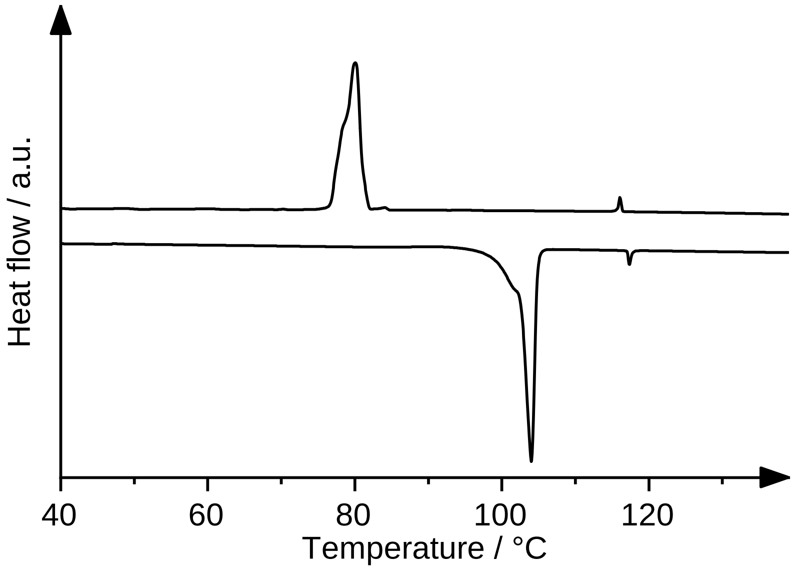

Figure S1. DSC traces of **7b**, 2nd heating/cooling cycle.

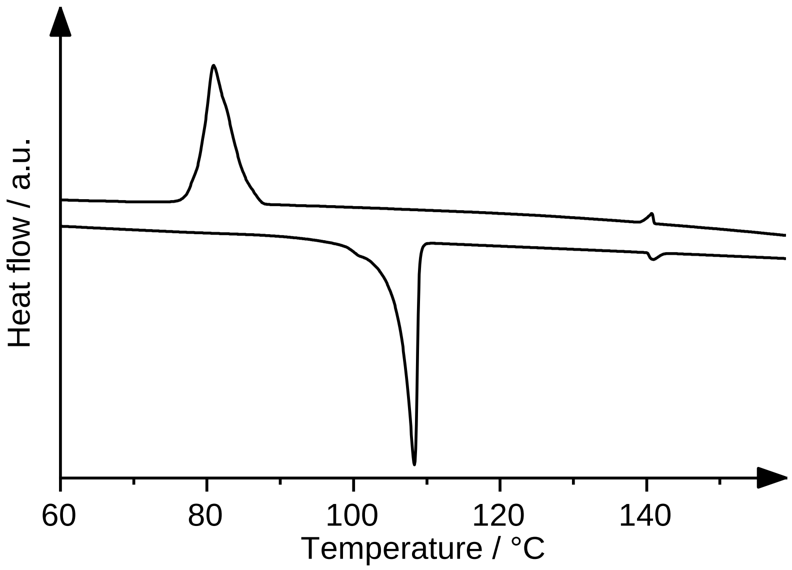

Figure S2. DSC traces of **7c**, 2nd heating/cooling cycle.

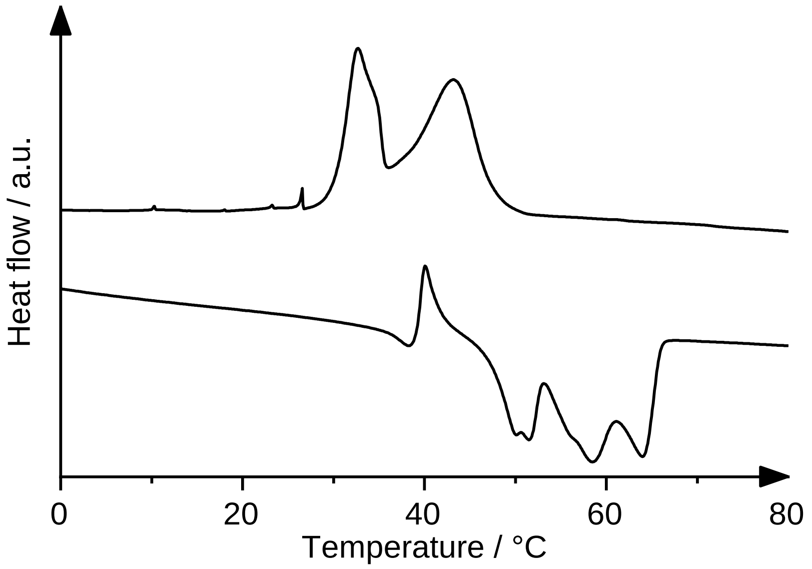

Figure S3. DSC traces of **9**, 2nd heating/cooling cycle.

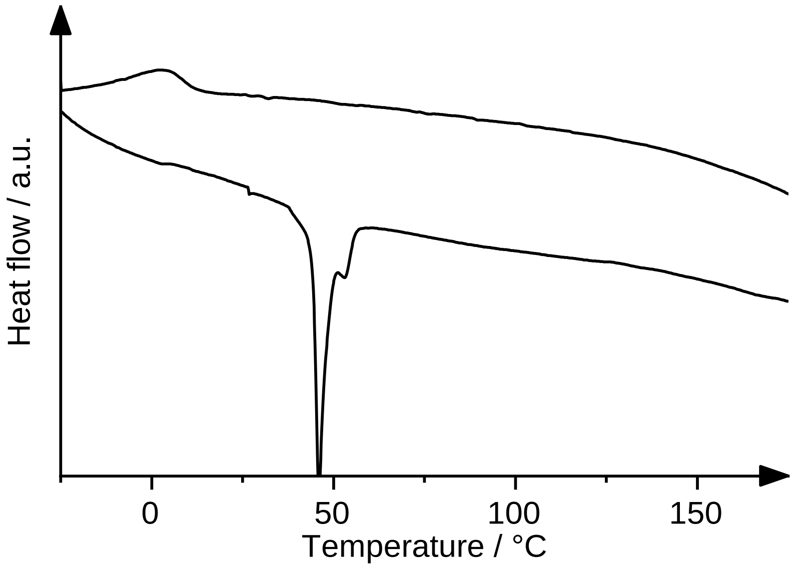

Figure S4. DSC traces of **10a**, 1st heating/cooling cycle.

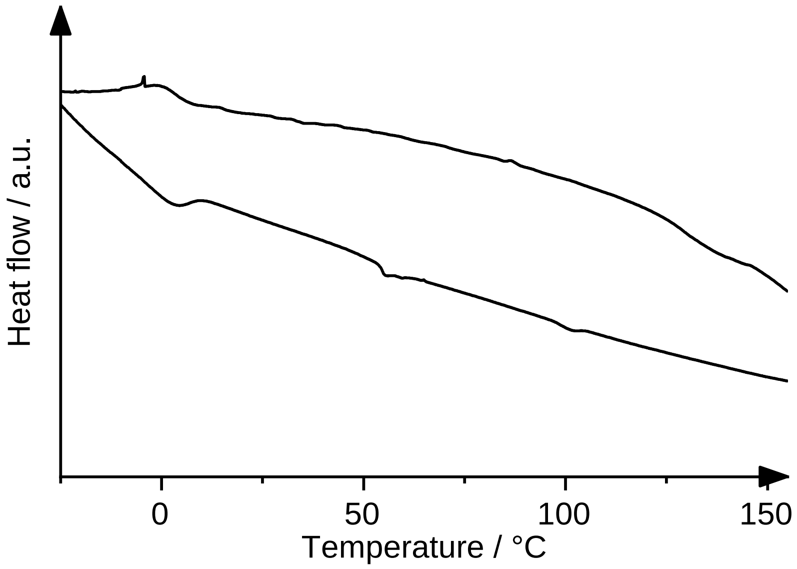

Figure S5. DSC traces of **10c**, 2nd heating/cooling cycle.

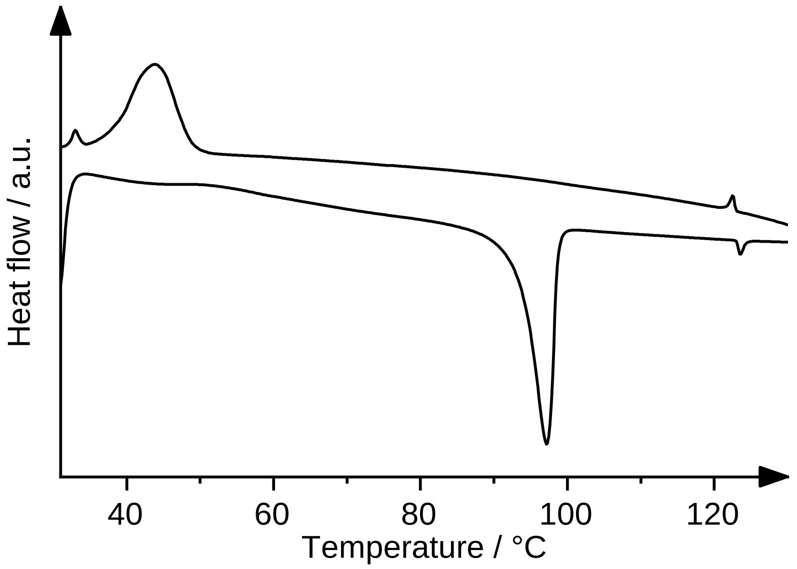

Figure S6. DSC traces of **12a**, 2nd heating/cooling cycle.

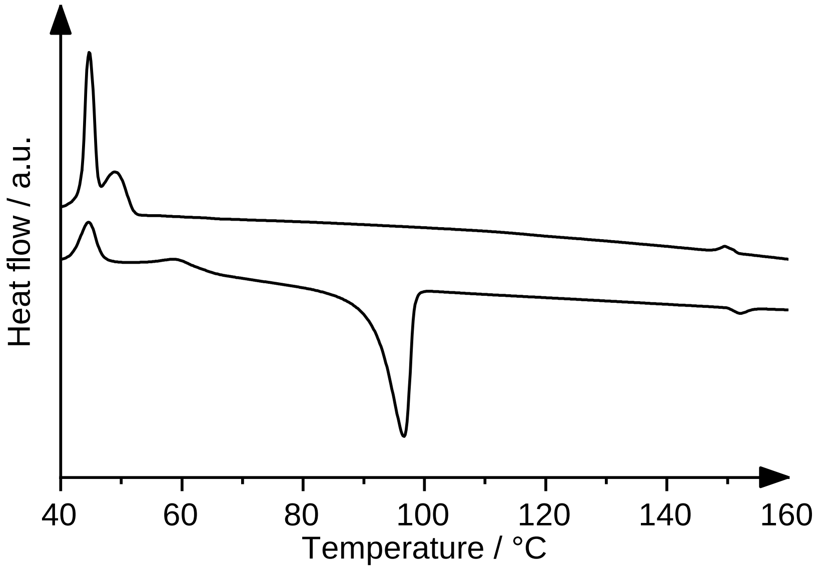

Figure S7. DSC traces of **12b**, 2nd heating/cooling cycle.

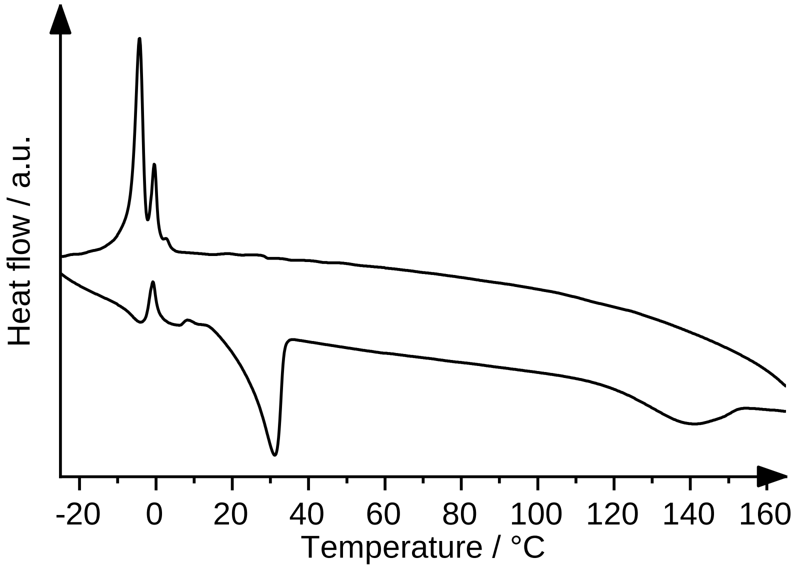

Figure S8. DSC traces of **14**, 2nd heating/cooling cycle.

5) Polarizing Optical Microscopy (POM) of Derivatives 9, 12 and 14

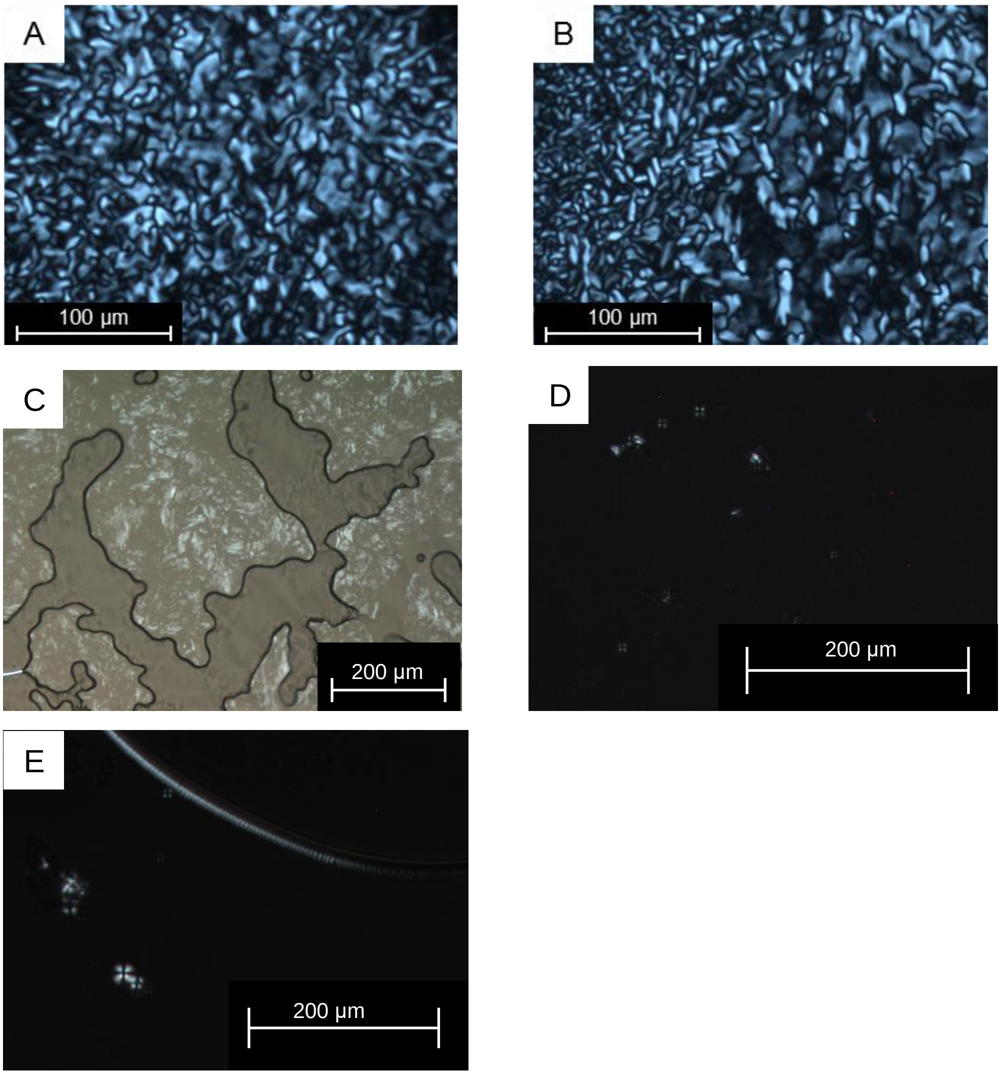

Figure S9. Texture of ammonium derivative **9** as seen between cross polarizers upon cooling from the isotropic liquid at 57 °C (A) and at 48 °C (B); texture of trimethylammonium derivative **14** upon cooling from the isotropic liquid at 20 °C after shearing (C); texture of tetramethyl guanidinium derivative **12a** upon cooling from the isotropic liquid at 160 °C (D) and texture of tetramethyl guanidinium derivative **12b** upon cooling from the isotropic liquid at 120 °C (E) (cooling rate 5 K min^{-1}, magnification ×200).

6) X-Ray Diffraction (XRD) Measurement of Derivatives 9, 10a, 12 and 14

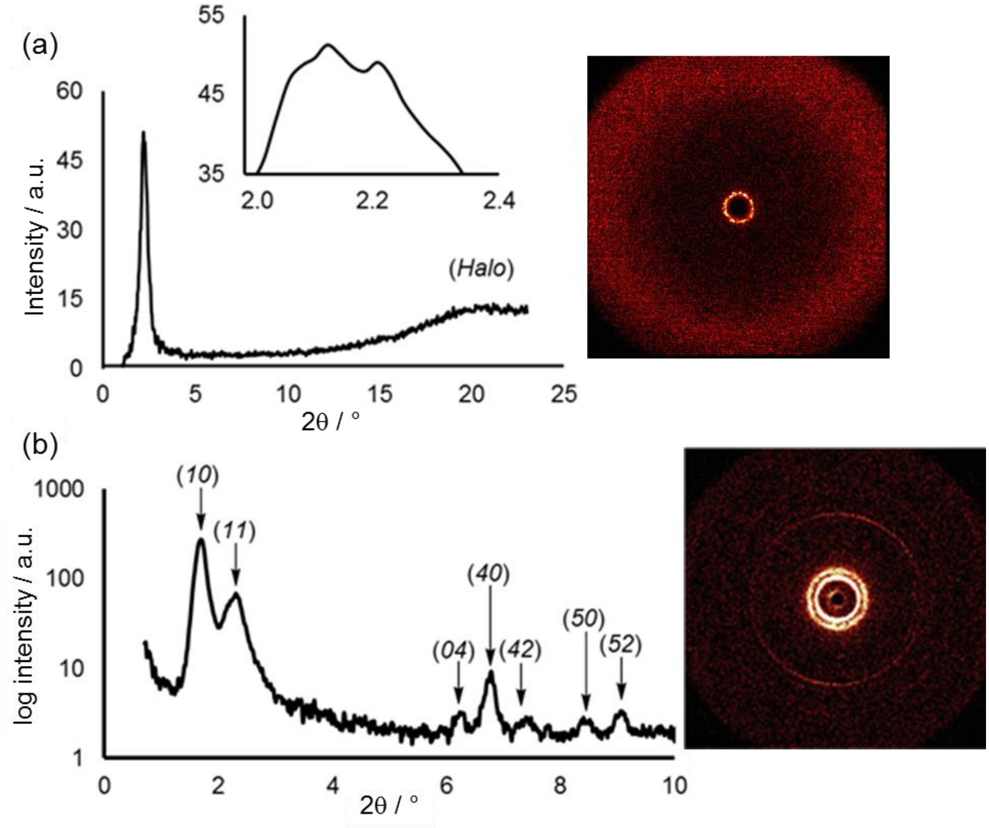

Figure S10. (a) WAXS scattering profile and diffraction image of **9** at 55 °C upon cooling from the isotropic layer. Inset: detail of the peak at 2.2°. (b) SAXS profile and diffraction image of **9** at 55 °C upon cooling from the isotropic liquid.

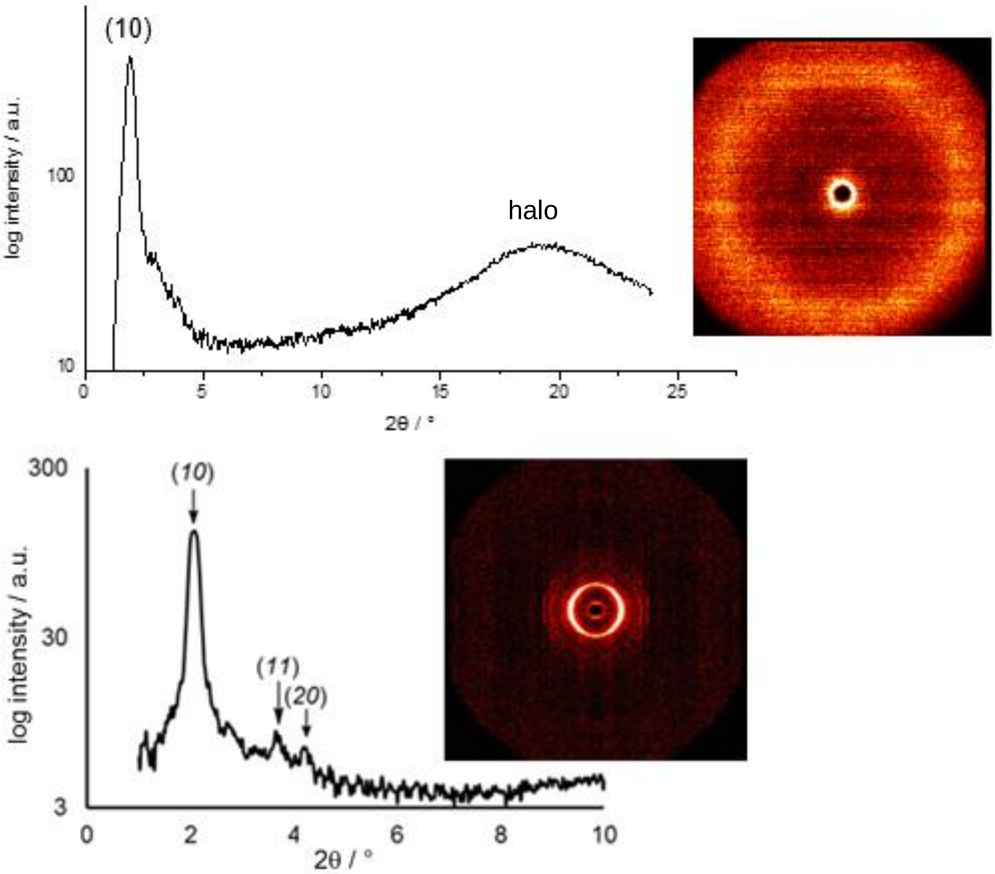

Figure S11. (a) WAXS scattering profile and diffraction image of **10a** at 160 °C upon first heating. (b) SAXS profile and diffraction image of **10a** at 160 °C upon first heating.

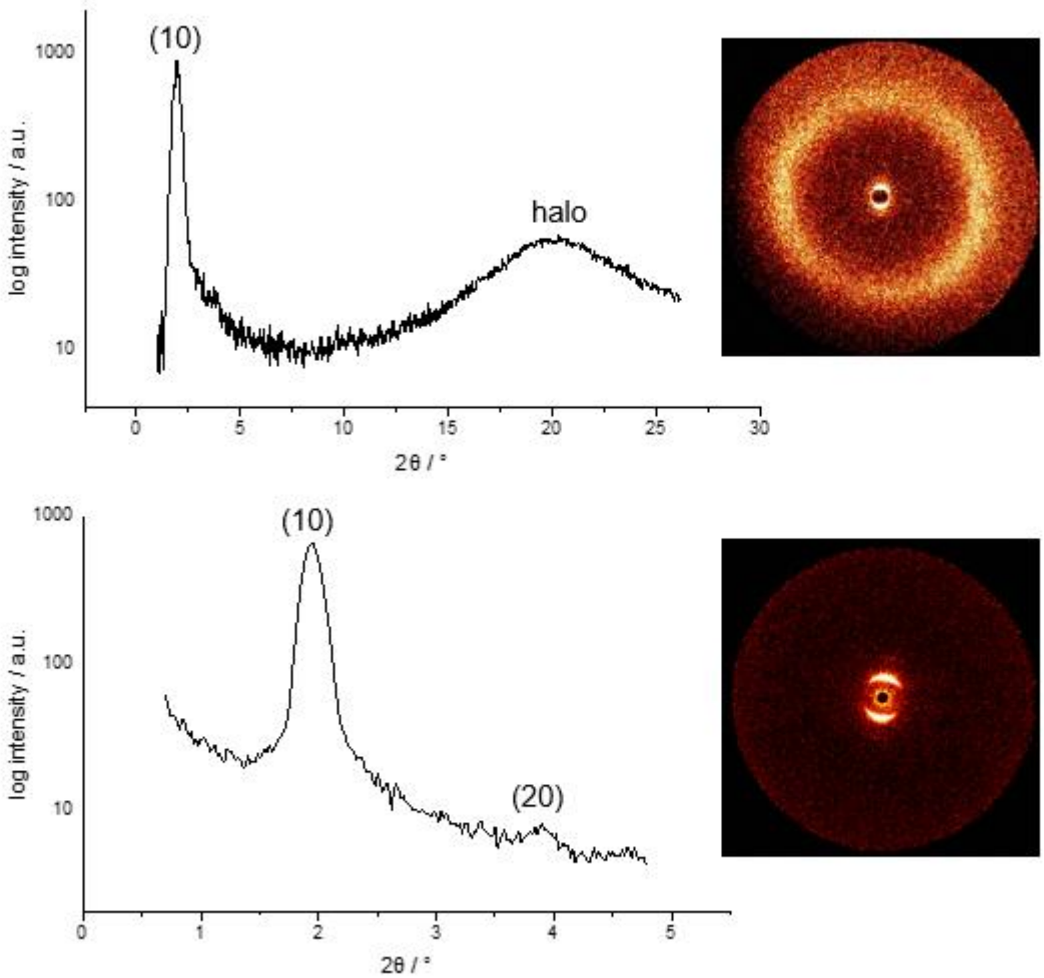

Figure S12. (a) WAXS scattering profile and diffraction image of **14** at 31 °C upon cooling from the isotropic layer. (b) SAXS scattering profile and diffraction image of **14** at 55 °C upon cooling from the isotropic liquid.

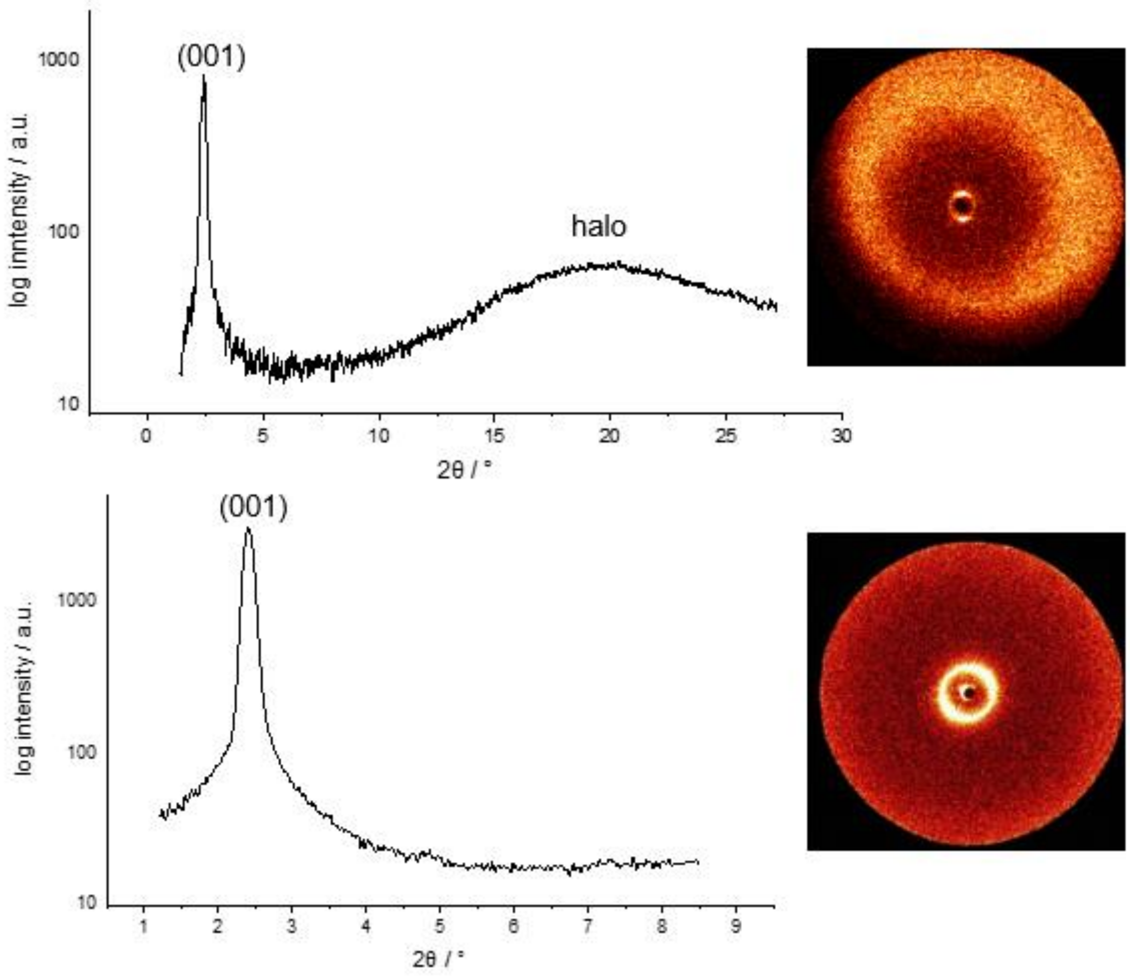

Figure S13. (a) WAXS scattering profile and diffraction image of **12a** at 115 °C upon cooling from the isotropic layer. (b) SAXS scattering profile and diffraction image of **12a** at 90 °C upon cooling from the isotropic liquid.

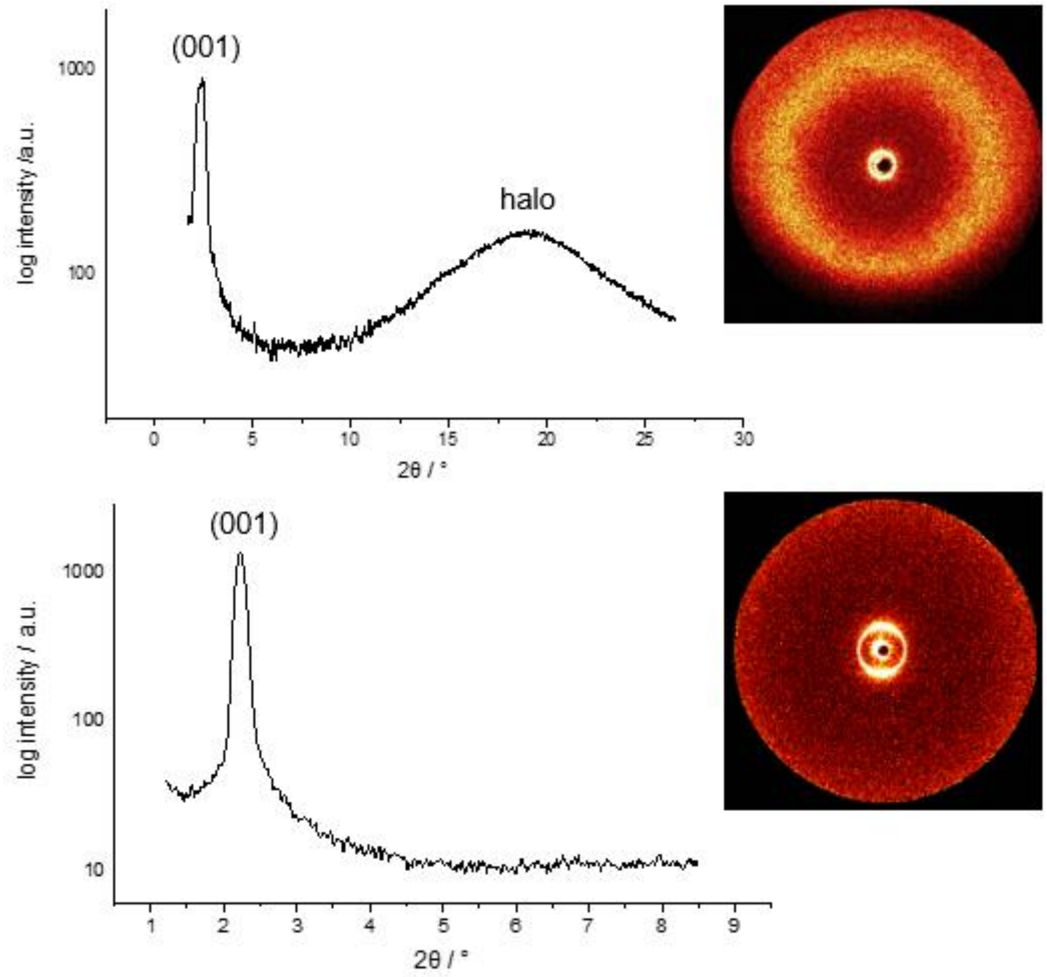

Figure S14. (a) WAXS scattering profile and diffraction image of **12b** at 120 °C upon cooling from the isotropic layer. (b) SAXS scattering profile and diffraction image of **12b** at 90 °C upon cooling from the isotropic liquid.

7) X-Ray Single-crystal Structure Analysis

Table S1. Crystal data and structure refinement for derivative **8a**.

	**8a**
empirical formula	$C_{33}H_{58}BF_4N_3O$
formula weight	599.63
temperature (K)	130(2)
wavelength (Å)	0.71073
crystal system	monoclinic
space group	$P2_1/c$
unit cell dimensions (Å)	a = 20.8139(10)
	b = 9.6856(4)
	c = 18.1395(8)
	α = 90°
	β = 105.126(2)°
	γ = 90°
volume V (Å^3)	3530.1(3)
Z	4
density D_c (calcd. g/cm^3)	1.128
μ (mm^{-1})	0.082
F(000)	1304
crystal size (mm^3)	0.38 × 0.21 × 0.17
θ range for data collection (deg)	2.03–28.37
index ranges	$-27 \leq h \leq 26$, $-12 \leq k \leq 12$, $-24 \leq l \leq 24$
reflns collected	32726
reflns unique	8781 [R_{int} = 0.0330]
completeness to θ = 28.37	99.5%
refinement method	full-matrix least-squares on F^2
data/restraints/parameters	8781/0/392
GOF on F^2	1.040
final R indices (I > 2α(I))	R_1 = 0.0413, wR_2 = 0.1002
R indices (all data)	R_1 = 0.0608, wR_2 = 0.1070
largest diff. peak and hole (e/Å^3)	0.345, –0.231

There is evidence of a strong intermolecular hydrogen bond interaction between the N1-H1 as H-donor and F3 as H-acceptor of the BF_4^- anion, with a F3···H1 distance of 2.05 Å and a N1-H1···F3 angle of 167°. Further stabilization of the anion is observed by more weak interaction between C21-H21, C17-H17 and C6-H6 as donors and the F atoms of the anion as acceptor. The range of the H···F distances is between 2.36 Å and 2.55 Å and the interval of the angles is 137° to 158° (Figure S4).

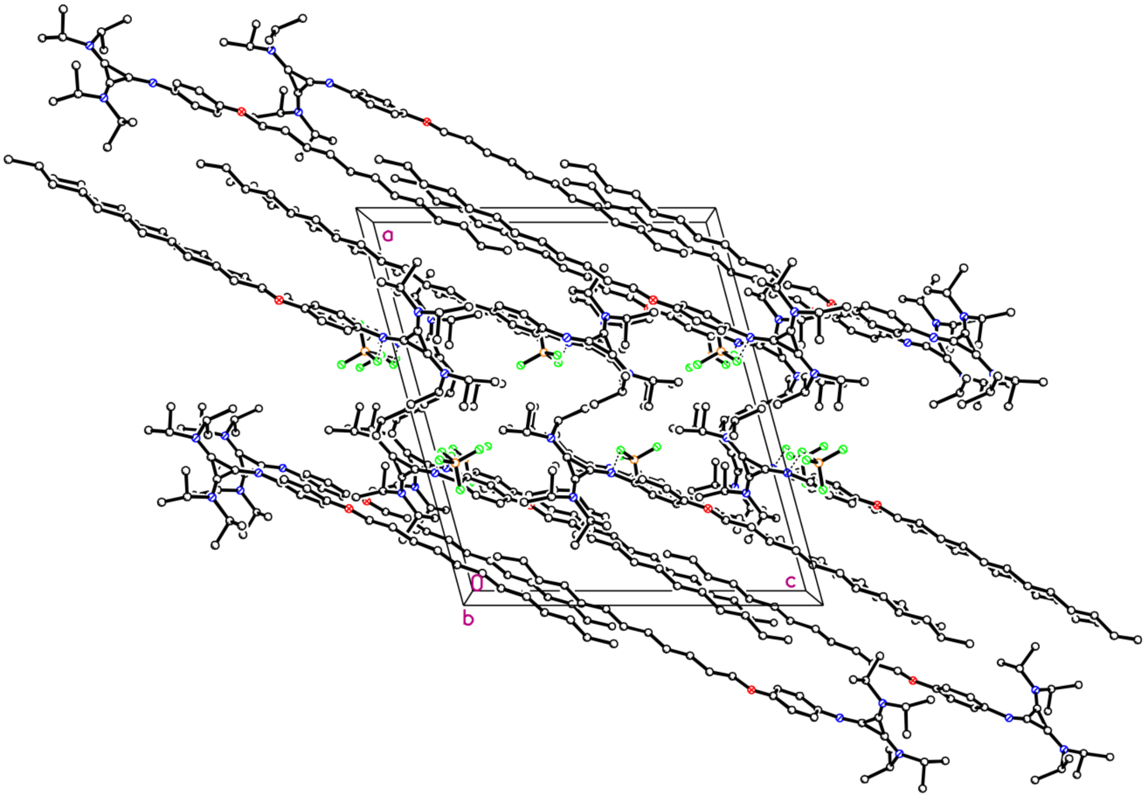

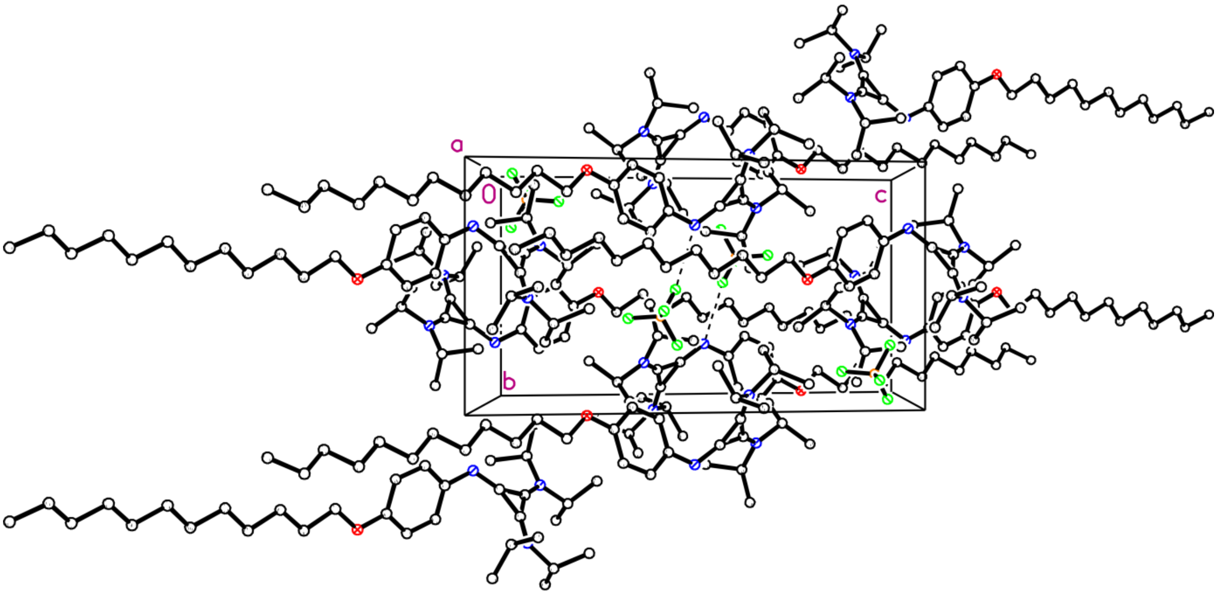

Figure S15. Packing diagrams of 1,2-bis(diisopropylamino)cyclopropenium tetrafluoroborate **8a** viewed along the b axis (above) and the a axis (below).

8) NMR Data of New Compounds

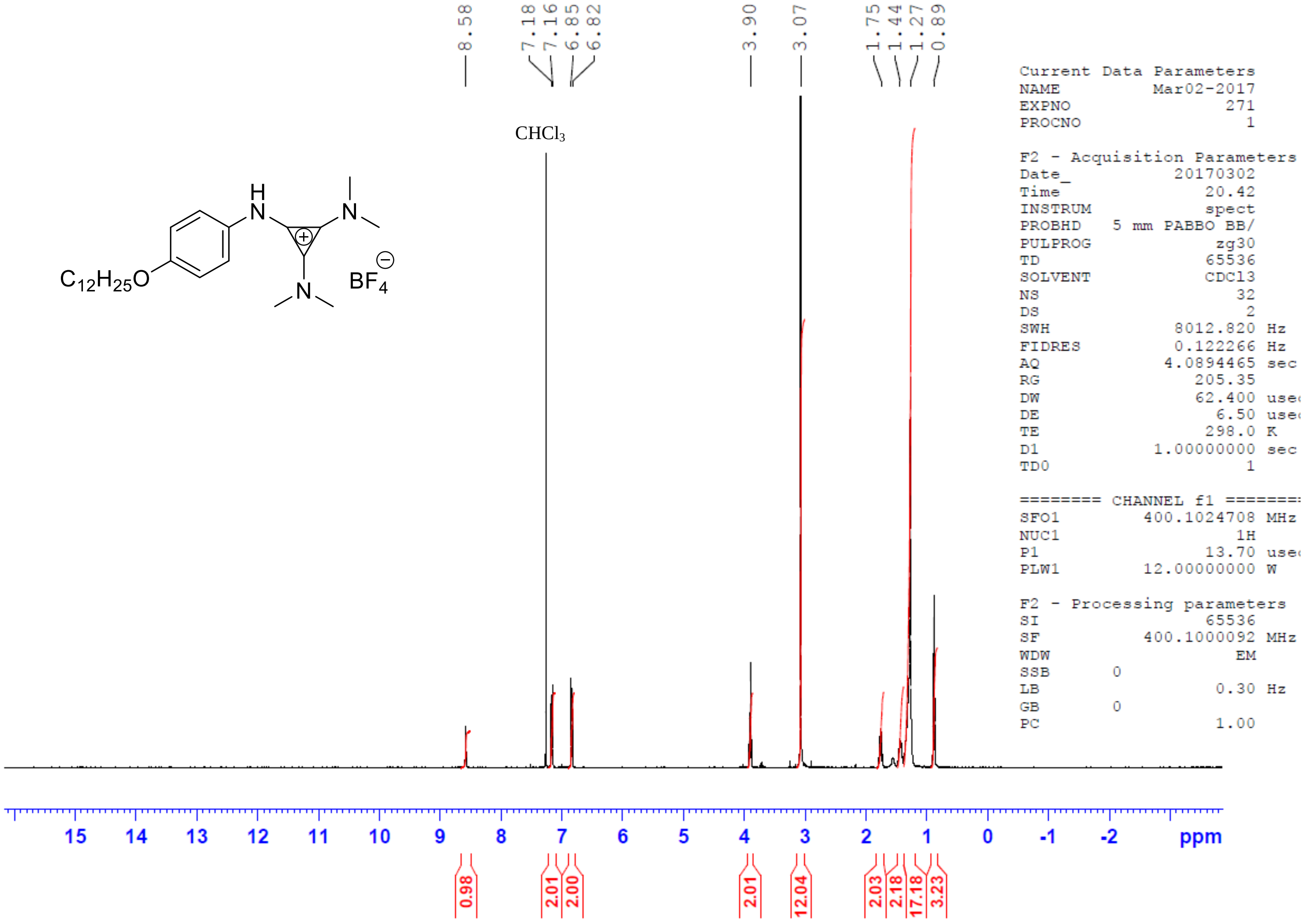

^{1}H NMR spectrum of **7a**.

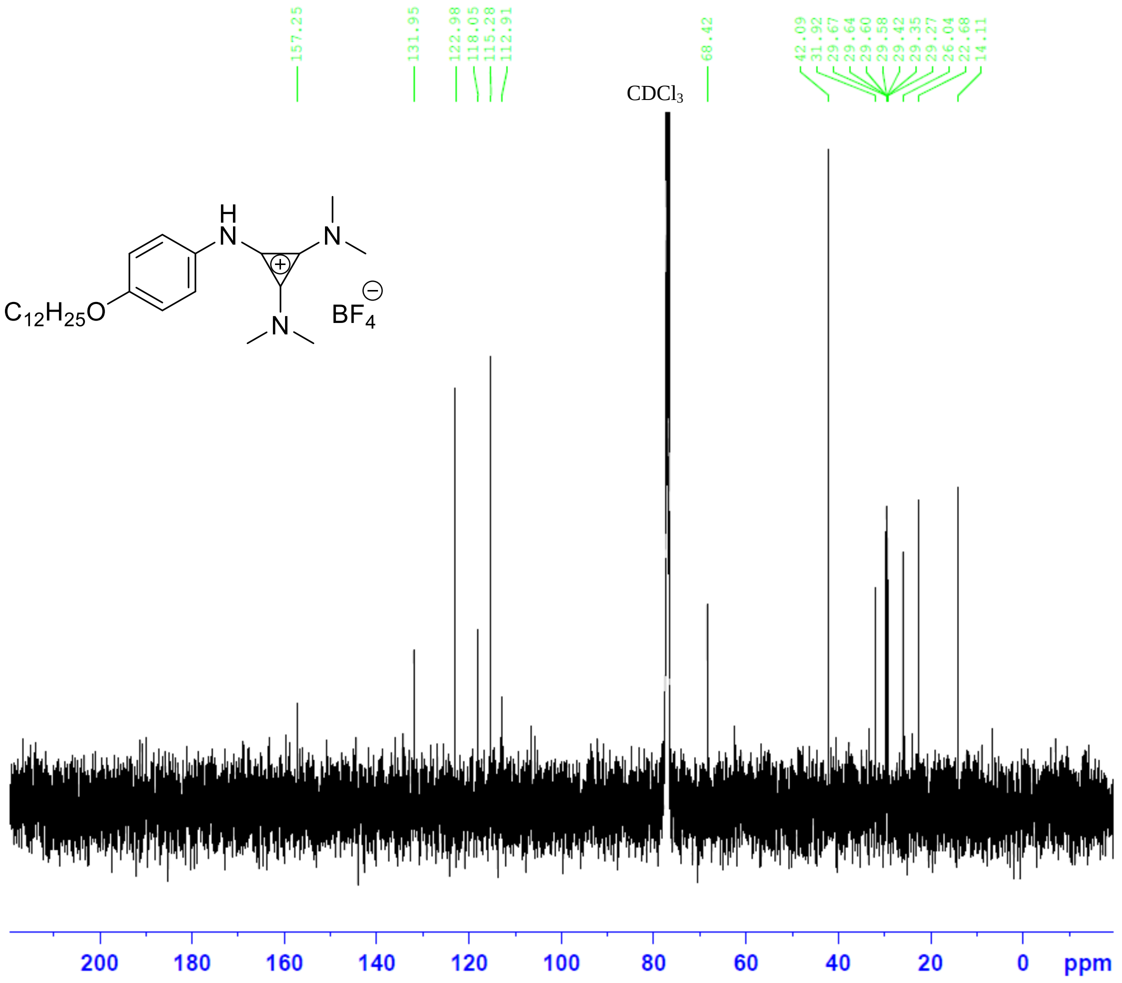


```
Current Data Parameters
NAME              Mar02-2017
EXPNO                    275
PROCNO                     1

F2 - Acquisition Parameters
Date_             20170303
Time                  0.26
INSTRUM              spect
PROBHD       5 mm PABBO BB/
PULPROG            zgpg30
TD                   65536
SOLVENT              CDCl3
NS                    1024
DS                       4
SWH              24038.461 Hz
FIDRES            0.366798 Hz
AQ               1.3631488 sec
RG                  205.35
DW                  20.800 usec
DE                    6.50 usec
TE                   298.0 K
D1              8.00000000 sec
D11             0.03000000 sec
TD0                      1

======== CHANNEL f1 ========
SFO1          100.6152851 MHz
NUC1                   13C
P1                   10.00 usec
PLW1           48.00000000 W

======== CHANNEL f2 ========
SFO2          400.1016004 MHz
NUC2                    1H
CPDPRG[2           waltz16
PCPD2                90.00 usec
PLW2           12.00000000 W
PLW12           0.27805999 W
PLW13           0.22522999 W

F2 - Processing parameters
SI                   32768
SF            100.6052250 MHz
WDW                     EM
```

^{13}C NMR spectrum of **7a**.

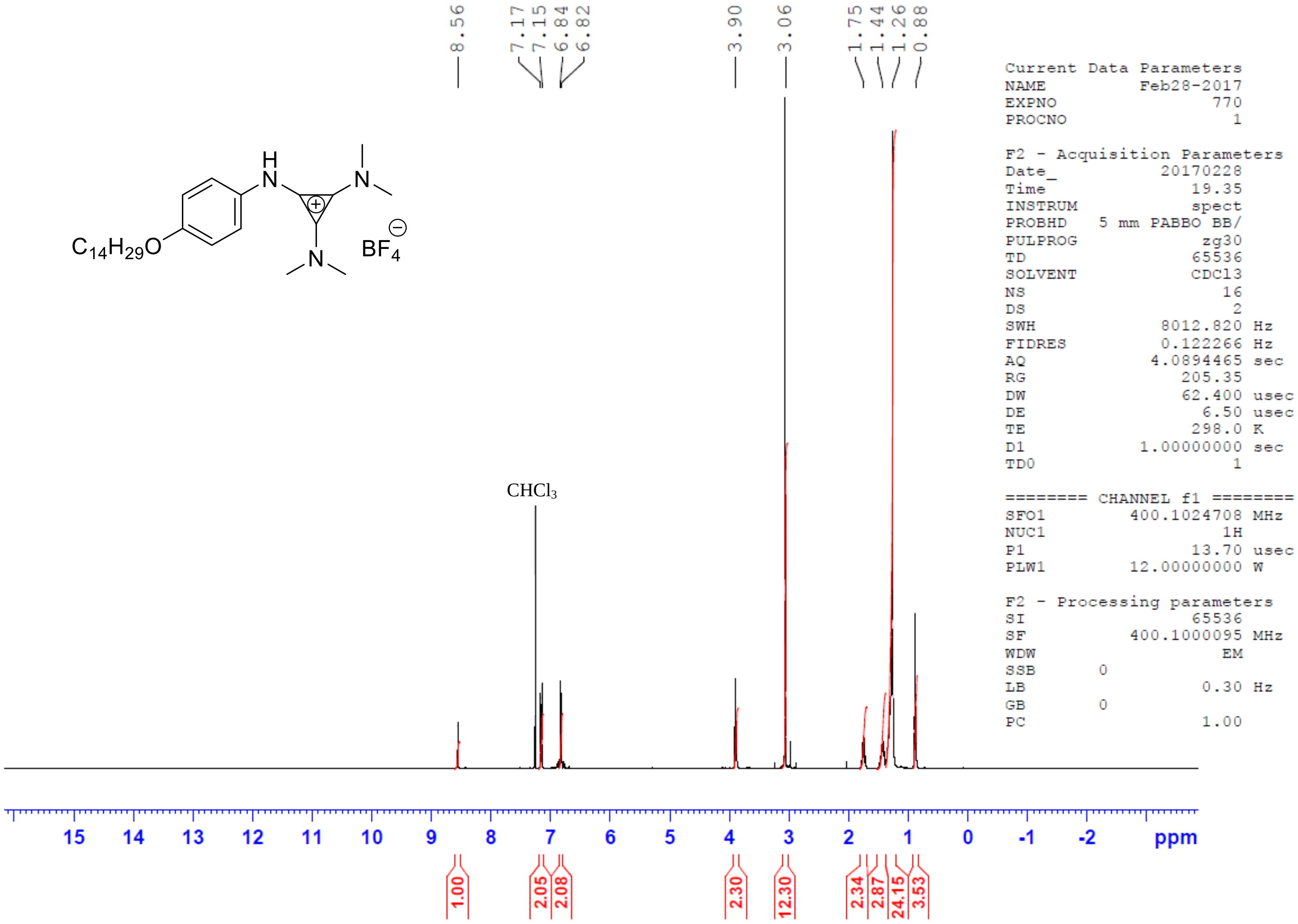

^{1}H NMR spectrum of **7b**.

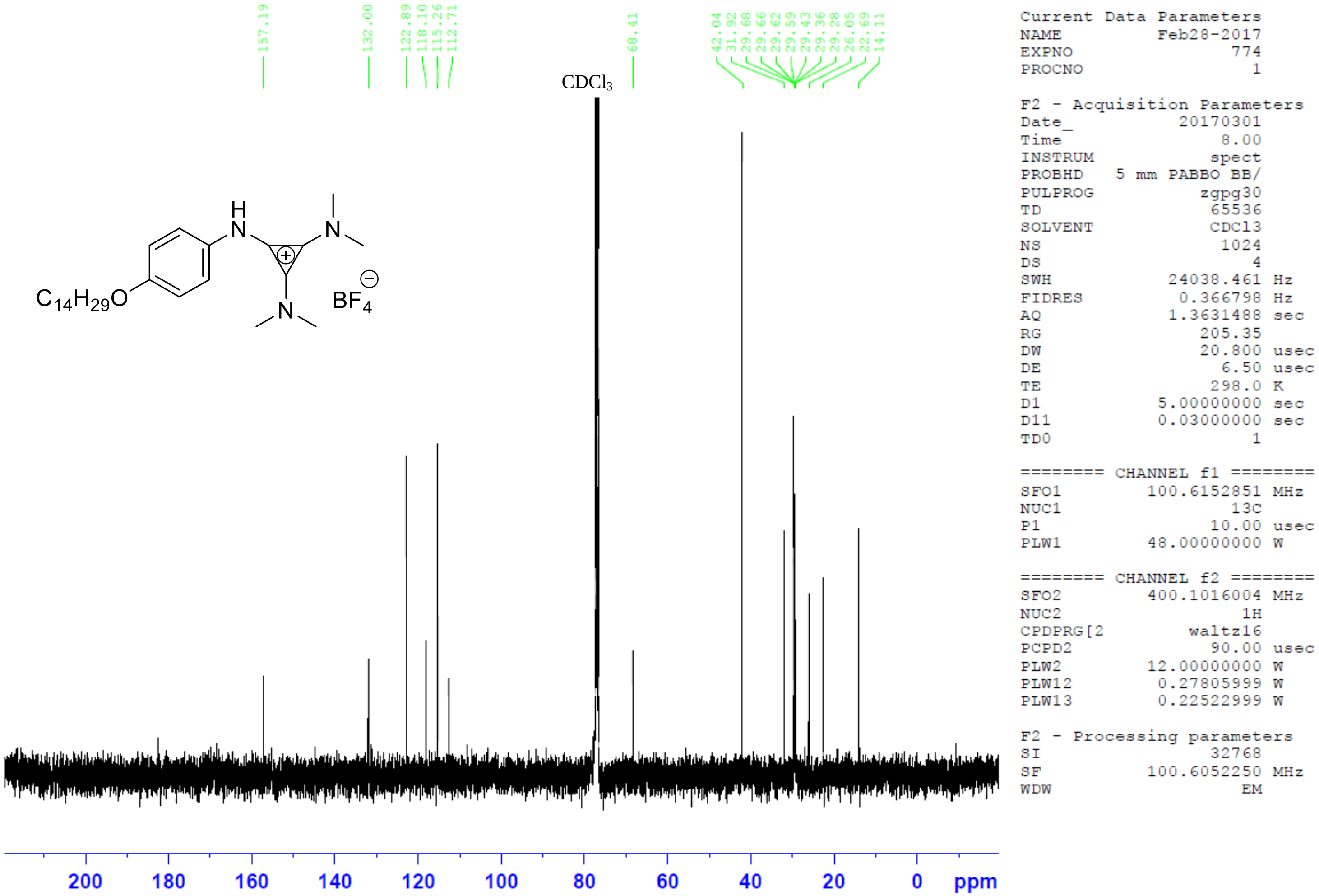

^{13}C NMR spectrum of **7b**.

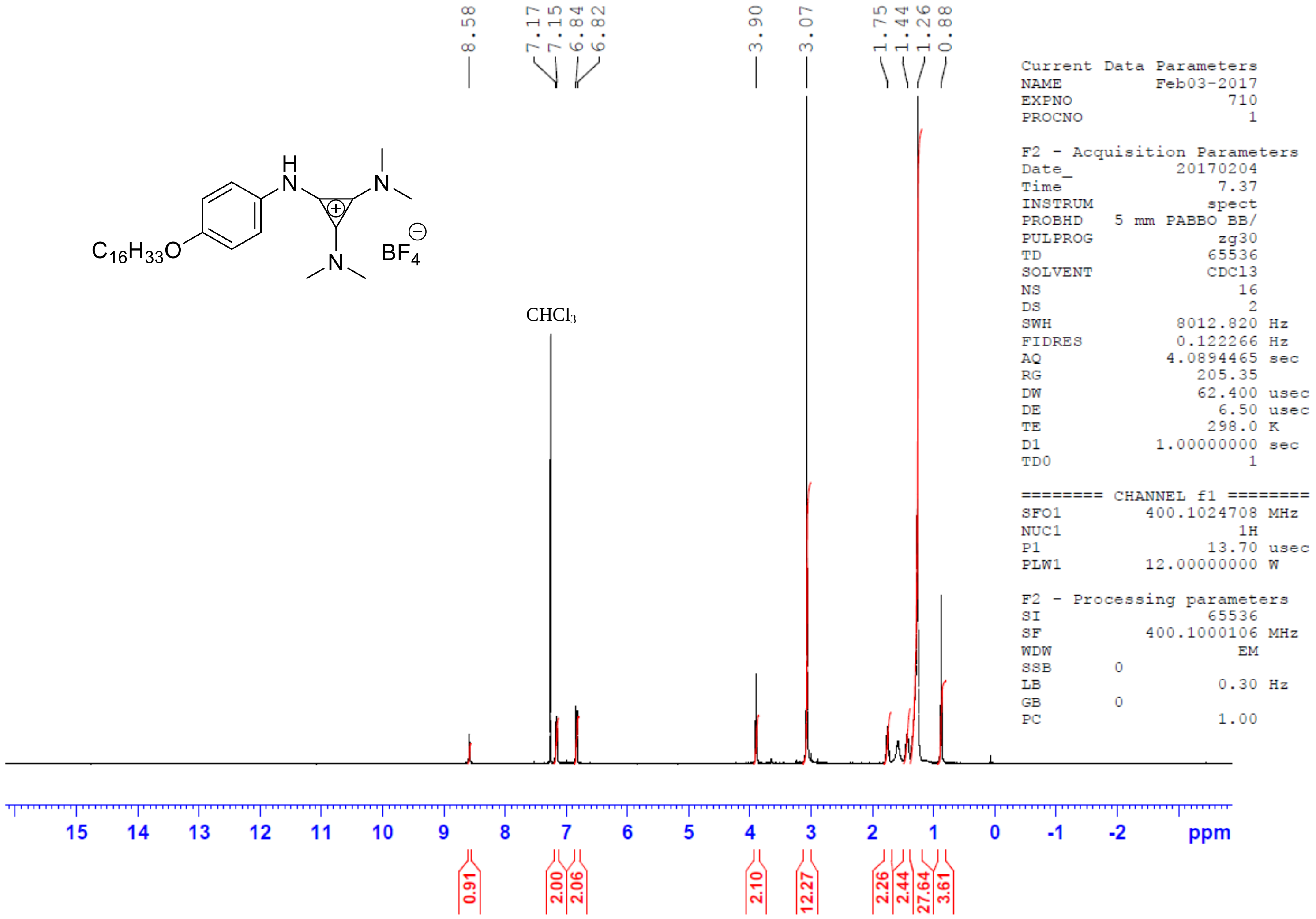

^{1}H NMR spectrum of **7c**.

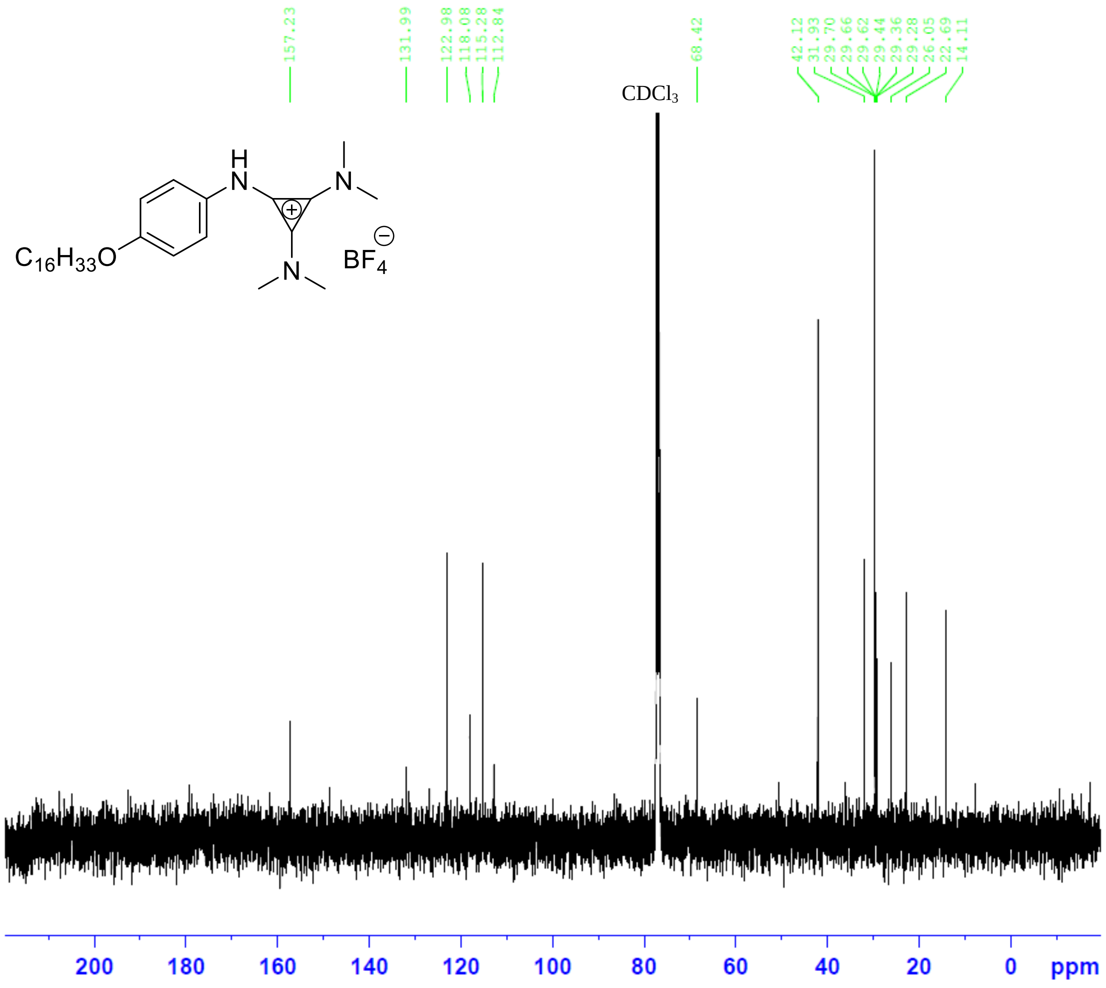


```
Current Data Parameters
NAME              Feb03-2017
EXPNO                    714
PROCNO                     1

F2 - Acquisition Parameters
Date_              20170205
Time                   6.15
INSTRUM               spect
PROBHD    5 mm PABBO BB/
PULPROG             zgpg30
TD                   65536
SOLVENT              CDCl3
NS                    1024
DS                       4
SWH              24038.461 Hz
FIDRES            0.366798 Hz
AQ               1.3631488 sec
RG                  205.35
DW                  20.800 usec
DE                    6.50 usec
TE                   298.0 K
D1              7.00000000 sec
D11             0.03000000 sec
TD0                      1

======== CHANNEL f1 ========
SFO1           100.6152851 MHz
NUC1                   13C
P1                   10.00 usec
PLW1           48.00000000 W

======== CHANNEL f2 ========
SFO2           400.1016004 MHz
NUC2                    1H
CPDPRG[2           waltz16
PCPD2                90.00 usec
PLW2           12.00000000 W
PLW12           0.27805999 W
PLW13           0.22522999 W

F2 - Processing parameters
SI                   32768
SF             100.6052250 MHz
WDW                     EM
```

^{13}C NMR spectrum of **7c**.

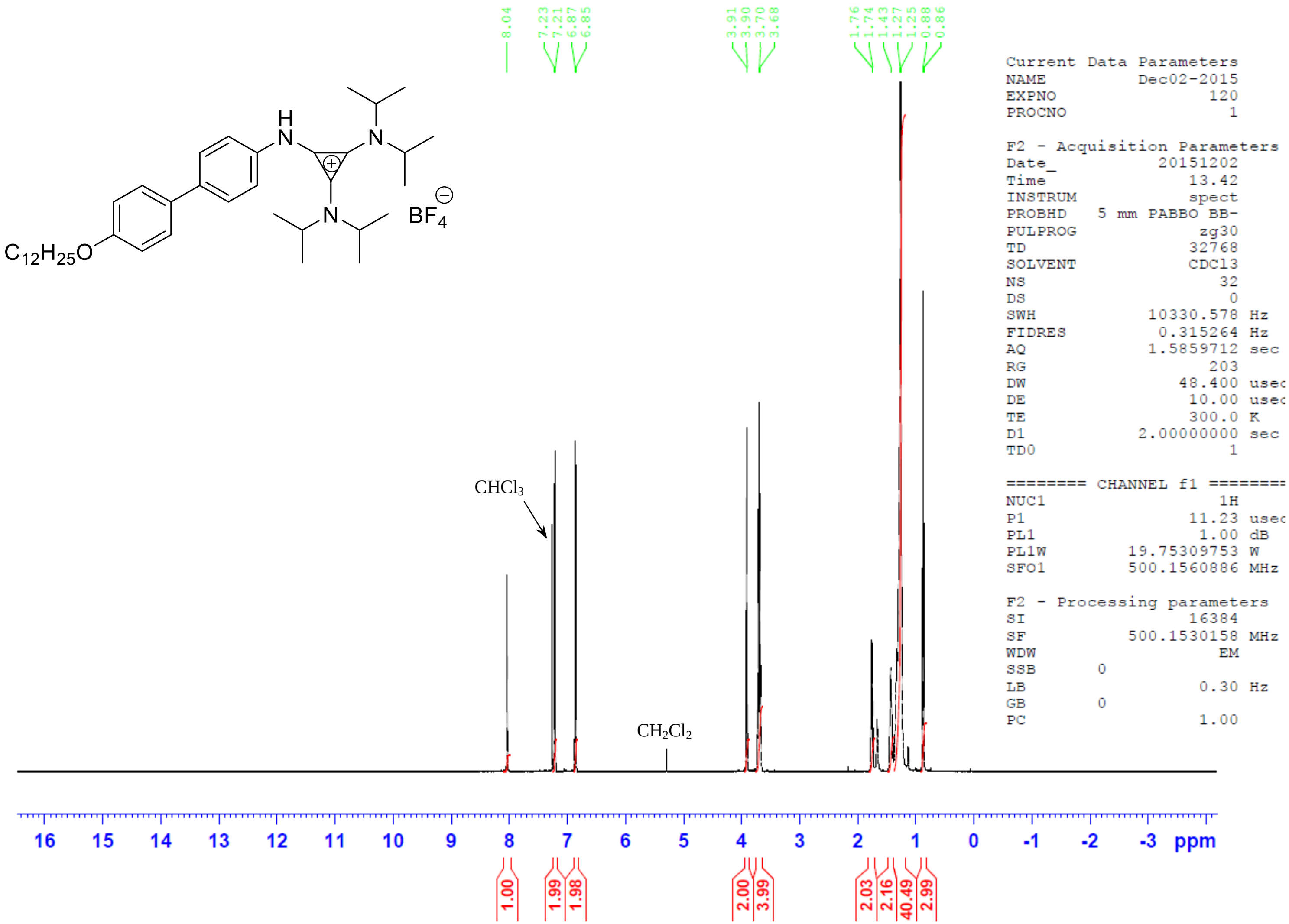

^{1}H NMR spectrum of **8a**.

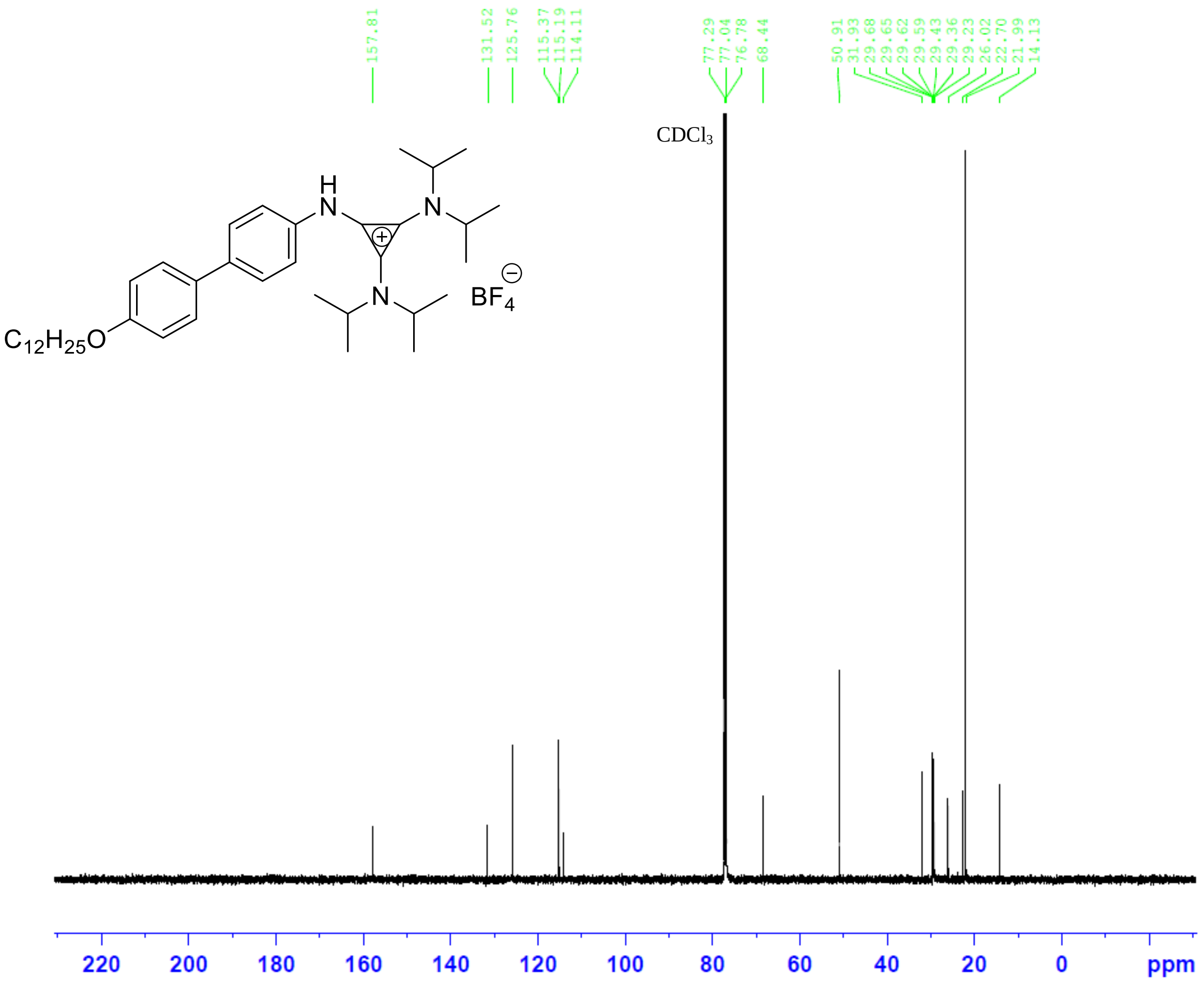


```
Current Data Parameters
NAME            Dec02-2015
EXPNO                  121
PROCNO                   1

F2 - Acquisition Parameters
Date_             20151202
Time                 14.35
INSTRUM              spect
PROBHD   5 mm PABBO BB-
PULPROG            zgpg30
TD                   65536
SOLVENT              CDCl3
NS                    1024
DS                       4
SWH             32894.738 Hz
FIDRES           0.501934 Hz
AQ              0.9961472 sec
RG                    2580
DW                  15.200 use
DE                   10.00 use
TE                   296.8 K
D1              2.00000000 sec
D11             0.03000000 sec
TD0                      1

======== CHANNEL f1 =======
NUC1                   13C
P1                   10.20 use
PL1                   1.50 dB
PL1W           51.74793243 W
SFO1          125.7761482 MHz

======== CHANNEL f2 =======
CPDPRG[2           waltz16
NUC2                    1H
PCPD2               100.00 use
PL2                   1.00 dB
PL12                 19.99 dB
PL13                 21.00 dB
PL2W           19.75309753 W
PL12W           0.24925002 W
PL13W           0.19753097 W
SFO2          500.1550006 MHz
```

^{13}C NMR spectrum of **8a**.

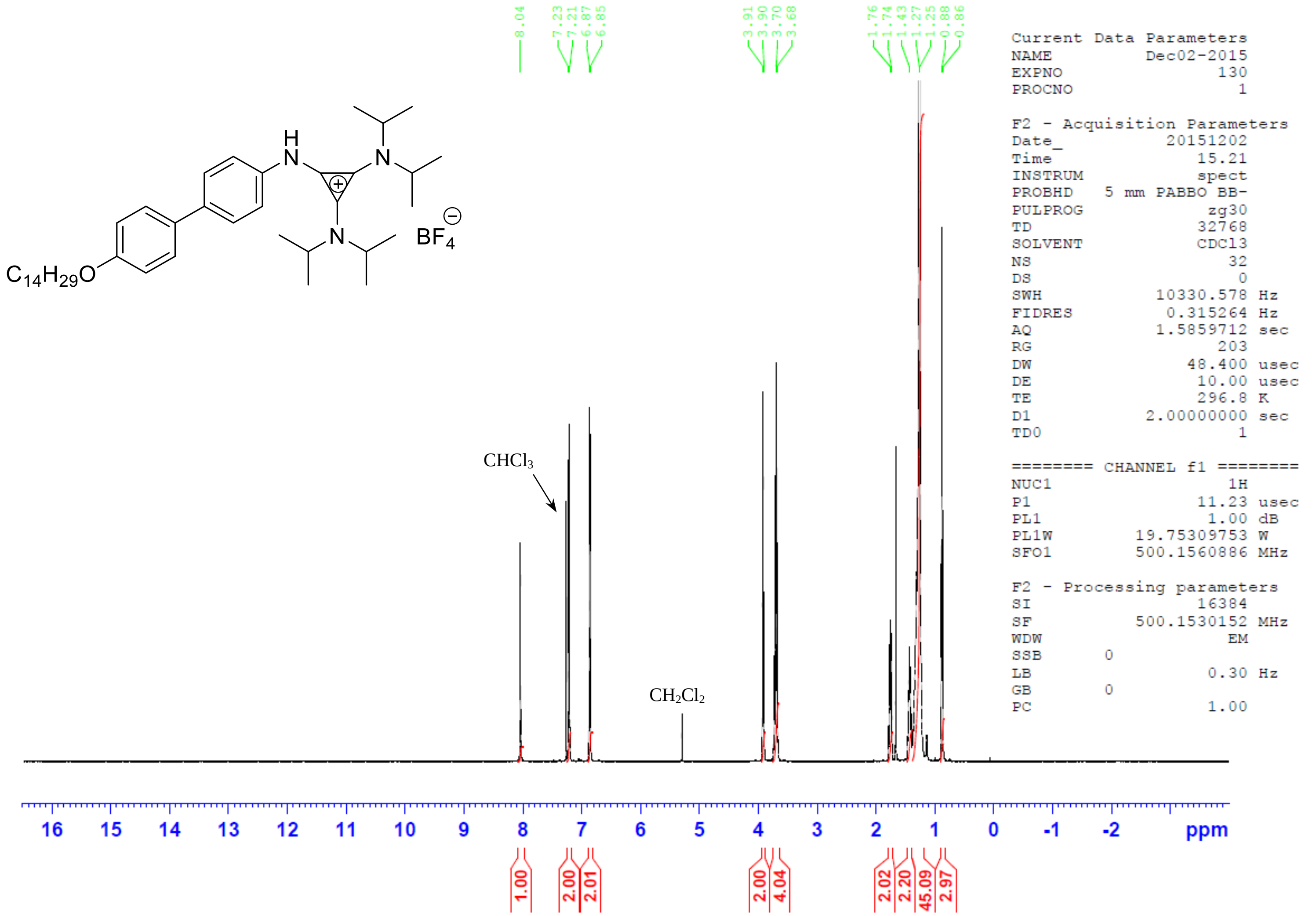

^{1}H NMR spectrum of **8b**.

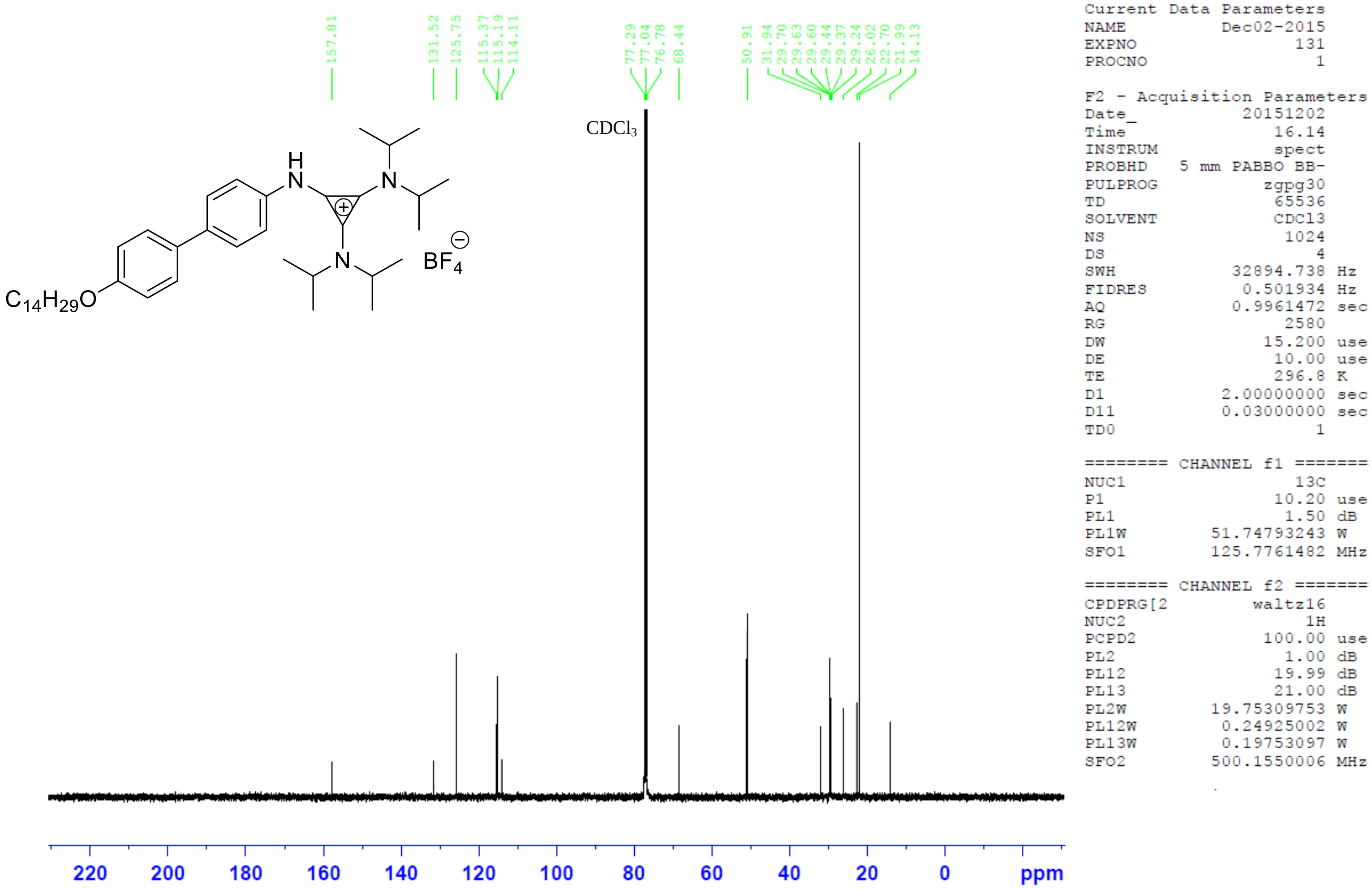

^{13}C NMR spectrum of **8b**.

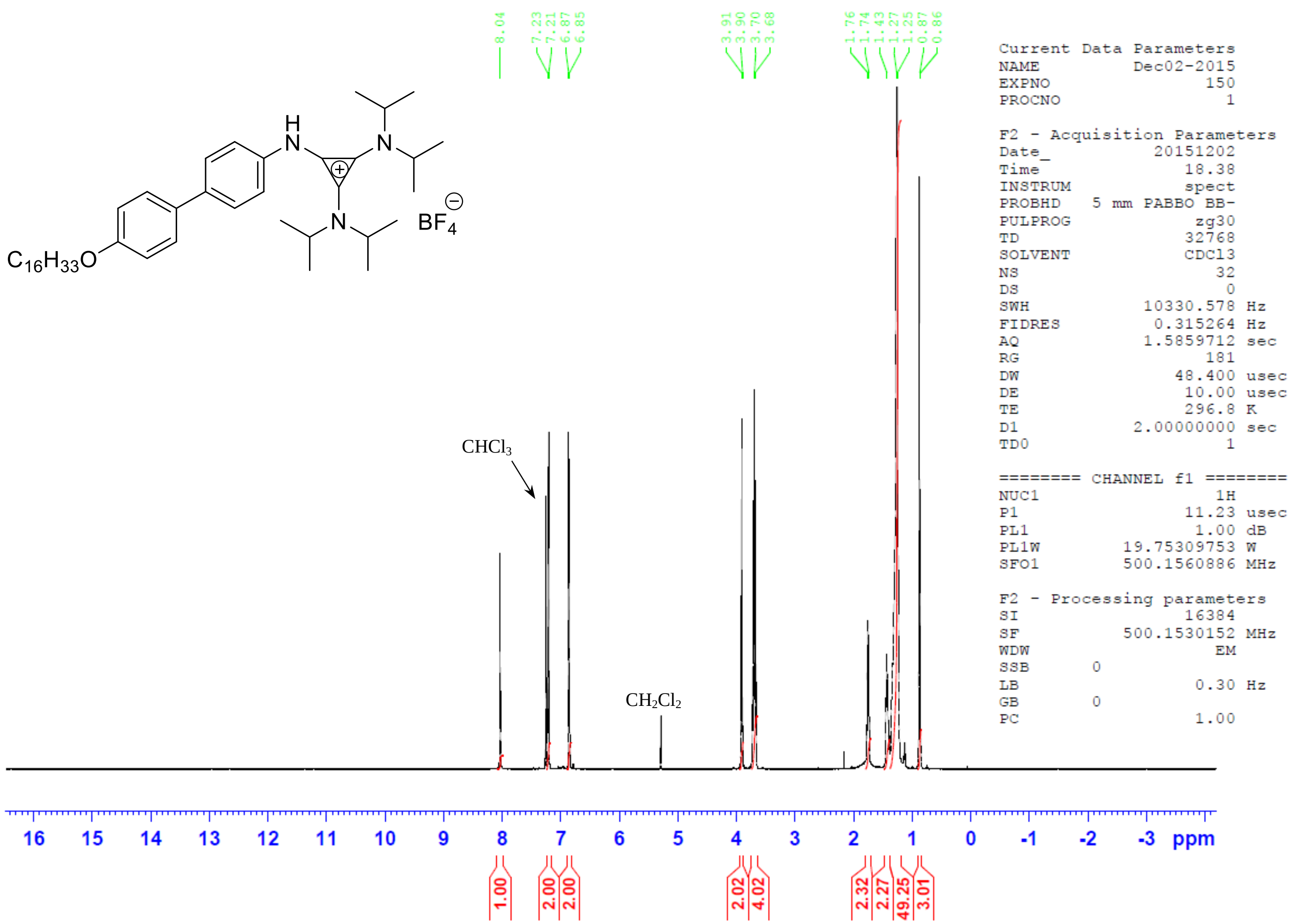

^{1}H NMR spectrum of **8c**.

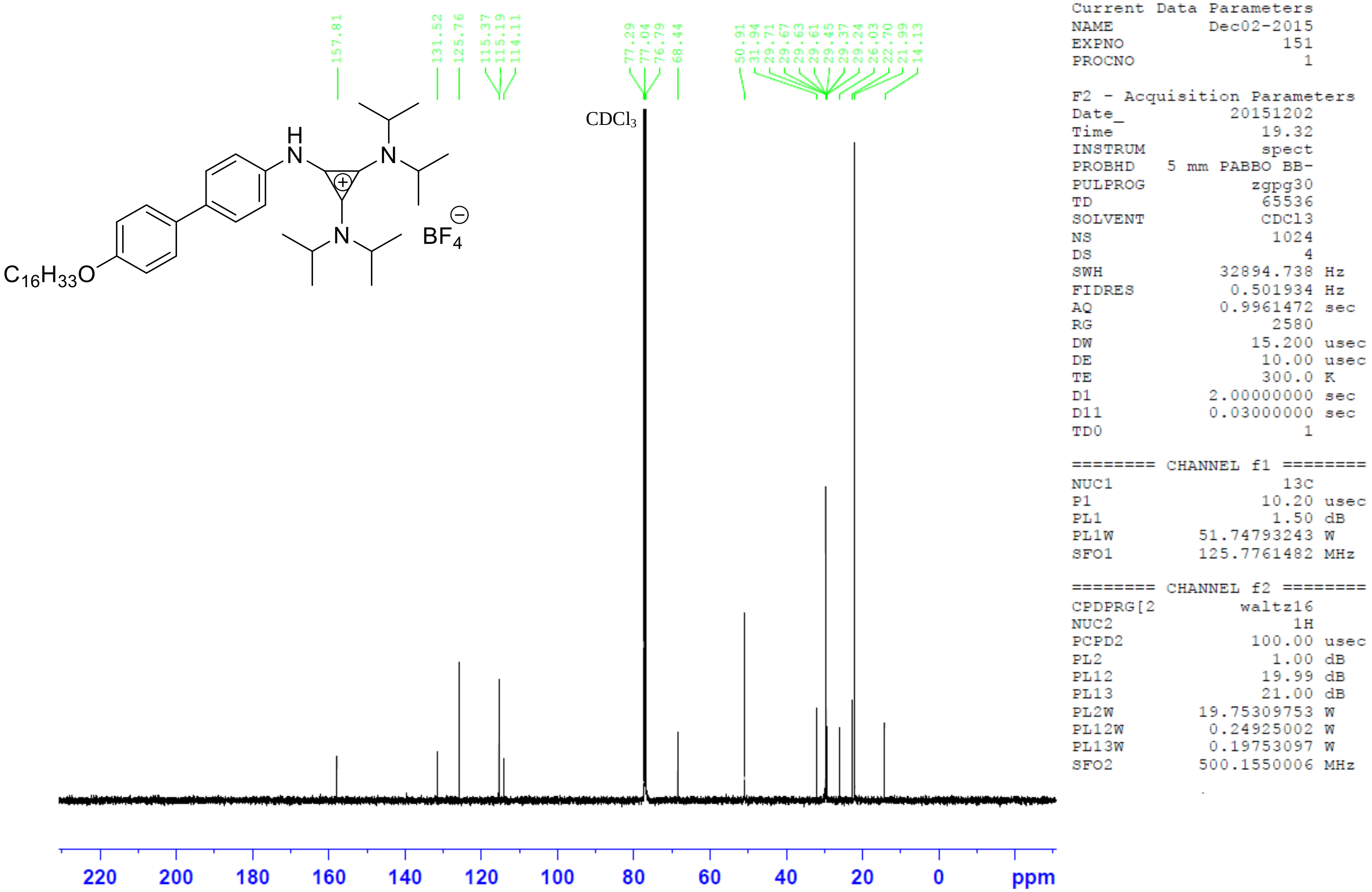

^{13}C NMR spectrum of **8c**.

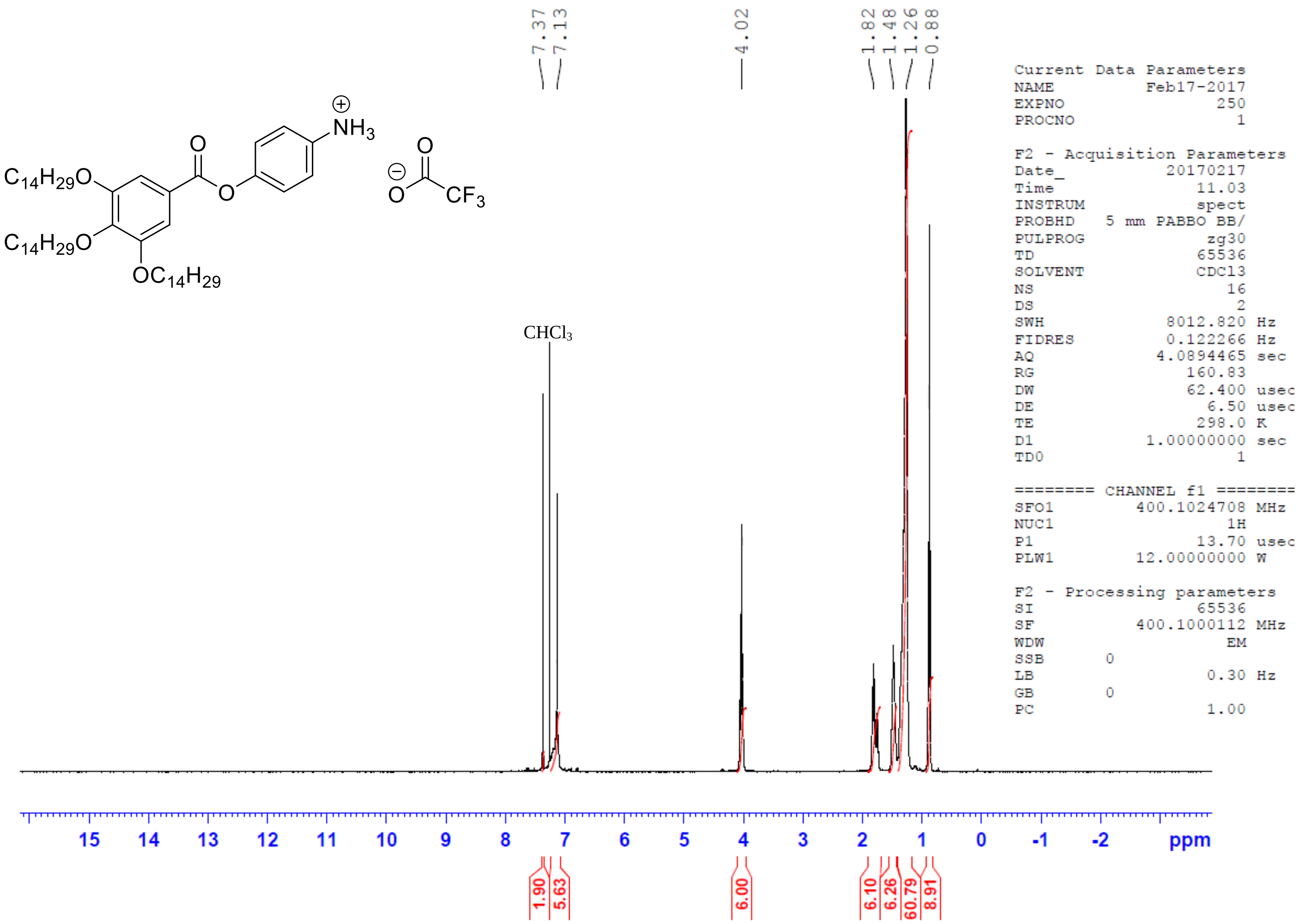

^{1}H NMR spectrum of **9**.

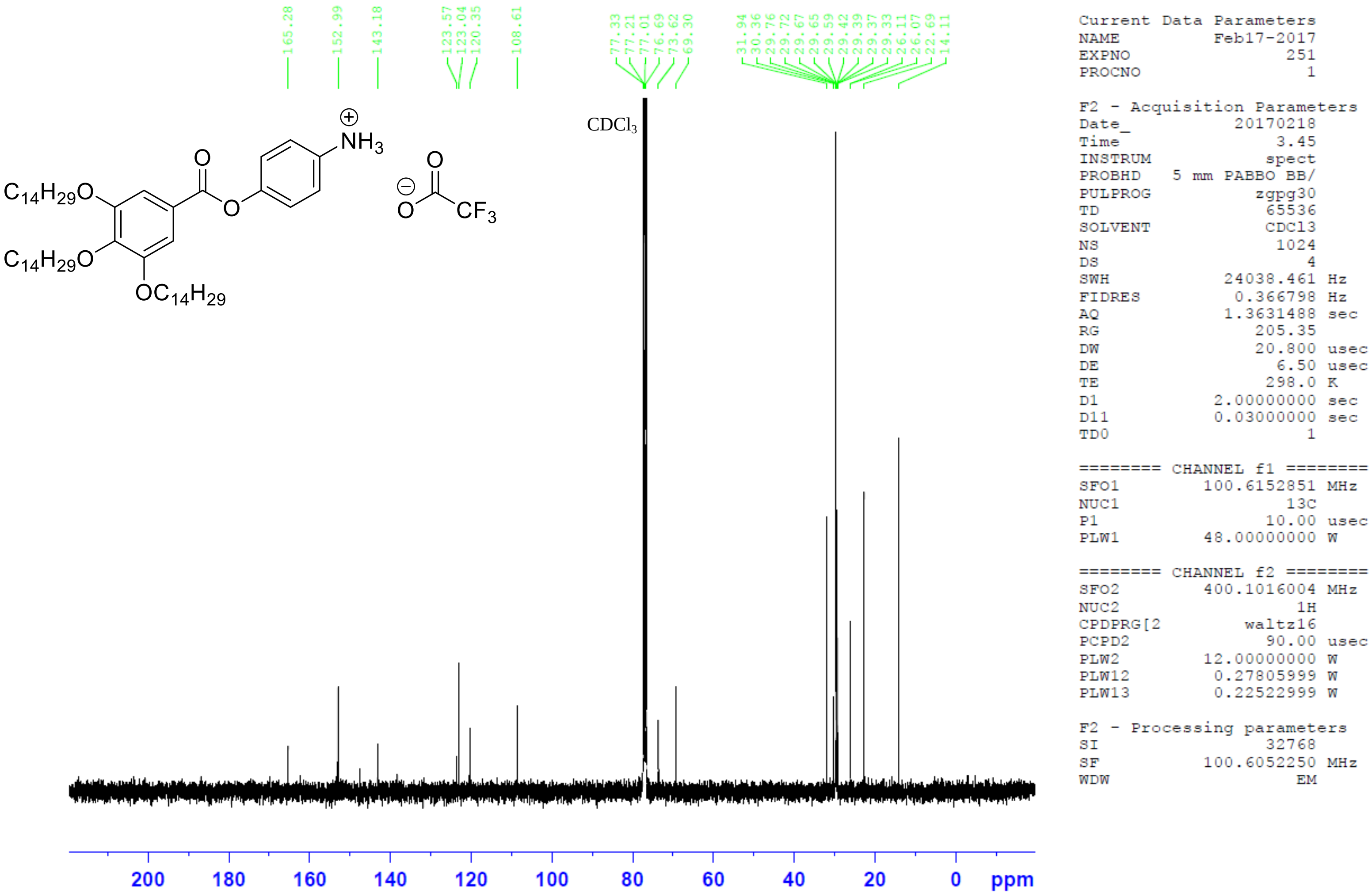

^{13}C NMR spectrum of **9**.

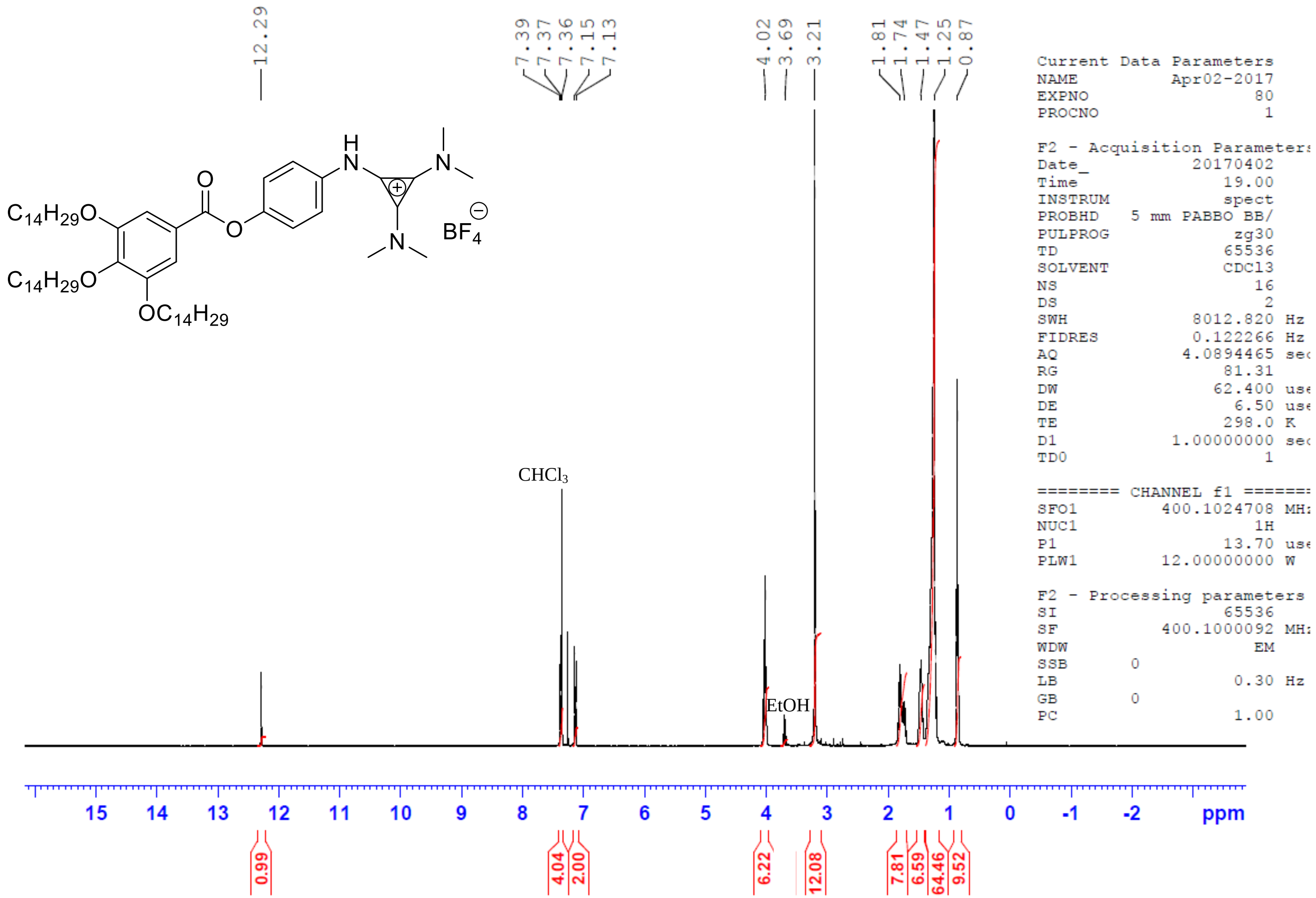

^{1}H NMR spectrum of **10a**.

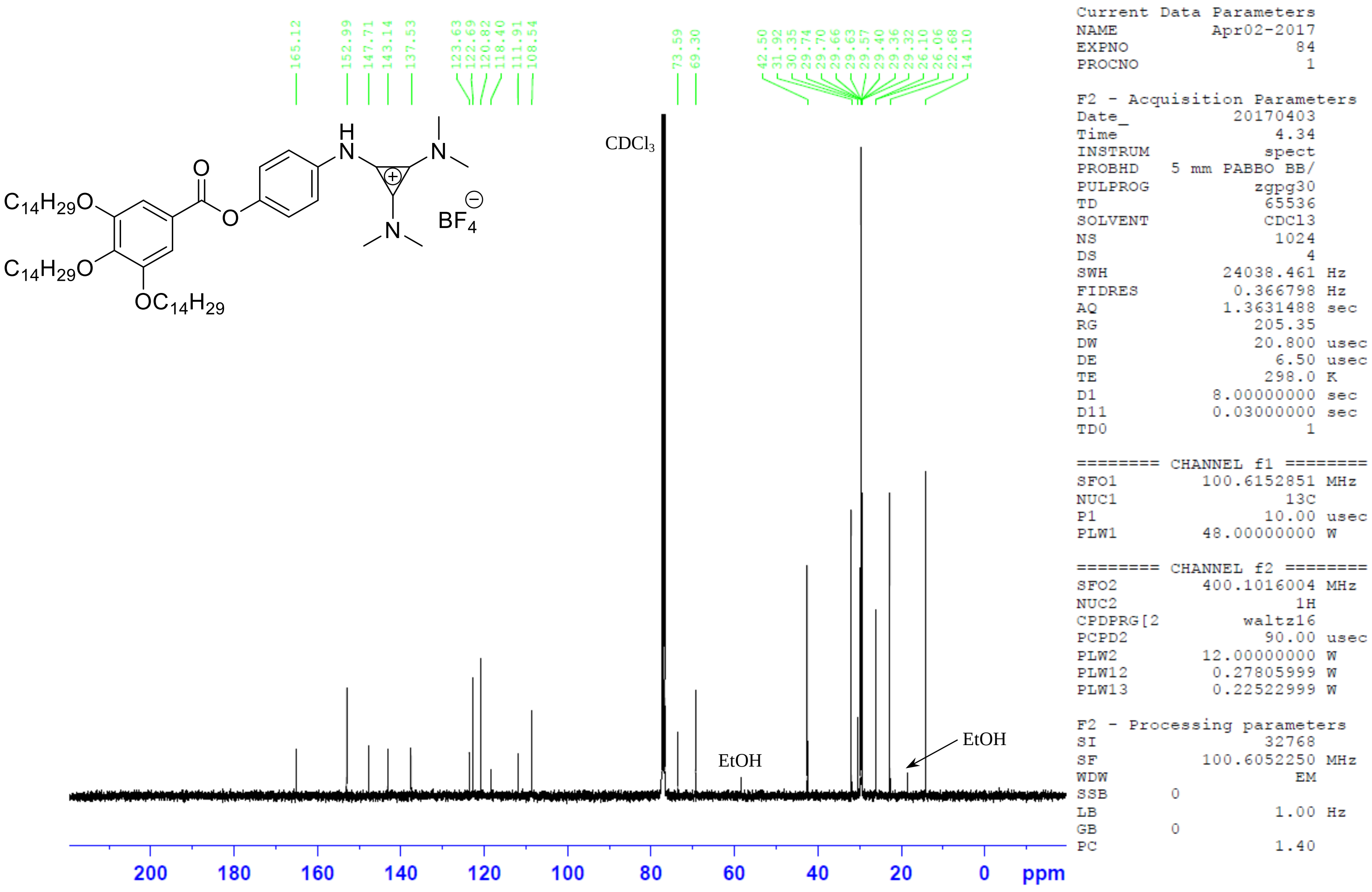

^{13}C NMR spectrum of **10a**.

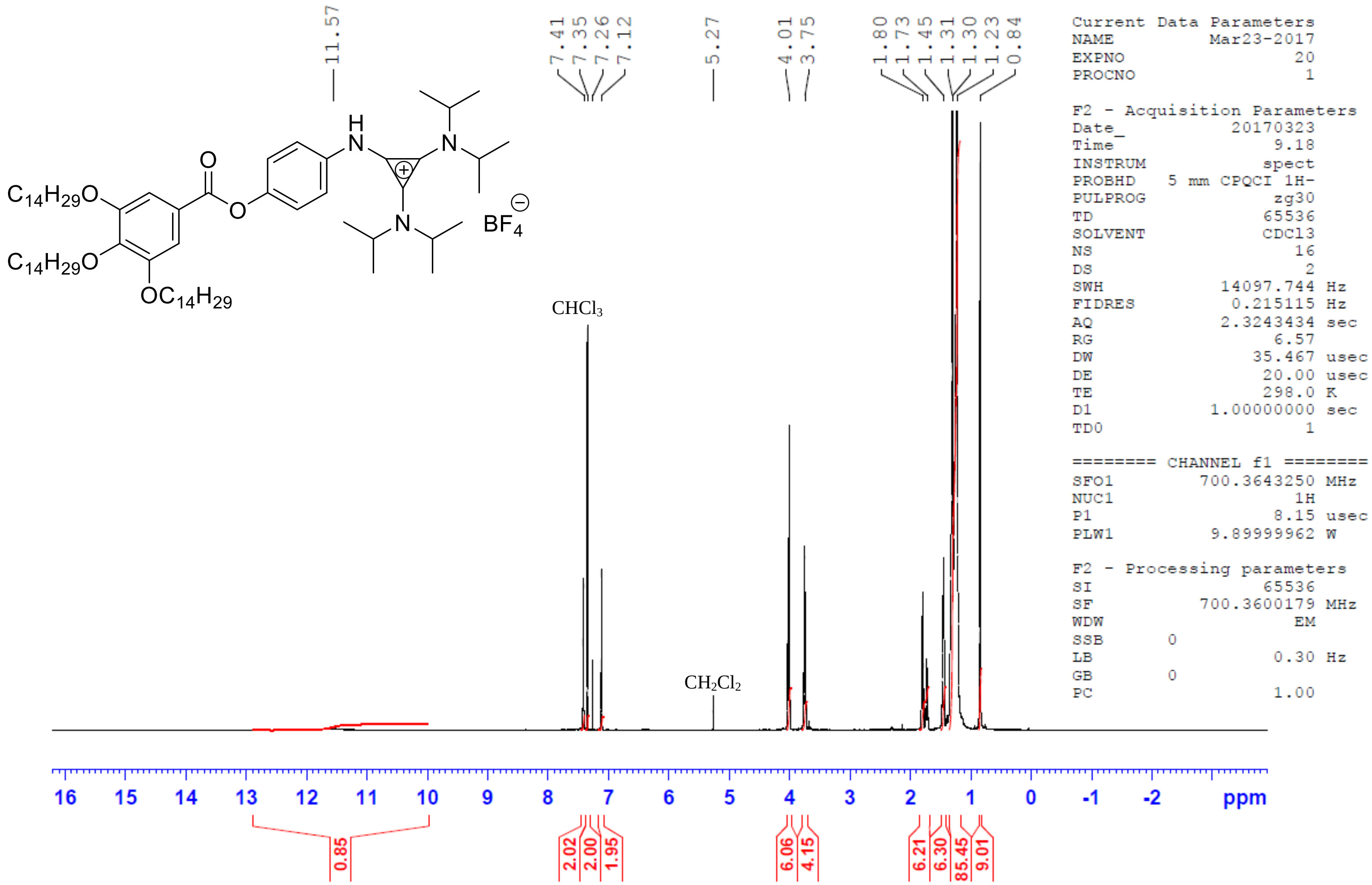

^{1}H NMR spectrum of **10b**.

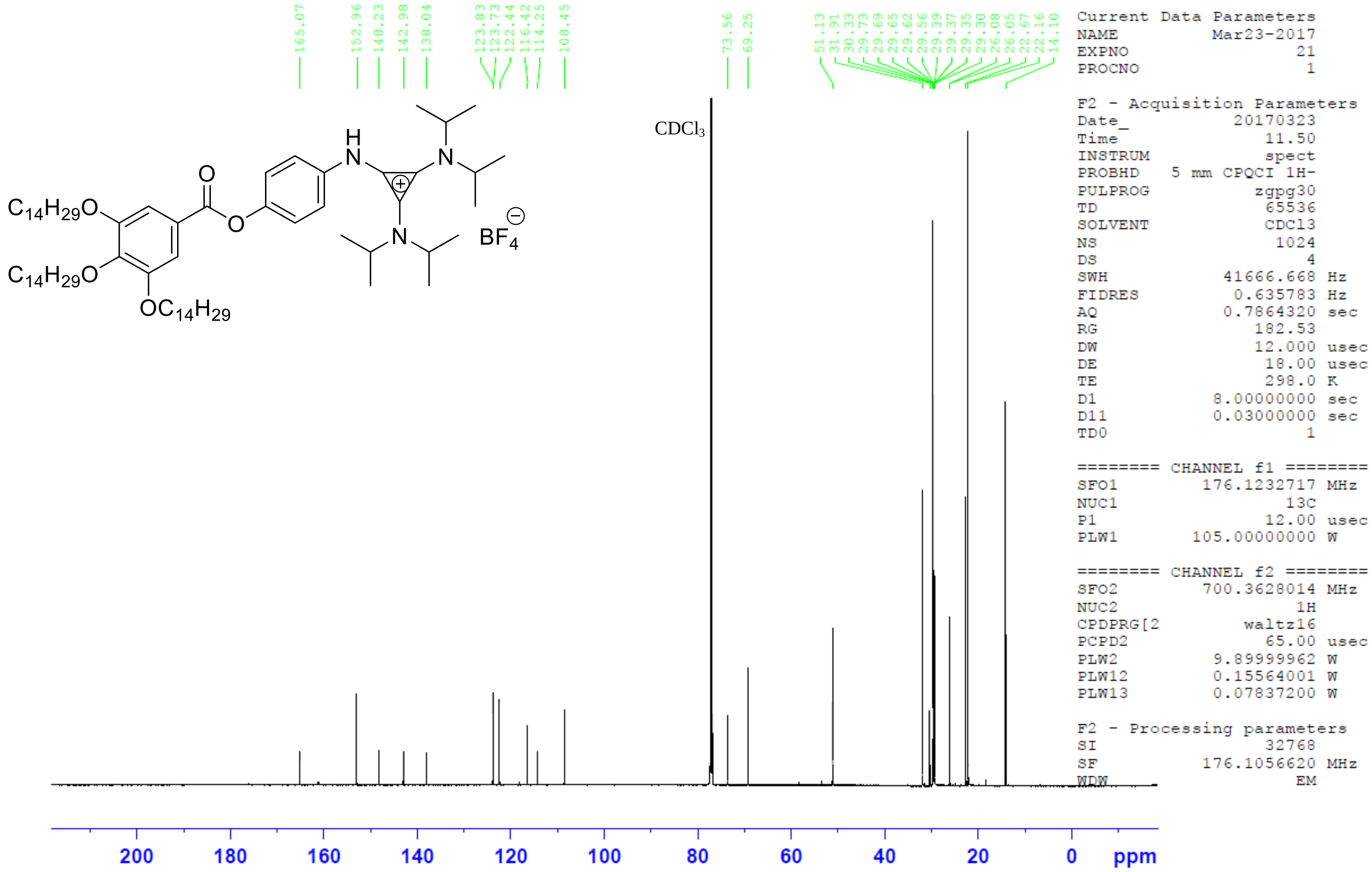

^{13}C NMR spectrum of **10b**.

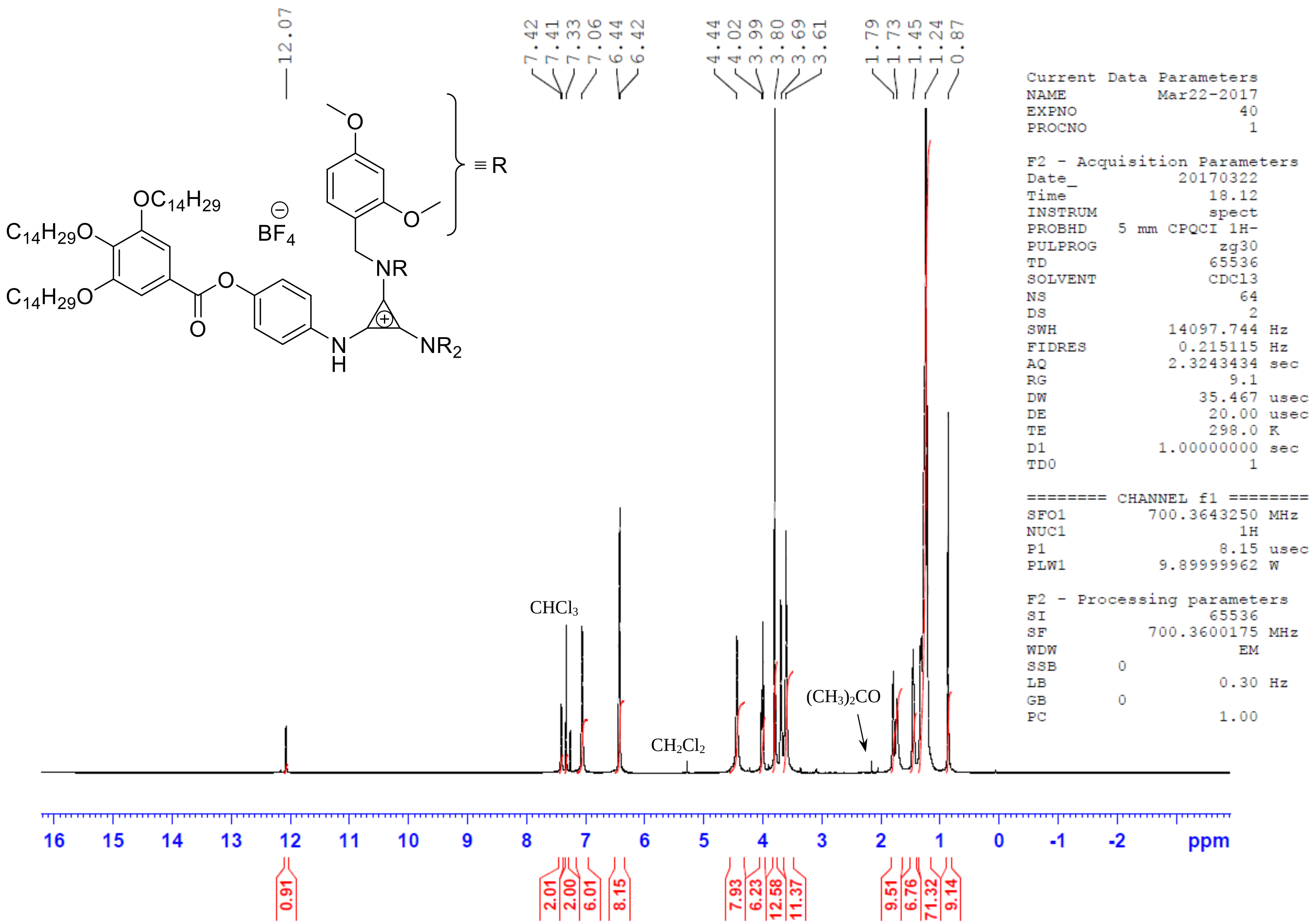

^{1}H NMR spectrum of **10c**.

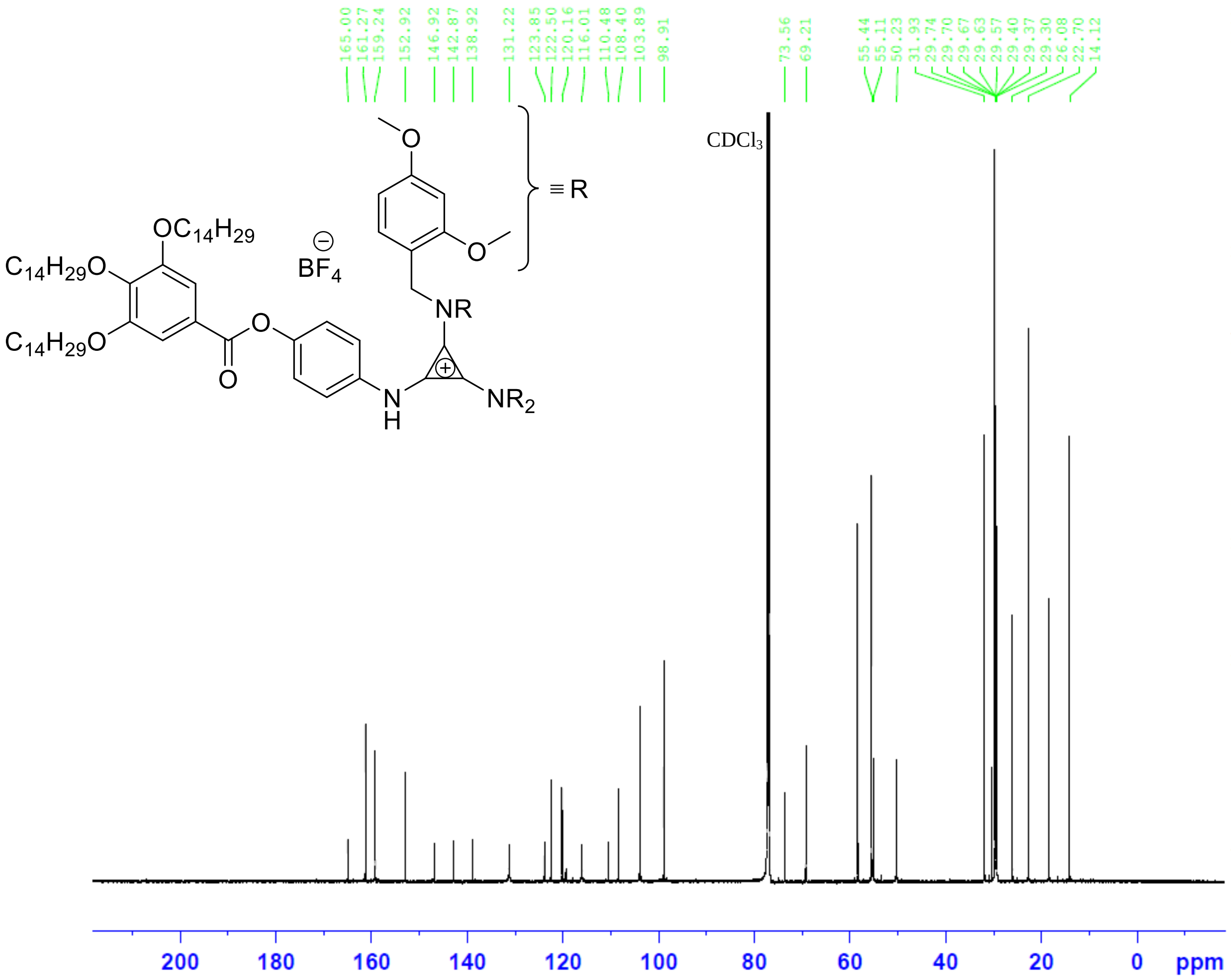


```
Current Data Parameters
NAME            Mar22-2017
EXPNO                   41
PROCNO                   1

F2 - Acquisition Parameters
Date_             20170322
Time                 20.44
INSTRUM              spect
PROBHD    5 mm CPQCI 1H-
PULPROG            zgpg30
TD                   65536
SOLVENT              CDCl3
NS                    1024
DS                       4
SWH              41666.668 Hz
FIDRES            0.635783 Hz
AQ               0.7864320 sec
RG                  182.53
DW                  12.000 usec
DE                   18.00 usec
TE                   298.0 K
D1              8.00000000 sec
D11             0.03000000 sec
TD0                      1

======== CHANNEL f1 ========
SFO1          176.1232717 MHz
NUC1                  13C
P1                  12.00 usec
PLW1         105.00000000 W

======== CHANNEL f2 ========
SFO2          700.3628014 MHz
NUC2                   1H
CPDPRG[2          waltz16
PCPD2               65.00 usec
PLW2           9.89999962 W
PLW12          0.15564001 W
PLW13          0.07837200 W

F2 - Processing parameters
SI                  32768
SF            176.1056620 MHz
WDW                    EM
```

^{13}C NMR spectrum of **10c**.

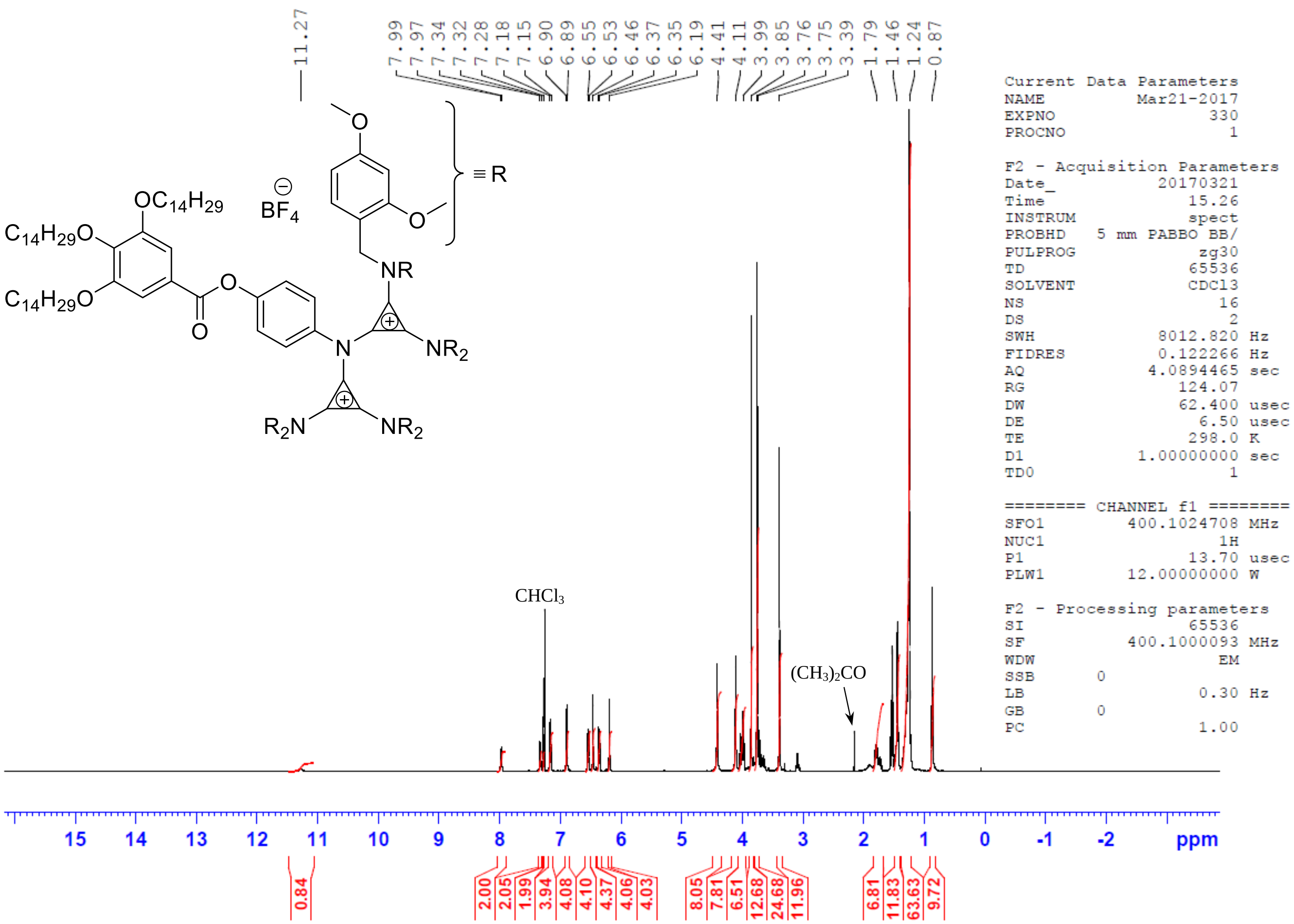

^{1}H NMR spectrum of **11**.

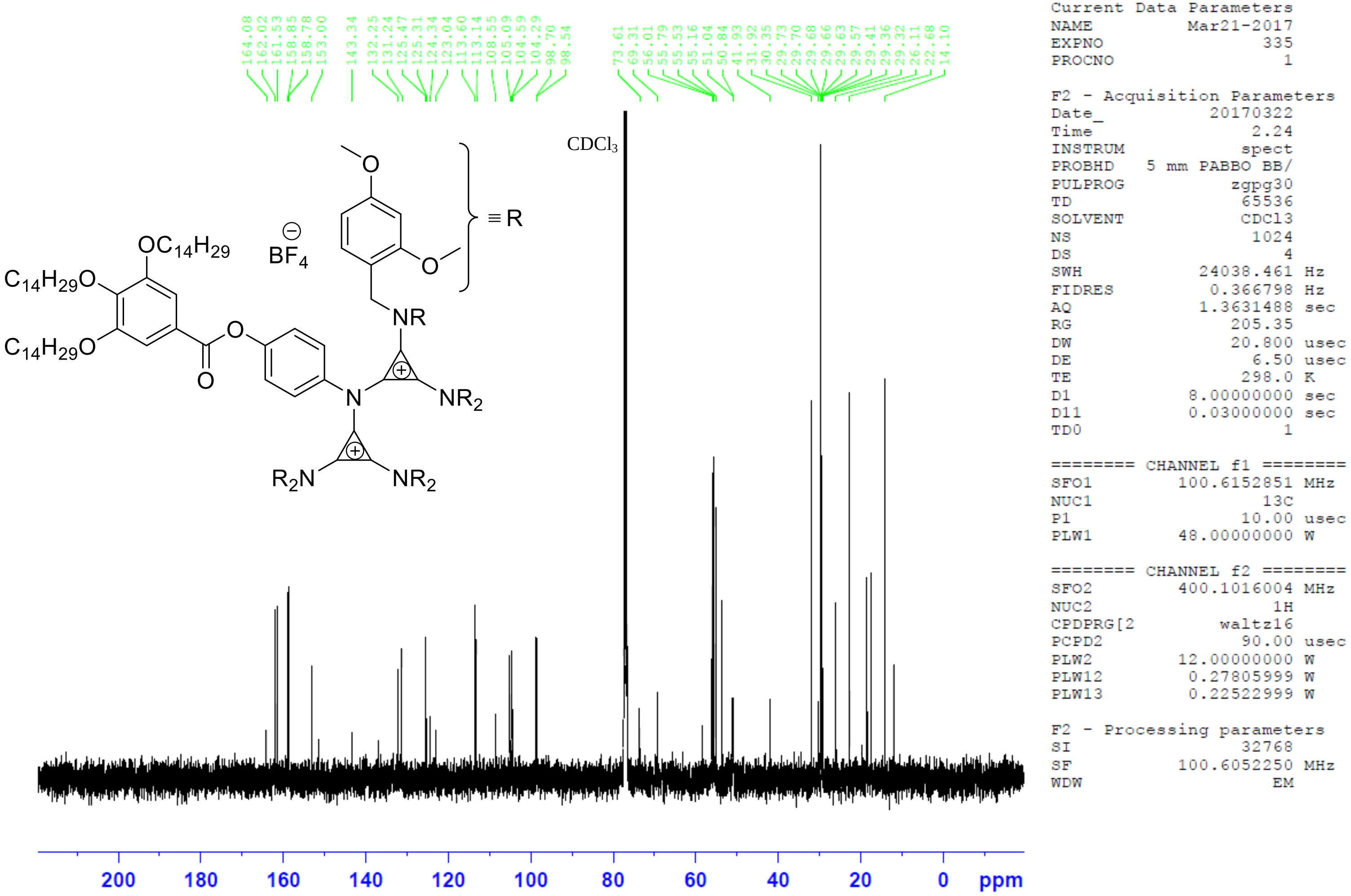

^{13}C NMR spectrum of **11**.

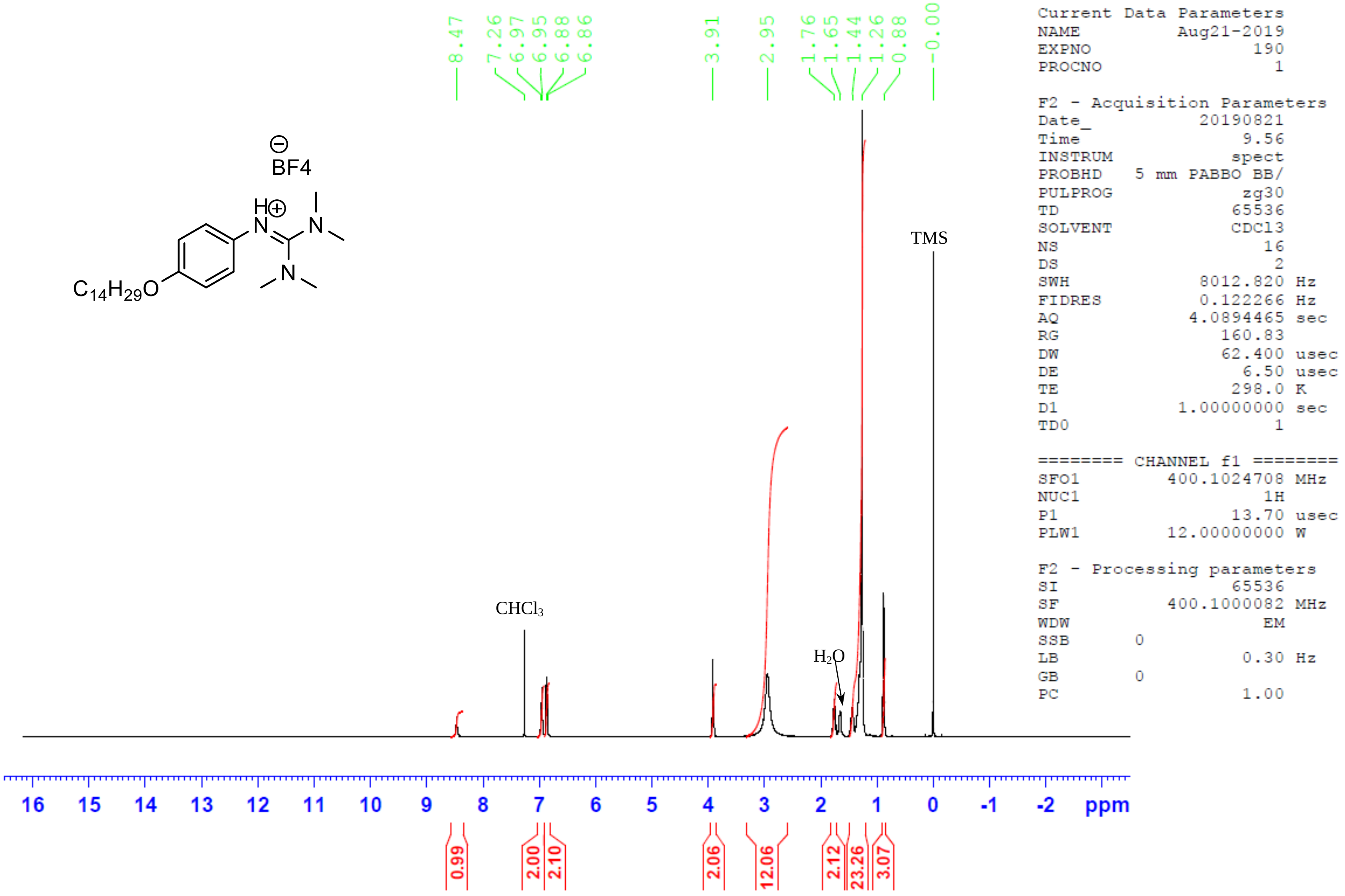

^{1}H NMR spectrum of **12a**.

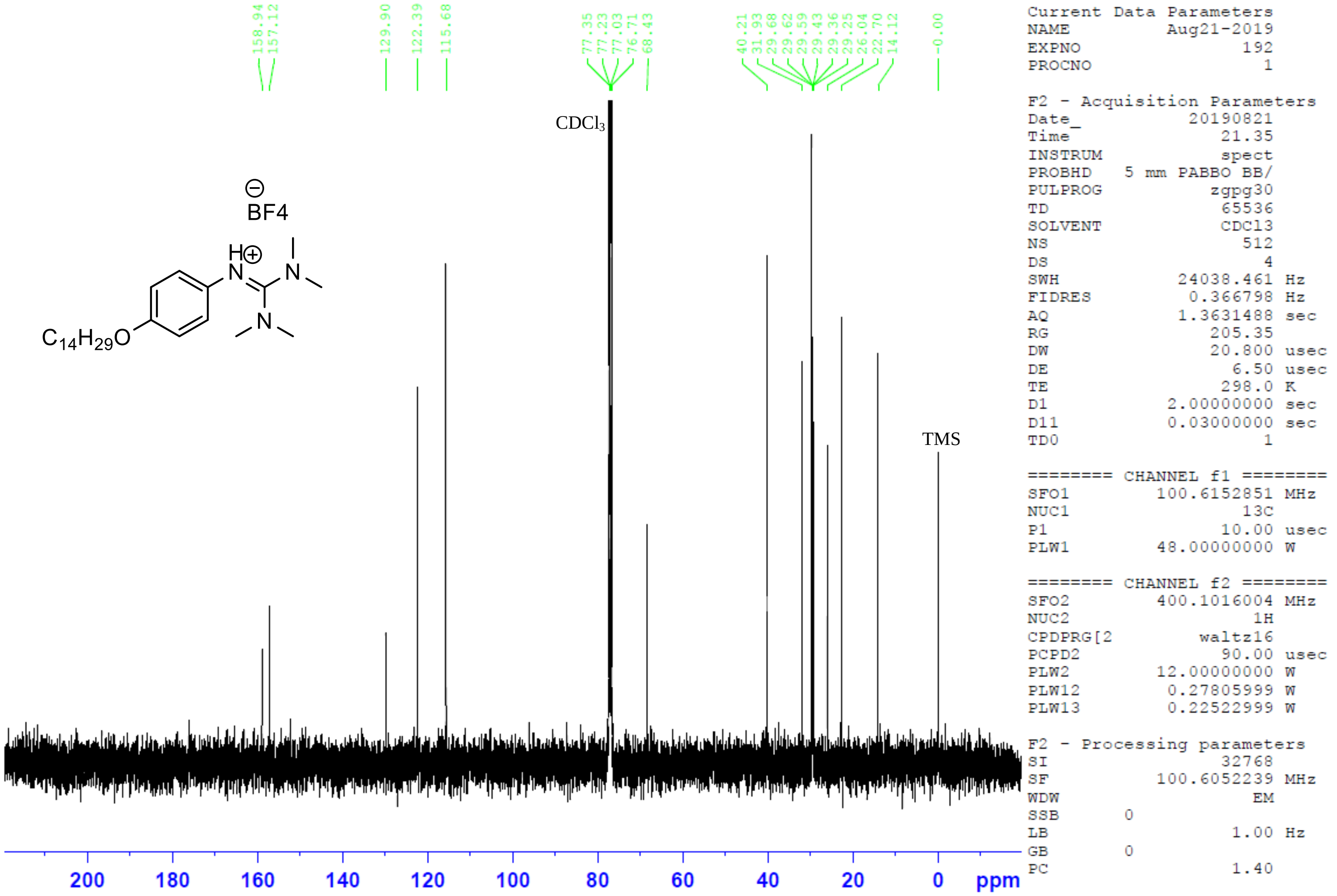

^{13}C NMR spectrum of **12a**.

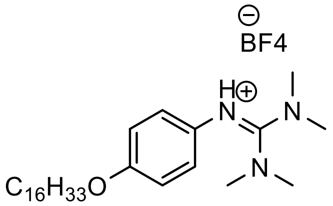

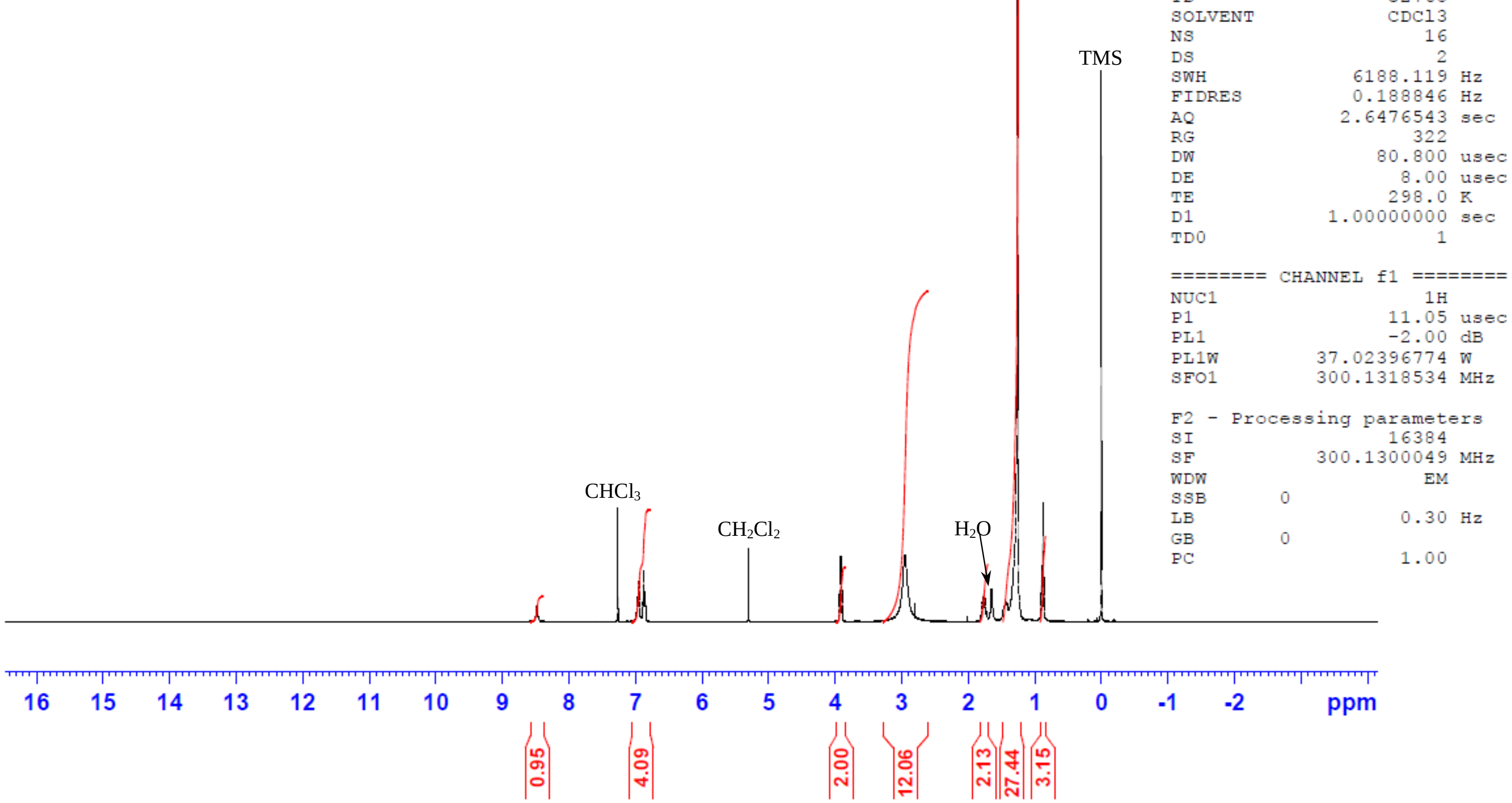

^{1}H NMR spectrum of **12b**.

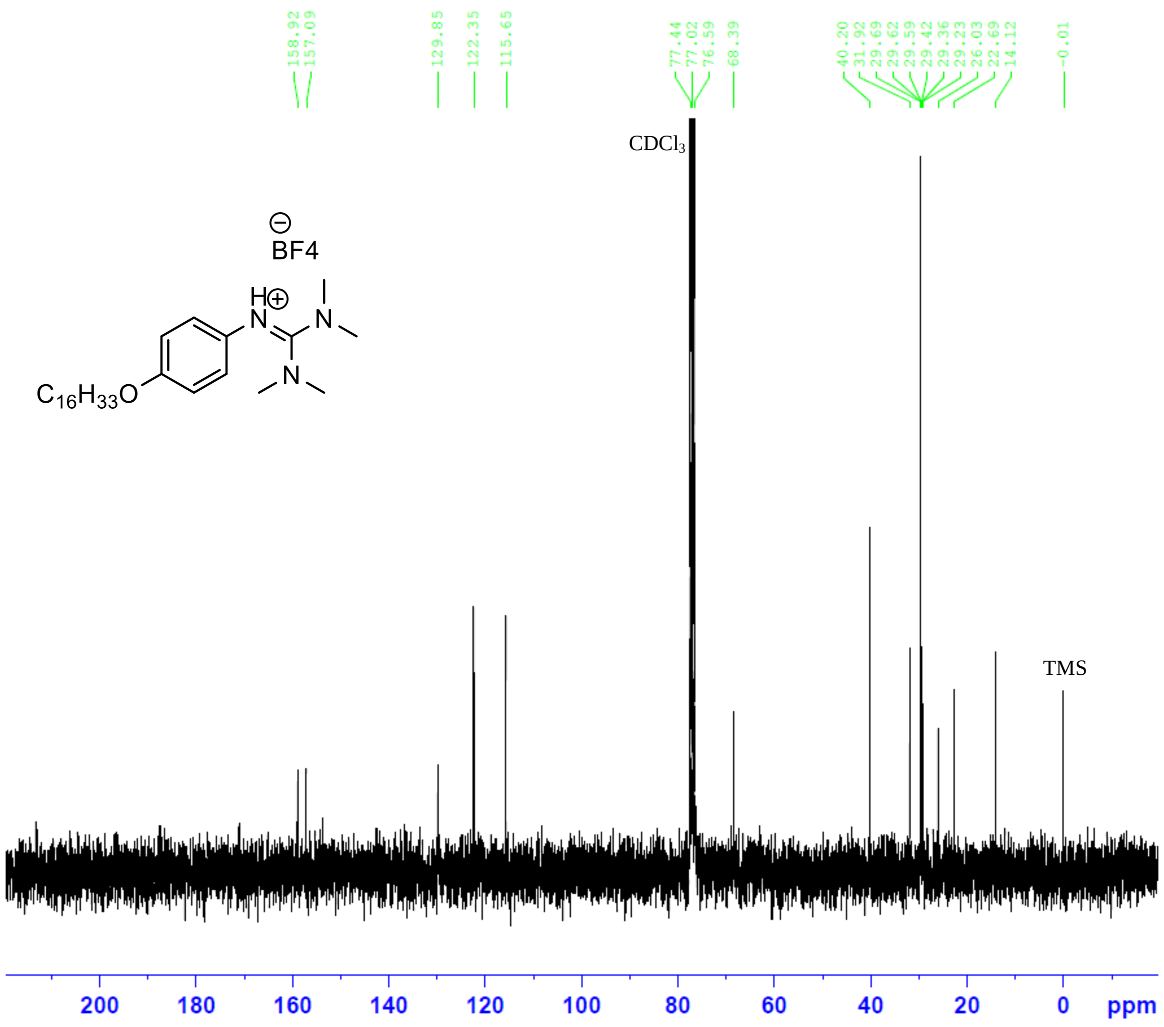


```
Current Data Parameters
NAME          Aug22-2019
EXPNO                501
PROCNO                 1

F2 - Acquisition Parameters
Date_           20190822
Time               18.29
INSTRUM            spect
PROBHD   5 mm PABBO BB-
PULPROG          zgpg30
TD                 65536
SOLVENT            CDCl3
NS                   512
DS                     4
SWH            18028.846 Hz
FIDRES          0.275098 Hz
AQ             1.8175317 sec
RG                  1620
DW                27.733 usec
DE                  8.00 usec
TE                 298.0 K
D1            2.00000000 sec
D11           0.03000000 sec
TD0                    1

======== CHANNEL f1 ========
NUC1                 13C
P1                 11.75 usec
PL1       0 dB
PL1W         31.14739227 W
SFO1          75.4752953 MHz

======== CHANNEL f2 ========
CPDPRG[2         waltz16
NUC2                  1H
PCPD2             100.00 usec
PL2                -2.00 dB
PL12               17.05 dB
PL13               19.00 dB
PL2W         37.02396774 W
PL12W         0.46076876 W
PL13W         0.29409182 W
SFO2         300.1312005 MHz

F2 - Processing parameters
SI                 32768
SF            75.4677490 MHz
WDW                   EM
SSB       0
LB                  1.00 Hz
GB        0
PC                  1.40
```

^{13}C NMR spectrum of **12b**.

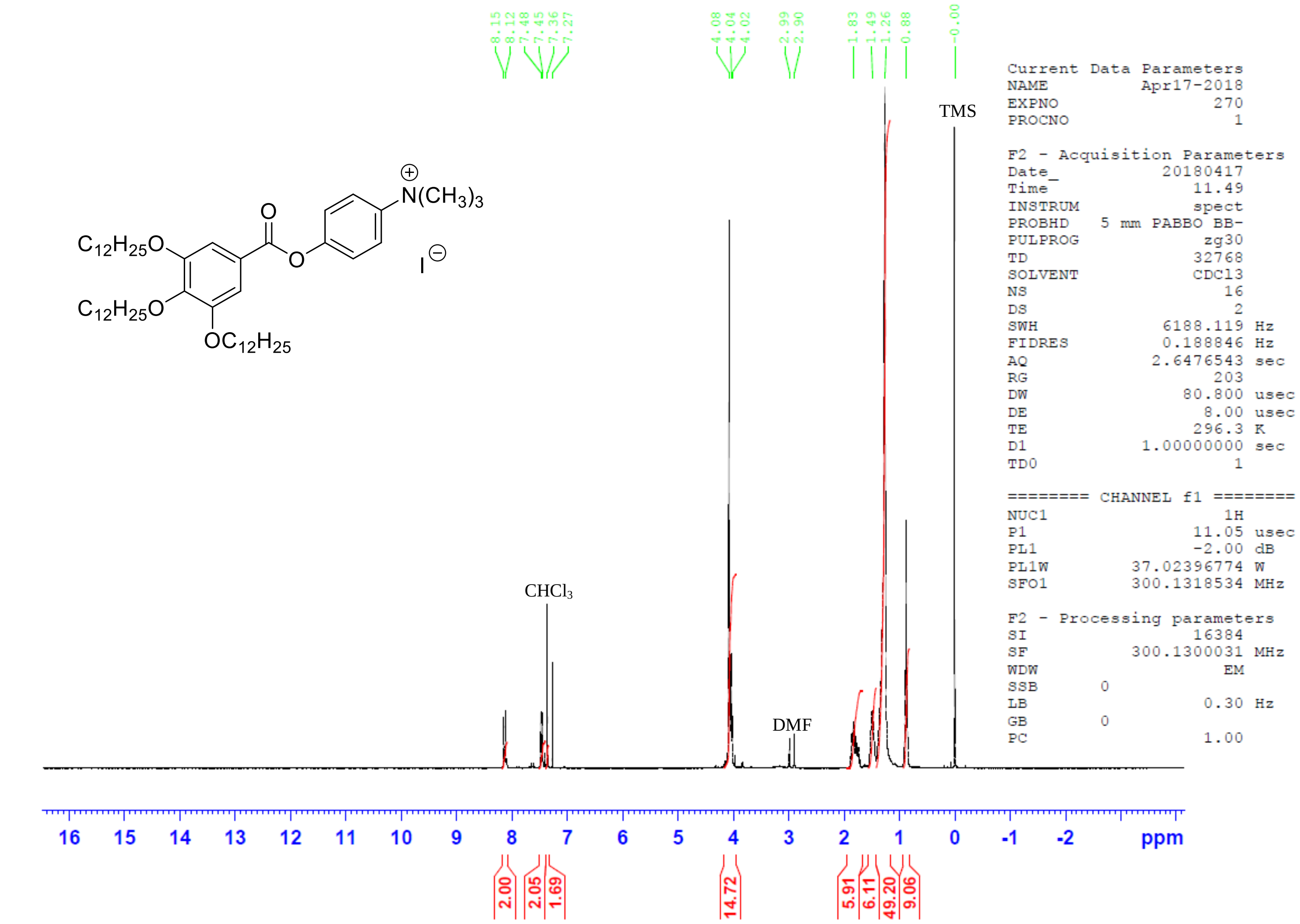

^{1}H NMR spectrum of **14**.

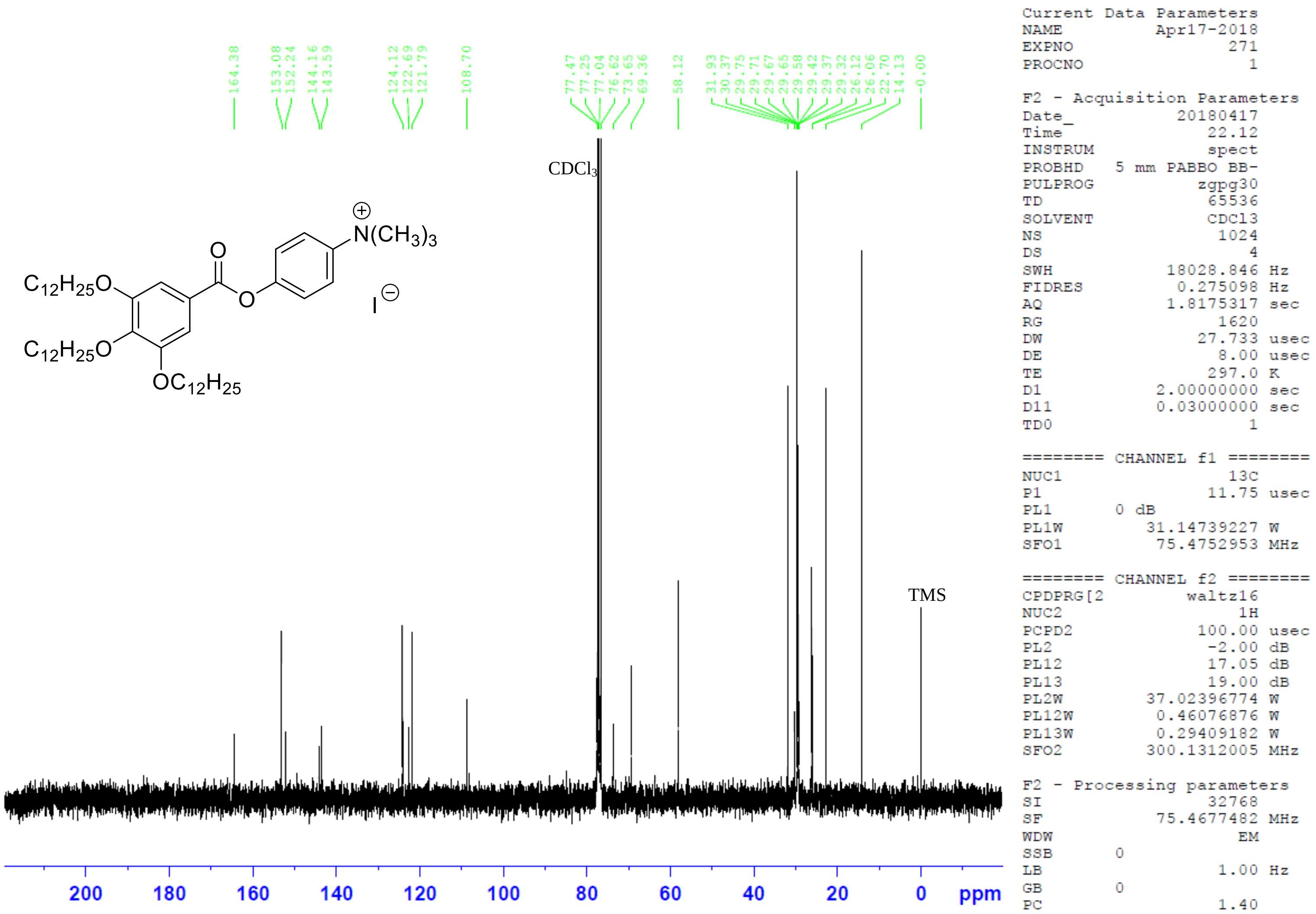

^{13}C NMR spectrum of **14**.
